# Survey of open science practices and attitudes in the social sciences

**DOI:** 10.1038/s41467-023-41111-1

**Published:** 2023-09-05

**Authors:** Joel Ferguson, Rebecca Littman, Garret Christensen, Elizabeth Levy Paluck, Nicholas Swanson, Zenan Wang, Edward Miguel, David Birke, John-Henry Pezzuto

**Affiliations:** 1 University of California, Berkeley, Department of Agricultural and Resource Economics, Berkeley, California USA; 2 University of Illinois Chicago, Department of Psychology, Chicago, Illinois USA; 3 Federal Deposit Insurance Corporation, Washington, District of Columbia USA; 4 Princeton University, Department of Psychology, Princeton, New Jersey USA; 5 University of California, Berkeley, Department of Economics, Berkeley, California USA; 6 University of California, San Diego, Rady School of Management, La Jolla, California USA

**Keywords:** Economics, Psychology, Human behaviour, Interdisciplinary studies, Sociology

## Abstract

Open science practices such as posting data or code and pre-registering analyses are increasingly prescribed and debated in the applied sciences, but the actual popularity and lifetime usage of these practices remain unknown. This study provides an assessment of attitudes toward, use of, and perceived norms regarding open science practices from a sample of authors published in top-10 (most-cited) journals and PhD students in top-20 ranked North American departments from four major social science disciplines: economics, political science, psychology, and sociology. We observe largely favorable private attitudes toward widespread lifetime usage (meaning that a researcher has used a particular practice at least once) of open science practices. As of 2020, nearly 90% of scholars had ever used at least one such practice. Support for posting data or code online is higher (88% overall support and nearly at the ceiling in some fields) than support for pre-registration (58% overall). With respect to norms, there is evidence that the scholars in our sample appear to underestimate the use of open science practices in their field. We also document that the reported lifetime prevalence of open science practices increased from 49% in 2010 to 87% a decade later.

## Abstract (web summary)

Open science practices are becoming more common in the social sciences, but there is limited data on their popularity and prevalence. Here, using survey data, the authors provide evidence that levels of adoption are relatively high and underestimated by many in the field.

## Introduction

Open science practices—such as posting data or code in online archives and pre-registering hypotheses and analyses—have been the subject of controversy in the social sciences^1–6^. While there have been institutional and technological innovations that have promoted open science practices in recent years^7,8^, there has also been noticeable individual and field-based opposition to at least some practices^3,9–11^. Debates about whether these practices improve or inhibit scientific progress, creativity, and rigor to date have not, unfortunately, proceeded from a basic understanding of how widespread these practices are, how popular they are from the perspective of social scientists’ personal views, and how much these practices have already become part of the normative cultures of various social science fields.

Previous research projects have attempted to answer this important question, but most have provided a partial or possibly unrepresentative picture^12–17^. For example, refs. ^18,19^ use an audit approach to explore the point prevalence of open science practices used in randomly sampled articles, finding generally low usage. While this approach provides a useful snapshot, their article-level methodology cannot speak to the lifetime adoption of open science practices by researchers or social scientists’ opinions on open science. Additionally, some previous studies have used relatively narrow definitions of open science practices, for example, only counting a researcher as engaging in the practice if they post data or submit a pre-registration in specific locations mentioned in the article body^19^.

In this project, we conduct a survey of a sample of social scientists, which includes scholars published in top-10 journals and graduate students in top-20 ranked North American departments, across four major fields: economics, psychology, political science, and sociology. We offered high levels of remuneration for participating in the survey and fielded it twice over the course of two years to ensure a decent response rate across fields. We measure scholars’ self-reported lifetime use of (meaning that a scholar has used a given practice at least once) and private attitudes toward open science practices, and their perceptions of their field’s norms regarding these practices. In particular, our survey focuses on two core open science practices: the posting of data or code and the pre-registration of hypotheses or analyses. We allow for a broad definition of both practices, not putting restrictions on how or where data was posted or pre-registrations were submitted. To understand how actual behavior aligned with private attitudes and perceived norms, we verify self-reported behavior with both web-scraping and manual audits of public websites. The present data also assess how accurately social scientists perceive open science adoption and support—i.e., whether perceived norms reflect the private consensus and behavioral trends in each field. Based on these methodological improvements, this study provides a more up-to-date and complete picture of the lifetime prevalence of and support for open science practices, as well as the extent to which reception of open science practices has varied across different age cohorts of social scientists, and across social science disciplines and methodological approaches, within our sample of respondents.

## Results

### Current open science attitudes and practices

**Fig. 1:**
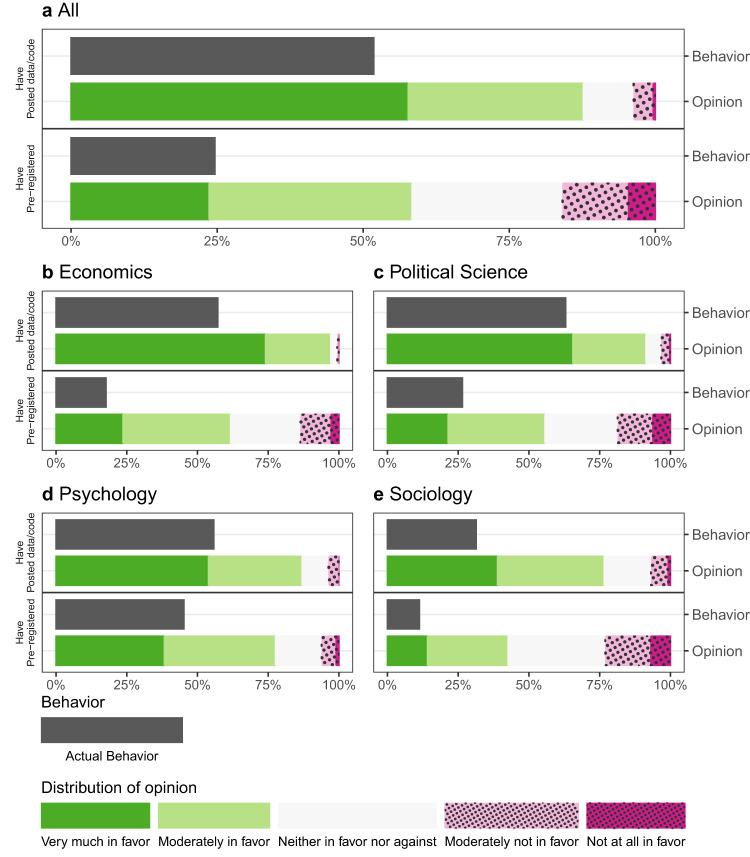
Support for and engagement in open science by discipline. Bars depict support for and reported lifetime usage of two open science practices: posting data or code online and pre-registering hypotheses or analyses. Within each panel, the solid black bar shows the proportion of researchers who report ever using the stated practice in the survey. Below each solid black bar, the next bar in the panel shows the distribution of support for the practice elicited in the survey. Panel **a** shows data from all respondents, and Panels **b**–**e** show responses from researchers identified as economists, political scientists, psychologists, and sociologists, respectively.

Figure 1 depicts the lifetime usage rates and distribution of support for open science practices for the entire sample. Lifetime usage of open science practices, on average, is high. Figure 1 depicts the self-reported usage, verified as 80% accurate by our manual audit of actual behavior. Open science practices are higher among published authors than among graduate students (Supplementary Figs. 23 and 24), though this is possibly because published authors have had more opportunities to apply open science practices to their work. There is variability in lifetime usage across fields, but rates are over 45% among all disciplines for posting data or code. Overall, the gap between the proportion of social science scholars reporting ever posting data or code (52%) and those reporting ever pre-registering hypotheses (25%) is large, though less so in psychology.

The survey also reveals high levels of favorability toward open science practices across all four disciplines and to the same extent among both published authors and graduate students. In response to questions such as “To what extent do you believe that [PRACTICE] is important for progress in [DISCIPLINE]?”, over half of all social scientists were very much or moderately in favor of pre-registration; over 85% felt this way with respect to posting data or code. It is notable that there are fairly high levels of stated support for open science, even among scholars in a discipline like sociology, where there is less institutionalization of these practices.

**Fig. 2:**
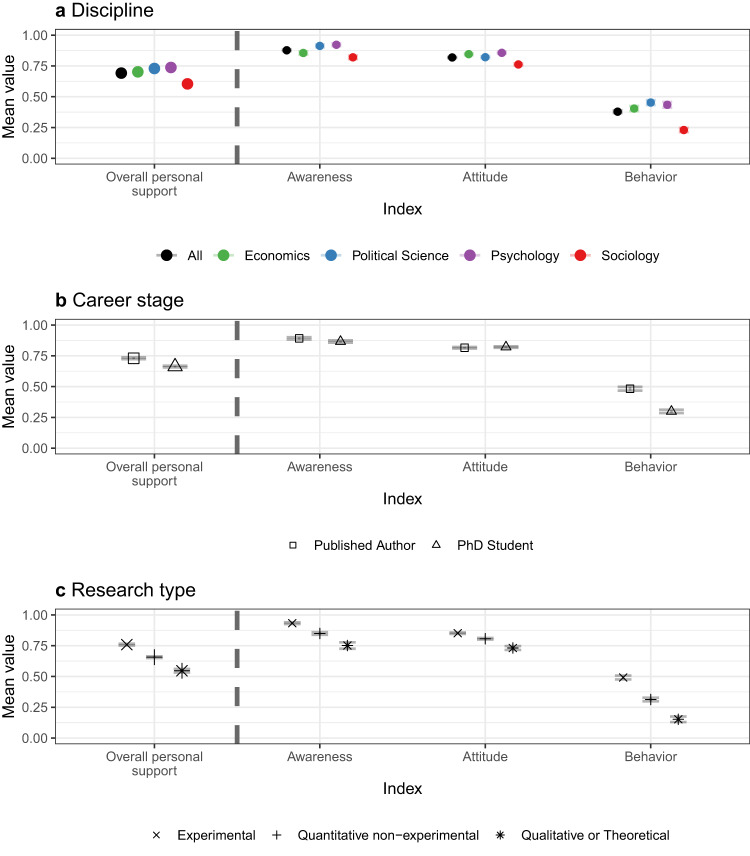
Open science awareness, attitudes and behavior—by research type. Dots represent means and lines around the dots are 95% confidence intervals for the estimates. Awareness is an index comprised of questions related to the respondent’s (i) Awareness of posting data or code online, (ii) Awareness of posting study instruments and (iii) Awareness of pre-registration. In wave 2, Awareness is automatically coded as 1 for those who completed the first wave. Behavior is an index comprised of questions related to the respondent’s (i) Behavior of posting data or code online, (ii) Behavior of posting study instruments and (iii) Behavior of pre-registration. Attitudes is an index comprised of questions related to the respondent’s (i) Attitudes of posting data or code online, (ii) Attitudes of posting study instruments and (iii) Attitudes of pre-registration. Overall Personal Support is an average of the three indices. The questions and methodology that are used to construct the indices can be found in Supplementary Table 7. Panel **a** shows the responses across disciplines. Panel **b** shows the responses across career stages. Panel **c** shows the responses across research types. See Supplementary Tables 23 and 24 for the *n* for each data point.

Researchers across disciplines who use experimental methods show the highest levels of support and practice, followed by researchers who use non-experimental quantitative methods. Although qualitative researchers show the least support and lifetime usage, their stated support is still at relatively high levels, as shown in Fig. 2.

**Fig. 3:**
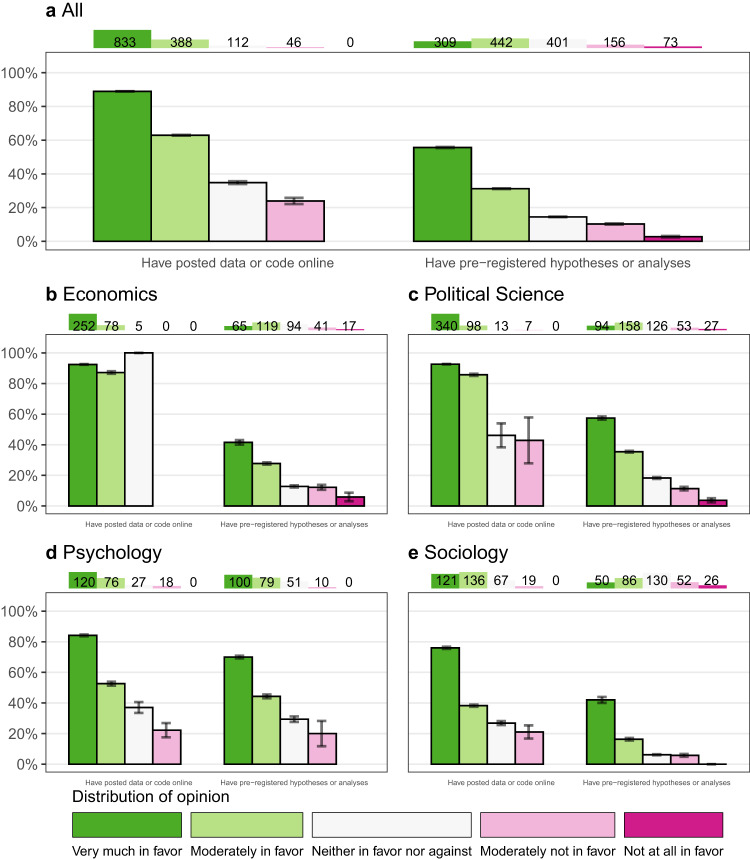
Posting behavior by attitude published authors. The chart shows the relationship between attitudes and behavior for the published authors in our sample. Each panel restricts to published authors in different disciplines. The chart shows attitudes and behavior for two open sciences practices: posting data or code online (left side of each panel) and pre-registering hypotheses or analyses (right side of each panel). Each bar represents published authors who stated that they were more or less supportive of the open science practice, with green indicating more support for the practice and red colors indicating less support for the practice. The height of the bar then displays the proportion of authors (i.e., the mean) with this attitude toward the open science practice who have done the practice previously. 95% Confidence Intervals are shown by the black whiskers on top of the bar. The number on top of the panel shows the total number of authors who have this attitude toward the practice. Panel **a** shows data from all published author responses, and Panels **b**–**e** show responses from published authors identified as economists, political scientists, psychologists, and sociologists, respectively.

**Fig. 4:**
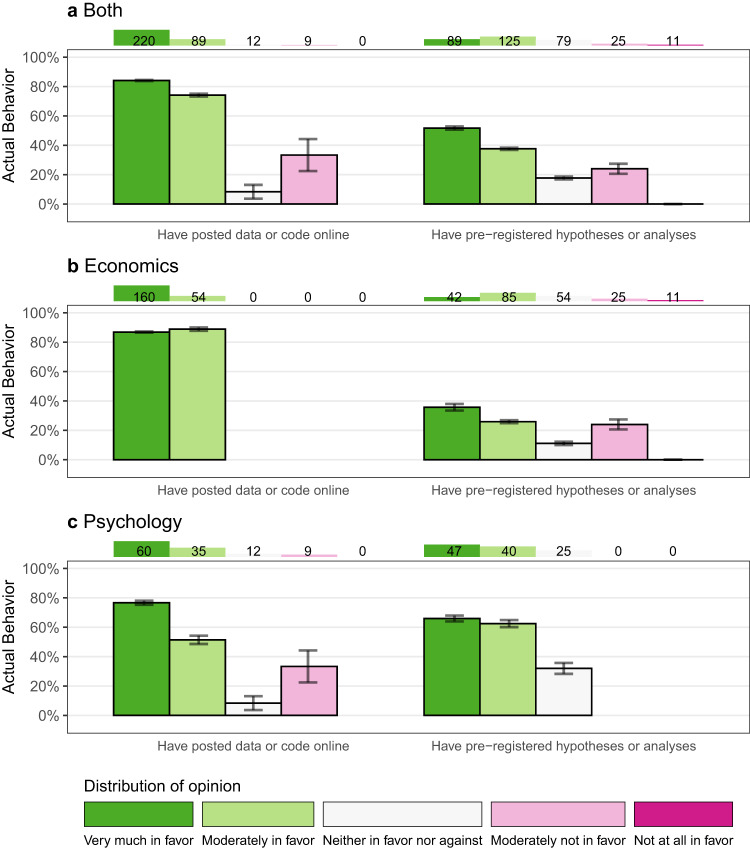
Posting behavior by attitude published authors, hand audit. The chart shows the relationship between attitudes and behavior for all the hand audit data (Panel **a**) and separately for the Economist (Panel **b**) and Psychologist (Panel **c**) published authors in our sample, who we validated the behavior of during our hand audit. The chart shows attitudes and behavior for two open sciences practices: posting data or code online (left side of the panel) and pre-registering hypotheses or analyses (right side of the panel). Each bar represents published authors who stated that they were more or less supportive of the open science practice, with green indicating more support for the practice and red colors indicating less support for the practice. The height of the bar then displays the proportion of authors (i.e., the mean) with this attitude toward the open science practice who have done the practice previously, as found in our audit. 95% Confidence Intervals are shown by the black whiskers on top of the bar. The number on top of the panel shows the total number of authors who have this attitude toward the practice.

In general, support for open science practices is predictive of lifetime usage. The correspondence between stated support for open science practices and their usage of the practices among the full sample is r = 0.32, *p* < 0.001, 95% CI = [0.29, 0.35] for posting data or code and r = 0.32, *p* < 0.001, 95% CI = [0.28, 0.35] for pre-registration (see Figs. 3 and 4 for additional details). When we just look at published authors, who have had more opportunity to put their attitudes into practice, the correlations rise to r = 0.44, *p* < 0.001, 95% CI = [0.40, 0.49] for posting data or code, and 0.36, *p* < 0.001, 95% CI = [0.32, 0.41] for pre-registration. These correlations are comparable to the correspondence between height and weight in the US (r = 0.44^20^), suggesting that open science attitudes are meaningful in that they correspond to social scientists’ methodological practices.

### Perceived norms

**Fig. 5:**
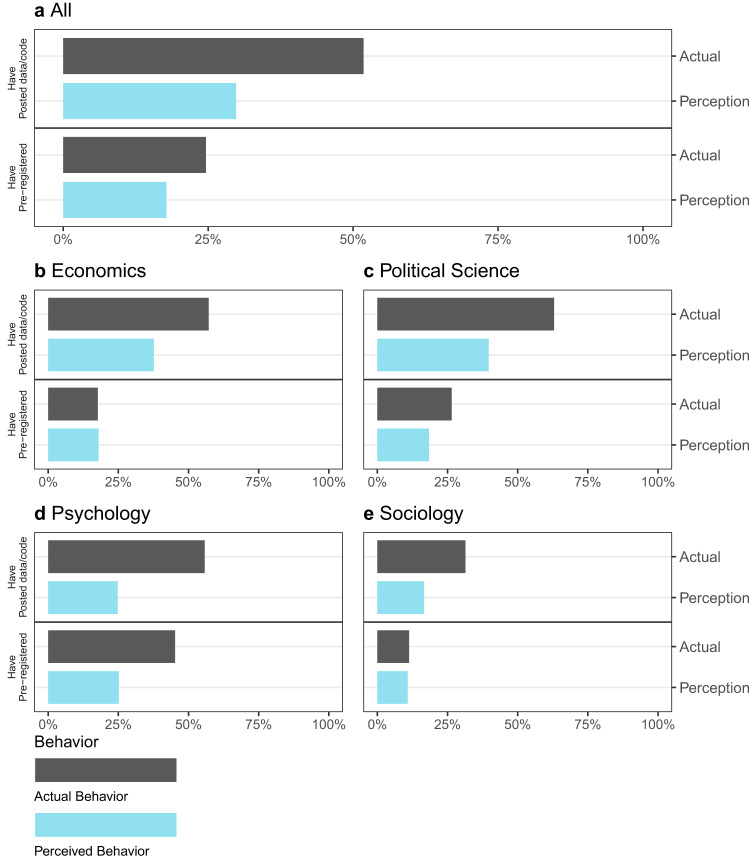
Actual and perceived behavior of open science practices. The chart shows differences between perceived and actual behaviors regarding two practices: having ever posted data or code online and having ever pre-registered hypotheses or analyses. The analogous data restricted to published authors or PhD students are presented in Supplementary Figs. 25 and 26, respectively. Within each panel, the top bar shows the fraction of scholars in a specified discipline that have ever engaged in an open science practice. The bar underneath shows the average perception among respondents about what proportion of scholars in their discipline have engaged in open science practice. Panel **a** shows data from all respondents, and Panels **b**–**e** show responses from researchers identified as economists, political scientists, psychologists, and sociologists, respectively.

We measured respondents’ perceptions of open science norms in their discipline, specifically, asking them to estimate what percentage of researchers in their discipline engage in (1) posting code and data online and (2) pre-registering hypotheses or analyses in advance of a study. Figure 5 depicts scholars’ perceptions of behavioral norms in their discipline against the actual lifetime rates of behavior as reported by survey respondents. Two findings are apparent. First, normative perception of behavior consistently underestimates actual lifetime rates of self-reported behavior. The pattern across all disciplines is particularly strong for posting data or code. We also observe an underestimation of perceived lifetime usage of pre-registration in psychology and political science, the two disciplines with the highest levels of actual pre-registration rates.

**Fig. 6:**
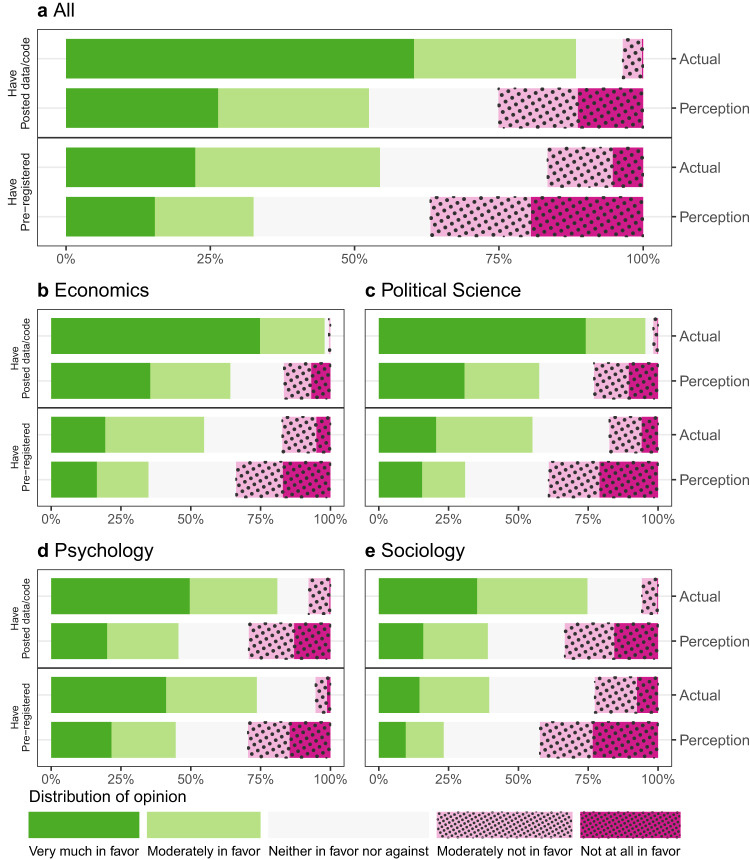
Perceived and actual opinions about open science by discipline—pushlished authors. The chart shows differences between perceived and actual support for two practices: posting data or code online and pre-registering hypotheses or analyses. The sample is restricted to published authors. Each panel represents the same published authors in a particular discipline. Within each panel, the first green and red bars show the distribution of support for Posting data or code, with green segments indicating more and red segments less support. The second bar shows the perceived distribution of support for the practice among published authors. This is constructed by asking individuals what percentage of researchers in their field they believe fall into each opinion category and then averaging over their responses. The third and fourth bars are analogous bars showing actual and perceived support for pre-registration. Panel **a** shows data from all published author responses, and Panels **b**–**e** show responses from published authors identified as economists, political scientists, psychologists, and sociologists, respectively.

We also measured perceived norms of support for open science practices. As shown in Fig. 6, we find the same pattern of underestimation in norm perception regarding perceived versus actual *support* for open science practices, particularly for attitudes toward pre-registration. These data show that despite the widespread usage of open science practices, the actual lifetime prevalence rates of behavior would be surprising to many of our survey respondents, who appear to significantly underestimate open science behavior and support.

These data suggest a lag in perceived norms behind actual rates of open science behavior in the social science disciplines. There are alternative interpretations of this pattern of data; we can largely rule out the possibility that high rates of lifetime usage are based on selection into our sample and on over-reporting actual rates of open science behavior (see description of our hand audit in Supplementary Information Section 1.4). By design, about half of our sample was drawn from authors who had published in their disciplines’ top journals. For these relatively well-published authors, their practices may not be representative of the entire discipline, whereas their perceptions of the field may take into account the mixture of scholars who do and do not publish in the same top journals. This aspect of our research design may exaggerate the lag found between perceptions of and actual lifetime rates of behavior and support in the field.

### Change over time

**Fig. 7:**
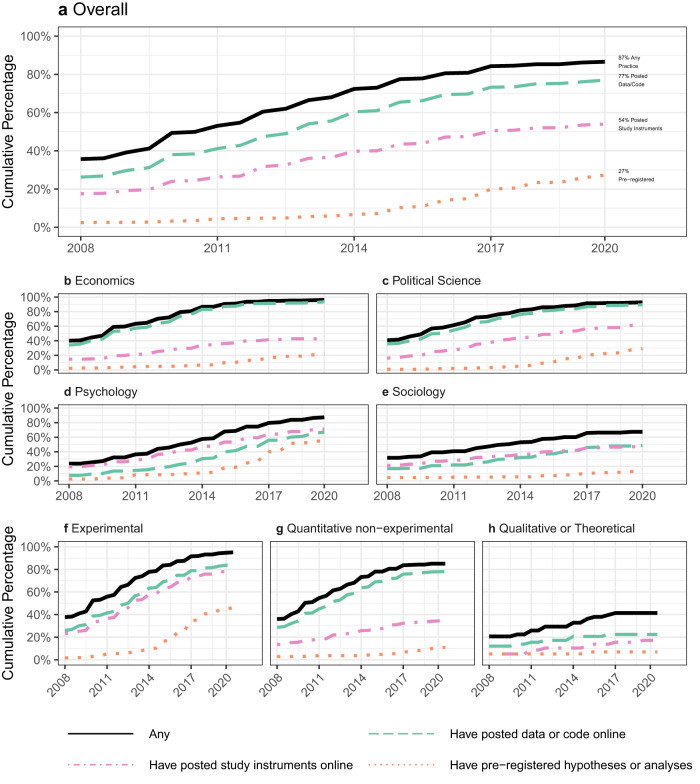
Year of first usage of open science practices. The chart shows the cumulative proportion of scholars (who earned their PhD in 2009 or earlier, *n* = 782) in a given year who adopted an open science practice in that year or previously. Data are taken from responses to the question form “Approximately when was the first time you [OPEN SCIENCE PRACTICE?]”. Panel **a** shows data from all scholar respondents, and Panels **b**–**e** show responses from scholars identified as economists, political scientists, psychologists, and sociologists, respectively. Panels **f**–**h** show responses from scholars who self-reported primarily being experimentalists, quantitative non-experimental, and qualitative or theoretical, respectively.

We next assess how the usage of open science practices has changed over time using survey respondents’ self-reported year of first adopting a practice. Figure 7 presents the cumulative proportion of scholars who have adopted open science practices over time. We focus on scholars who received their PhD by 2009, as they had the opportunity to engage in these practices over much of the last decade (see Supplementary Fig. 11 for robustness to different PhD cutoff dates). 87% of scholars reported having ever used an open science practice by 2020, nearly doubling from 49% in 2010. While the sharing of data, code, and study instruments has grown fairly consistently over the recall period, the use of pre-registration has increased since 2013. Posting data or code online is the most common practice, followed by posting study instruments online and then pre-registration. Those who reported adopting an open science practice at some point in the past are likely to have employed it in their most recent research project (see Supplementary Tables 29 and 30), indicating that usage tends to be persistent.

While there is an upward trend in all four disciplines, Panels b–e of Fig. 7 show that the reported year of adoption patterns differ across disciplines. The evolution of adoption in economics and political science appears relatively similar, with a rapid increase in the lifetime rates of posting data or code online. Psychology researchers were lagging behind economics and political science scholars until recently for all practices, but over the last few years, psychology has had the most rapid increase in adoption. Sociology has the lowest levels of lifetime usage for all open science practices, but as with the other fields, there has been a steady recent increase.

Reported adoption of all highlighted open science practices has been highest for researchers using experimental methods across social science disciplines (see Panels f–h of Fig. 7). Lifetime prevalence rates for all practices are the lowest among researchers using exclusively qualitative methods^21^, which likely helps to explain the lower rates in sociology, where such methods are more common. The high rates of adoption of pre-registration in psychology are due both to greater shares of psychologists engaging in experimental research and a higher adoption rate among experimental psychologists than experimental researchers in the other disciplines (see Supplementary Fig. 14).

## Discussion

Our large-scale survey of scholars in four social science disciplines—economics, political science, psychology, and sociology—indicates that there is widespread support for open science practices. Support for posting data or code is higher than support for pre-registration, although on average, support is greater than 50% even for pre-registration. We find little credible evidence of a difference between the stated support of newer entrants to fields and published authors. There is variation among the social science disciplines, with sociologists reporting less support than economists, political scientists, and psychologists. However, favorability is relatively high even among sociologists.

Additionally, there has been widespread lifetime usage of open science practices, meaning that a researcher has used a particular practice at least once. This is particularly the case among published authors, who have had more opportunities to try these practices. The lifetime prevalence of open science practices has increased over the past decade from 49 to 87%, according to social scientists’ recollections of when they first used an open science practice. Use is highest among economists and political scientists, where posting data or code is near-universal. Pre-registration is highest among psychologists, perhaps because of their experimental and original data-collection focus (sub-areas of economics that also focus on experimental data collection, such as development economics, feature high usage of pre-registration). We acknowledge these open science practices may, in some cases, be insufficient for replication or reproducibility—data and code shared may not always work in practice, and pre-analysis plans can vary widely in quality—so they should be viewed as a step in the direction of open science rather than necessarily corresponding to best practice^22^.

The hand audits of self-reported behavior concord with social scientists’ reports, suggesting that the self-reported data is generally accurate. However, social scientists’ perceptions of the behavior and attitudes within their fields are less than perfectly accurate. Social scientists appear to underestimate the amount of support and lifetime usage in their fields for open science practices (although the gap may be partly due to differences between the relatively elite sample we surveyed and members of these disciplines as a whole). For many reasons, this is not a surprise. Open science adoption is not always straightforward to recognize. Institutional practices aimed at making open science usage more visible, such as open data and pre-registration badges placed on some journals’ publications, might help more social scientists estimate accurately and shift the perceived norms of open science toward greater adoption.

A number of limitations should be considered when interpreting the results of this study. First, there are some limitations to our survey measures. The survey instrument asked about data and code sharing jointly, rather than including separate measures of each. While these two practices often go together to allow others to verify and reproduce results, it is possible that some researchers have different attitudes toward posting data vs. posting code or engaging in one of these practices but not the other. For pre-registration, we asked about attitudes toward pre-registering study hypotheses or analyses in advance of a study but did not separate out attitudes toward different types of pre-registration (e.g., pre-analysis plans that include fully drafted analysis code vs. shorter pre-registrations that focus more on hypotheses and research designs). Researchers may have different attitudes toward more vs. less detailed pre-registrations, and this likely varies by field. Additionally, there are a number of increasingly common open science practices that we did not ask about in this survey, such as pre-publication verification of results by co-authors or journals and alternative forms of results-blind peer review like registered reports. Finally, when we compare respondents’ own open science behavior to their perceptions of whether others in their field engage in the behaviors, our measurement doesn’t correspond perfectly. We compare whether participants have ever used a practice (lifetime prevalence) to their estimation of the percentage of others in their discipline who use the practice.

There are also some limitations to our sample and sampling strategy. Since our sample was focused on authors who publish in highly-cited journals and students at highly-ranked institutions, we are left with open questions regarding the generalizability of our results to the disciplines at large. This sample could particularly modify the interpretation of the underestimation of favorable attitudes toward and practice of open science. Additionally, although we used generous incentives and conducted two waves of data collection to achieve the highest possible response rate, our overall response rate is 53%. While we test and control for non-response bias (see section 4.4.3), it remains a possibility that unobservable characteristics are correlated with response, thus biasing the results.

There remain a number of important unresolved issues regarding the spread of open science in the social sciences that are beyond the scope of this article. A frequent topic of discussion is the extent to which the adoption of pre-registration and pre-analysis plans will spread beyond experimental studies into observational research^23,24^. Some of the ambivalence toward pre-registration we document in this study may be related to concerns that these approaches may not be well-suited to certain branches of non-experimental analysis. On the one hand are concerns that divisions exposed during ongoing open science debates will persist into the future and possibly lead to the fracturing of social science disciplines into rival camps, each espousing distinct methodologies. On the other hand, the high levels of latent support for open science that we document in this article hold open the possibility of an eventual convergence to new norms.

Perhaps the most important question that the present research was not designed to address is which new open science practices will generate positive impacts on the credibility and quality of our science and which will instead have limited benefits despite potentially large adoption costs. To date, there are only tentative answers to this question (e.g., see refs. ^16,17,25–27^), and it remains a key direction for future meta-science research.

## Methods

Our research complied with all relevant ethical regulations, including obtaining informed consent from each participant, and was approved by the Princeton Institutional Review Board (Protocol #11972). The survey was administered twice: once between April and August 2018 (Wave 1) and once between March and July 2020 (Wave 2). Results broken down by survey wave are included in the supplementary materials, as pre-specified. Data was collected using a custom interface on top of Qualtrics, and data was analyzed using R (4.0.2). We pre-registered analyses for the survey and posted the pre-analysis plan and study materials on the Open Science Framework on April 3, 2018 (see https://osf.io/zn8u2/registrations for pre-registration and Supplementary Information Section 1.1 for details, including deviations from the pre-registration in Supplementary Table 1).

### Sample

Within the practical and budget constraints of the project, we opted to focus on a sample of scholars who (in our view) were likely to be active and influential researchers. Our population consists of scholars at two career stages: published authors and PhD students. We randomly drew the published authors sample for the first wave of the survey from the complete set of authors who had published at least once within a range of 3 years (2014–2016) in 10 of the most-cited journals for each discipline. The selection of journals was based on citation impact factor. We also added the respective version of the Annual Review for each discipline. In total, we have 45 journals, shown in Supplementary Tables 2 through 5. For the PhD student sample, we drew from the complete set of PhD students enrolled in each discipline in the top-20 North American Universities according to the Times Higher Education Social Science ranking during the fall of 2017. The complete list of schools used can be seen in Supplementary Table 6. PhD students who are also published authors were sampled only as PhD students.

The universe from which we sampled consisted of over 22,000 authors and students. E-mail addresses for authors were gathered from journal articles and department webpages; student addresses were similarly taken from department webpages. We used power calculations to estimate that *N* = 3200 responses would be necessary to detect meaningful differences for published authors across disciplines; specifically, a sample size of 3200 allowed us to reliably (at 80% power and 5% significance threshold) detect positive effect sizes as small as Cohen’s d (standard deviation units) of approximately 0.2, and even smaller effects of 0.14 when pooling published authors and graduate students when using a two-tailed t-test. All tests performed in the main text and supplementary materials are two-tailed t-tests conducted under the assumption that the data are independent and identically distributed and errors are potentially heteroskedastic. Since we have up to two surveys for each individual sampled, the primary analysis presents people’s most recent responses to our surveys (results using their average response across the two rounds can be found in Supplementary Information Section 2).

### Participant incentives and response rates

Achieving a high response rate was a critical issue for the validity of our study. Several previous surveys on related transparency and reproducibility topics featured minimal or no monetary compensation for participants and had fairly low response rates, most in the range of 10 to 24% (see refs. ^13,28^). We sought to generate longitudinal data on a far more representative population of active social science researchers by offering much higher levels of compensation.

In the first wave, participants were randomly offered either a standard or high incentive. The levels differed between published authors and PhD students and were based on the response rates from a pilot study we conducted. Published authors were compensated either *$*75 or *$*100, and graduate students either *$*25 or *$*40. Initial contact with selected respondents was made via email. There were three reminders at intervals following the initial email contact. PhD students offered the High incentive had an 8.2 percentage point higher response rate, and published authors offered the High incentive had a 0.8 percentage point higher response rate. Due to the limited effect of “High” incentives, all participants were offered the “Standard” incentive in wave 2.

Overall, 6231 individuals were invited in wave 1 and again in wave 2, of whom 6114 were actually contacted (emails did not bounce). Forty-two percent of this sample identified as women. We did not consider gender in the study design because we did not have evidence that this would be a major predictor of involvement in open science practices, nor was this a leading hypothesis among the research team. A total of 3257 responded in at least one wave, giving us an overall response rate of of 53%. A total of 114 individuals explicitly opted out, and 59 partially completed the survey (using only the most complete response from an individual across both waves).

Supplementary Fig. 2 presents the overall response rate by career stage (PhD student or published author) and field, which ranged from 47% in Psychology to 60% in Political Science. PhD students responded at or above 50% in every field, while published authors (who had predominantly completed their doctoral training) responded at somewhat lower rates. The response rate for published authors from psychology journals is somewhat lower than that for the other disciplines’ journals. This may be due to the fact that a subset of psychologists often publish with scholars or clinicians from other fields who are less active empirical researchers and therefore may be less likely to respond to an invitation to complete a survey focused on research methods. Consistent with this explanation, the response rate from authors who published in clinical and neuroscience-focused journals is considerably lower than the rate for social and developmental psychology journals (see Supplementary Fig. 6 for response rates by journal). Similarly, the response rate for authors who had published in macroeconomics journals is somewhat lower than the rate from other economics journals, possibly due to the greater share of articles based on theoretical or simulation approaches rather than quantitative empirical data analysis, in those journals.

### Measures and indices

We focus on two open science practices in the paper: posting data or code online and pre-registering analyses. For these practices, we ask about awareness, attitudes, behavior, descriptive norms, and prescriptive norms. We also ask about awareness, attitudes, and behavior for the practice of posting study instruments online, but we did not ask about norms for this practice due to space limitations in the survey. Our indices of awareness, attitudes, behavior, and overall personal support include this practice. More details on how we defined these practices in the survey can be found in Supplementary Information Section 1.2.2.

#### Lifetime prevalence

Many of the analyses focus on lifetime prevalence, which is a measure of whether participants have ever engaged in an open science practice. For publicly posting data and code, participants were asked: “Approximately how many times have you [publicly posted data or code online]?” The measure of lifetime prevalence is coded as 1 if the participant says that they have posted data or code one or more times, and as 0 if they have never done it. The two other practices follow the same format.

#### Attitudes

For the analyses comparing people’s own self-reported behavior toward open science practices with their attitudes, participants were asked: “What is your opinion of publicly posting data or code online?” and “What is your opinion of pre-registering hypotheses or analyses?” Response options ranged from 1 = Not at all in favor to 5 = Very much in favor.

#### Norms

For the analyses comparing people’s own self-reported behavior toward open science practices with their perceptions of behavior in their field (i.e., descriptive norms), participants were asked: “In your estimation, what percentage of researchers across the discipline of [Discipline] publicly post data or code online?” and “In your estimation, what percentage of researchers across the discipline of [Discipline] pre-register hypotheses or analyses in advance of a study?” Response options ranged from 1 = Not at all in favor to 5 = Very much in favor.

For the analyses comparing people’s own attitudes toward open science practices with their perceptions of attitudes in their field (i.e., prescriptive norms), participants were asked: “In your estimation, what is the distribution of opinion across the discipline of [Discipline] about publicly posting data or code online?” and “In your estimation, what is the distribution of opinion across the discipline of [Discipline] about pre-registering hypotheses or analyses in advance of a study?” Participants used a dynamic histogram programmed in Qualtrics to indicate the percentage of people in their field who fell into five opinion categories ranging from 1 = Not at all in favor to 5 = Very much in favor (see Supplementary Fig. 1 for an example of the dynamic histogram).

#### Indices

To assess levels of open science awareness, attitudes, and behavior across fields, career stage, and methodological orientation, we aggregate individual survey questions for each of the three open science practices (posting data or code online, posting study instruments, and pre-registration) into three separate measures of awareness (1 item; e.g., “Have you ever heard of the practice of pre-registering hypotheses or analyses in advance of a study?”, behavior (3 items; e.g., “Approximately how many times have you pre-registered hypotheses or analyses in advance of a study?”, and attitudes (2 items; e.g., “To what extent do you believe that pre-registering hypotheses or analyses is important for progress in [Discipline]?” The full set of measures and questions can be found in Supplementary Tables 7 and 8. We then create awareness, attitudes, and behavior indices that aggregate across the three open science practices by taking simple averages and a broad “Personal support for open science” index that combines the awareness, attitudes, and behavior indices into one by taking the simple average of the three sub-indices. Details of the aggregation method are described in Supplementary Table 8.

### Assessing reliability and representativeness of survey responses

The goal of this paper is to obtain an estimate of average attitudes, norms and perceptions for PhD students and published authors from top disciplinary journals. There are two major threats to this goal. First, one might worry that survey responses do not accurately reflect behavior and beliefs due to, for instance, demand effects. Second, one might worry that there is selection in our survey on a dimension that correlates with beliefs and behavior.

To assess the representativeness of those who responded to the survey of our selected sample, as well as the reliability of our survey results, we used data collected in manual and automated audits of open science behavior described below. These audits allowed us to collect data on objective measures of open science behavior for a random sample of published author respondents and non-respondents from economics and psychology, such as whether the respondent had previously posted data or code online. The goals of these audits were threefold. First, by comparing manual audit data to self-reports, we can assess the accuracy of self-reported data. Second, using both manual audits and web-based scraping audits allowed us to assess the degree to which web-based scraping approaches might be able to determine broad trends in open science. Finally, by auditing both survey respondents and non-respondents, we are able to assess the degree to which there is differential selection along observable dimensions in our sample.

#### Audit activities

In order to validate our survey responses and check for balance across respondents and non-respondents, we conducted an audit of our economics and some psychology published authors. Specifically, we randomly sampled (1) all economics published authors who completed our survey in both waves, (2) 150 economics published authors who were contacted but did not complete our survey in either wave and (3) 167 psychology published authors who were contacted in both waves, 119 of whom completed either wave of the survey. We chose to audit Economics and Psychology rather than all four fields due to project budget and staffing constraints. We expected that providing information on these fields would still be informative of the reliability of survey data for our entire sample.

We then conducted an audit, which was not pre-registered but we considered important in order for us to reliably interpret the survey data. For all sampled individuals, we conducted a hand audit of these authors’ pre-registration and data-posting behaviors using publicly available information. The first audit activity was completed between March 15, 2019, and March 29, 2019, for wave 1. The auditors searched through a broad set of websites and searched by last name only. They looked through the search results and tried to identify the published author using their first name or affiliation. Then, following the link associated with an identified author, auditors look for a (1) pre-analysis plan or (2) posted data or code on the websites. As soon as a match was found, auditors stopped searching and recorded the match and a link to the matched page. If no match could be found, the auditors recorded that no match was found. See Supplementary Information Section 1.4.1 for a full list of websites we searched and more detailed information on the protocol. After the second wave, auditors also searched for the 4–5 most recent publications by authors and checked journal websites for evidence of posting data or code directly. This was completed between June and November 2020.

Note that this audit process is in contrast to the procedure outlined by refs. ^18,19^, who follow a more tightly defined search process that focuses on mentions of data and pre-registration (as well as other open science practices) within journal articles themselves. While both audit methods have benefits, we found that our more wide-ranging audit activity seemed to uncover a larger number of open science practices that may have been missed in more narrowly defined audit protocols.

We also conducted automated audits to complement the hand audits. For automated audits, searches were made for items posted under a given name and email address, if available. The repositories scraped include the American Economic Association’s Randomized Control Trial Registry, the Evidence in Governance and Politics (EGAP) Registry, Dataverse, AsPredicted, and the Open Science Framework (OSF). The automated audit was conducted in August 2020, in line with database terms of service, and considered items posted before March 1, 2020. This second audit serves two important purposes. First, the automated audit allows us to validate the responses of a wider set of authors as it is much less time and effort-intensive than the manual audit, although necessarily with a more limited scope. Second, we can compare the results from the automated audit to those from the manual audit and self-reported data to understand which of the methodologies is best at capturing the extent of open science behavior in these fields.

#### Reliability of survey responses

The manual audit yields two main results regarding the reliability of our survey data. First, there is a high rate of agreement between self-reports and actual behavior: despite only checking a limited number of online sources, we were able to validate more than 91% of economists’ responses regarding the adoption of open science practices, meaning in 91% of cases our audit confirms the respondent’s response (see Supplementary Table 11). While the corresponding number is slightly lower for psychologists, we still validate approximately 75–80% of psychologists’ responses (see Supplementary Tables 13 and 14). More importantly, among psychologists, we find no evidence for one-sided reporting errors, suggesting that this reflects noise rather than demand effects. The fact that we validate such a high proportion of survey responses suggests that our responses are accurate reflections of the state of these disciplines and are not driven by a demand effect.

Second, comparing the results of the survey to the manual and electronic audit, we find a higher rate of agreement between the survey results and the manual audit than we do the electronic audit. While all three methods generally agree on measures of pre-registration, the electronic audit misses 70% of the data and code that scholars post. This arises because of the large set of websites that authors now use to post data or code, at least in economics. Notably, this means that methods of gauging open science behavior that rely on scraped data from major repositories may significantly understate the extent of open science behavior. Until more centralized repositories for data or code exist, as they do for pre-registrations, surveys can play an important role in helping to capture the extent of open science behavior across these disciplines.

#### Representativeness of survey responses

While the preceding section shows that the survey data represent accurate reflections of behavior for the subset of respondents that responded to the survey, one might worry about selection into the survey sample. Selection may not be an issue if individuals are essentially “missing at random,” whereas if selection into the sample is correlated with our outcomes of interest, this becomes problematic for inference about the population parameters. Finally, even in the latter case, one might be able to adjust estimates of population parameters for this selection if the selection is largely driven by observable characteristics rather than outcomes.

To assess the degree of selection into the survey sample, we use the hand audit of respondent and non-respondent economist published authors. We regress measures of audited behavior (posting data/code, pre-registering, or either) on an indicator for whether an individual completed the survey. In additional specifications, we also control for observable features that were collected for respondents and non-respondents, including disciplinary subfield, publication track record, job type and institution.

Unadjusted for observables, we see limited credible evidence for selection on data-posting into the survey sample, while evidence for selection on pre-registration: while respondents are not meaningfully more likely to have posted data, they are more likely to have pre-registered previously (see columns 1, 4 and 7 of Supplementary Table 15).

We then control for observable characteristics. Disciplinary subfield is an especially important predictor of differences in these estimates. In particular, it appears that economists in fields with a more empirical orientation (e.g., development economics, behavioral economics) are more likely to respond to the survey, particularly as compared to theory-focused scholars (see column 10 of Supplementary Table 15). Controlling for these compositional differences, we see very limited credible evidence for differences in behavior between respondents and non-respondents: controlling for these fields and other easily observable characteristics eliminates 75–80% of the difference in behavior between respondents and non-respondents, with the remaining differential not being statistically significant at conventional levels (see columns 3, 6 and 9 of Supplementary Table 15).

To model selection, we predict the propensity of response based on covariates. We also assess the robustness of this approach by using multiple models of the response probability. See Supplementary Information Section 1.5 for more details. Generally, results adjusting for selection under various propensity score models are very similar to the unadjusted results. See Supplementary Tables 18–21 for the full set of adjusted results.

We also provide some evidence that selection into our sample is on covariates rather than outcomes by again drawing on our hand audit of economists for evidence that this assumption may be correct. In particular, Supplementary Table 15 shows that among economists, there is an apparent selection on behavior (particularly pre-registration) into our sample. However, once we adjust for author’s subfields, we find little credible evidence that respondents and non-respondents differ greatly in behavior.

Another finding in this paper is that individuals underestimate how favorable other scholars are toward open science. Favorability, unfortunately, is not a measure that is easily auditable. Therefore, there may be several interpretations of this finding. Those who chose to respond to our survey invitation may be more supportive of open science than non-respondents, further shifting sample means. However, the evidence from the audit activity described above suggests the degree of selection is unlikely to be large: the patterns presented in Figs. 5 and 6 are robust to multiple approaches to accounting for non-random selection into the survey sample (see Supplementary Information Section 1.5). Another explanation is that respondents are over-reporting their usage of and support for open science for reasons of self or social image. However, the web hand audit shows that self-reported open science practices are accurate. Additionally, admitting some social desirability toward responding favorably about open science in an anonymous survey seems to support the idea that a relatively strong social norm in favor of open science has already developed.

### Reporting summary

Further information on research design is available in the Nature Portfolio Reporting Summary linked to this article.

### Supplementary information

Supplementary Information

Reporting Summary

## Data Availability

The de-identified data generated in this study have been deposited in the Open Science Framework at https://osf.io/zn8u2/ and can be freely accessed. The raw respondent-provided subfield data are protected and are not available to protect anonymity. See Supplementary Information Section 1.1.1 for links to all posted materials.

## Code availability

The de-identified code used to analyze the data from this study has been deposited in the Open Science Framework at https://osf.io/zn8u2/ and can be freely accessed. Code used includes R (4.0.2), tidyverse (2.0.0), knitr (1.43), ggtext (0.1.2), patchwork (1.2.2), SuperLearner (2.0-26), cvAUC (1.1.0), cobalt (4.3.0), glmnet (4.1-1), CBPS: Covariate Balancing Propensity Score (0.21), and arm (1.11-2). See Supplementary Information Section 1.1.1 for links to all posted materials.
