# Supplementary material for: Survey of open science practices and attitudes in the social sciences
Source: Nat Commun. 2023 Sep 5;14:5401. doi: 10.1038/s41467-023-41111-1 (PMC10480148; doi:10.1038/s41467-023-41111-1)
Supplement: Supplementary file 1 — Supplementary Information [file 41467_2023_41111_MOESM1_ESM.pdf]

Supplementary Information for Survey of Open Science Practices and Attitudes in the Social Sciences

Joel Ferguson[1†] Rebecca Littman[2†] Garret Christensen[3†]
Elizabeth Levy Paluck[4*] Nicholas Swanson[5†]
Zenan Wang[5†] Edward Miguel[5*]
David Birke[5†] John-Henry Pezzuto[6†]

[1]University of California, Berkeley, Department of Agricultural and Resource Economics, Berkeley, California, USA
[2]University of Illinois Chicago, Department of Psychology, Chicago, Illinois, USA
[3]Federal Deposit Insurance Corporation, Washington, District of Columbia, USA
[4]Princeton University, Department of Psychology, Princeton, New Jersey, USA
[5]University of California, Berkeley, Department of Economics, Berkeley, California, USA
[6]University of California, San Diego, Rady School of Management, La Jolla, California, USA

† These authors contributed equally.
*These authors jointly supervised this work. To whom correspondence should be addressed:
E-mail: emiguel@berkeley.edu, epaluck@princeton.edu.

August 2023

Supplementary Information

1. Supplementary Methods

   1.1. Supplementary Posted Materials

      1.1.1. Links to Registration, Data and Code

      1.1.2. Notes on project registration

      1.1.3. Notes on the pre-analysis plan

      1.1.4. Notes on the pre-analysis plan amendment

      1.1.5. Notes on the project data and code

      1.1.6. Pre-Registration Deviations

   1.2. Supplementary Information on Survey and Data

      1.2.1. Sample

      1.2.2. Open Research Practice Definitions

      1.2.3. Measures and Indices

   1.3. Response Rates

   1.4. Supplemetary Audit and Online Data

      1.4.1. Audit Protocol - Open Science behaviors

      1.4.2. Audit Protocol - Author Subfield

   1.5. Supplemetary material on Reliability and Representativeness of Survey Responses

1.5.1. Reliability

1.5.2. Representativeness

2. Supplementary Results

2.1. Adoption

2.2. Attitudes, Behavior and Norms

2.3. Broad Index Heterogeneity Results

SI 1 Supplementary Methods

SI 1.1 Supplementary Posted Materials

SI 1.1.1 Links to Registrations, Data and Code

This project's OSF page

This project's original OSF registration

The survey conducted, uploaded to OSF

Quick link to the Wave 1 pre-analysis plan on OSF

Quick link to the Wave 2 pre-analysis plan on OSF

Quick link to the amended pre-analysis plan for Wave 2 on OSF

SI 1.1.2 Notes on the project registration and pre-analysis plan for survey wave 1

This project was conceived of as a longitudinal study of scholars' and PhD students' attitudes and behavior towards open science practices: this was stated in the pre-registration for the first wave of surveys for this project. In the study registration linked above, the summary states "In particular, the overall research project will also include a follow-up survey with questions largely identical to those featured in this first wave of data collection, as well as information on participants' open science-related behaviors pulled from public sources. For this portion of the project, we intend to submit a future pre-analysis plan after seeing results from our first survey wave.

We posted our pre-analysis plan for the first wave of data collection on April 3, 2018. This can be found by navigating to the project's OSF page, clicking Registration, then clicking "View"→"Files"→"Archive of GitHub: garretchristensen-BITSS3S"→"src"→"pre_analysis"→"pre_analysis_plan.html". Readers must save the file and open in a web browser to view the file. In 2020 we uploaded an identical version

of this file to the front page of the OSF project to provide easier access. This pre-analysis plan described two distinct sets of analyses. The first analysis was a set of descriptive analyses that we include in this paper. In addition, the pre-analysis plan also included a set of experimental interventions that we included as part of our survey. The results of these interventions are not included in this paper as we plan to include them in a later, separate publication.

SI 1.1.3 Notes on the pre-analysis plan for survey wave 2

The pre-analysis plan described in the previous section (henceforth PAP1) was written for a single wave of surveys of published authors and PhD students across 4 social science disciplines that was completed between April and August 2018 (Wave 1). PAP1 was written with the intention of writing a paper that would use only the data from this single wave of surveys. A short conference proceedings paper was published using only the economics subset of this data in May 2020 [1], and a working paper using all disciplines, following the lines of PAP1 was released[1]. As briefly mentioned in PAP1, we had planned from the very beginning to collect additional rounds of data; we launched this second wave of data collection in May 2020.

Prior to the launch of the second wave of data collection in March 2020, we posted a second pre-analysis plan specifically for that wave of data collection (henceforth PAP2). PAP2 focused primarily on analyzing experimental outcomes related to the two survey waves, which as mentioned above are intended for inclusion in a separate paper. In addition to the experimental analysis, PAP2 describes performing a descriptive analysis analogous to that described in PAP1.

[1]Available at https://osf.io/preprints/metaarxiv/5rksu/

SI 1.1.4 Notes on the pre-analysis plan amendment

Given that no cross-disciplinary analysis from the first survey wave was published, we decided to amend PAP1 to incorporate the additional data that we collected from Wave 2 and to consolidate and expand on the descriptive analyses in PAP1 and PAP2. We did this for the following reasons. First, we believed that incorporating the data from Wave 2 might allow us to speak more to recent changes in the perceptions and adoption of open science practices. Second, using the data from Wave 2 allowed us to incorporate the responses of individuals that did not respond in Wave 1, making the combined sample more representative of the opinions of social science researchers originally sampled compared to the Wave 1 sample alone.

Therefore, the purpose of the pre-registration amendment was simply to modify (and expand) the data that we used for the paper relative to PAP1, rather than change the way that the statistical analysis will be performed.

Importantly, we want to state that at the time of uploading the pre-registration amendment we had not looked at any of the data from Wave 2, either individually or summarised in statistics, except for tracking the response rate. We acknowledge that for this claim to be verifiable, we should have technically registered (i.e. created a frozen, time-stamped version on the OSF.) Importantly, as shown in the paper, the core results of the paper do not change if the sample is restricted to only the first wave survey respondents.

SI 1.1.5 Notes on the project data and code

Data and code from the project is posted on the Open Science Framework. The main project page is at https://osf.io/zn8u2/; the direct link to the data and code is https://osf.io/hbpe8/.

SI 1.1.6 Pre-Registration Deviations

This table describes deviations from the pre-analysis plans listed above for each display item (figure or table) in the main text and supplementary materials. We consider both whether or not the concept of the analysis was included in a pre-analysis plan as well as how the analysis was implemented. For example, a description of the analysis represented in Supplementary Fig. 2 was not described in any pre-analysis plan, so it is not considered pre-registered in the table below. Supplementary Fig. 11, on the other hand, corresponds to the description of our pre-registered analysis of Evolution of Open Science Practices Over Time (Pre-Analysis Plan for Survey Wave 1, section 4.4.1), so it is considered pre-registered, but does not use the pre-registered cutoff dates, which is recorded in the Deviations column.

Supplementary Table 1: Deviations from Pre-Analysis Plans

Display Item	Pre-Registered?	Deviations
Figure 1	Yes	Discipline heterogeneity
Figure 2	Yes	Research type heterogeneity
Figure 4	No	
Figure 4	No	
Figure 5	No	
Figure 6	Yes	Discipline heterogeneity
Figure 7	Yes	Discipline and methodology heterogeneity
Supplementary Figures 2-4	Yes	Discipline heterogeneity
Supplementary Figure 5	No	
Supplementary Figure 6	No	
Supplementary Figure 7	No	
Supplementary Tables 9-14	No	
Supplementary Tables 15-21<br>Supplementary Tables 22-25	No<br>Yes	 Numbers rather than rates
Supplementary Figure 11	Yes	Students rather than<br>Published Authors who completed<br>their PhD before 2010
Supplementary Figures 9-12	Yes	None
Supplementary Figure 13	Yes	Discipline heterogeneity, key date flags
Supplementary Figure 14	Yes	Discipline×method heterogeneity
Supplementary Tables 26-28	Yes	Outcome is binary adoption
Supplementary Tables 29 and 30	No	
Supplementary Figures 15-22	Yes	None
Supplementary Figures 23-31	Yes	Discipline and career stage heterogeneity
Supplementary Tables 31-36	Yes	None

SI 1.2 Supplementary Information on Survey and Data

SI 1.2.1 Sample

Our population consists of scholars at two career stages: published authors and PhD students. We invited 6,220 individuals to participate in the first wave. All authors and PhD students who were invited to the first wave and were not screened from the survey (because, e.g., an author reported that they did not take part in empirical research) were also invited to the second wave, along with a supplementary sample of 400 individuals drawn from the same sampling frame.

<u>Published Authors:</u>

These are active social science researchers who have published in a top-10 leading journal within their discipline. We use the following definitions:

- Active: At least one publication in 2014-2016.
- Top-10 leading journals: The selection of journals was based on citation impact factor. We also added the respective version of the Annual Review for each discipline. In total we have 45 journals, shown in Supplementary Tables 2 through 5.
- Discipline: Before a participant entered the survey, we took an initial guess of their discipline. For PhD students it was their department, for published authors the discipline they have published in most frequently during 2010-2016, with ties split by the most recent publication. We used the initial guess to draw our sample, and for the analysis. The exception was the following, which occurred in a small number of cases: at the beginning of the survey we ask each participant for their primary discipline. If their answer did not match with the initial guess, and they indicated that they do not feel familiar enough to comment on the initially guessed discipline,

we asked them to choose which of the four disciplines they feel sufficiently familiar with. We assigned this discipline to them for our analysis. If they did not feel familiar enough with any of our four disciplines, the survey ended, and they did not become part of our analysis sample. Such respondents to the first wave of the survey were also not invited to participate in the second wave.

PhD Students:

These are current PhD students from top-20 North American doctoral programs within each discipline. We use the following definitions:

- Current: Listed on departmental websites in Fall 2017.
- Top-20 North American Universities: The 20 US and Canadian universities with the highest rank according to the Times Higher Education World University Social Science Rankings 2017. All PhD students were drawn from a single list of 20 universities. The complete list of schools used can be seen in Supplementary Table 6.

PhD students who are also published authors were sampled only as PhD students.

Supplementary Table 2: Economics Journals

Index	Journal	Publisher
NR	Annual Review of Economics	Annual Reviews
1	The Quarterly Journal of Economics	Oxford University Press
2	Journal of Political Economy	University of Chicago Press, JSTOR
3	American Economic Review	American Economic Association, JSTOR
4	Econometrica	Wiley, JSTOR
5	Journal of Economic Growth	Springer, JSTOR
6	Review of Economic Studies	Oxford University Press
7	Journal of Monetary Economics	Elsevier
8	Journal of Econometrics	Elsevier
9	Journal of Labor Economics	University of Chicago Press
10	The Review of Economics and Statistics	MIT Press

Sampling Frame Economics Published Authors Journals used to sample economics published authors. While the Annual Review of Economics is not ranked, it is included as it is an influential journal for the field. The selection of journals is based on citation impact factor. NR = “Not Ranked.”

Supplementary Table 3: Political Science Journals

Index	Journal	Publisher
NR	Annual Review of Political Science	Annual Reviews
1	American Journal of Political Science	Wiley
2	American Political Science Review	Cambridge University Press
3	The Journal of Politics	University of Chicago Press
4	British Journal of Political Science	Cambridge University Press
5	Political Analysis	Oxford University Press
6	Comparative Political Studies	SAGE Publishing
7	World Politics	Cambridge University Press
8	Political Behavior	Springer
9	International Organization	Cambridge University Press
10	International Studies Quarterly	Wiley

Sampling Frame Political Science Published Authors Journals used to sample political science published authors. While the Annual Review of Political Science is not ranked, it is included as it is an influential journal for the field. The selection of journals is based on citation impact factor. NR = "Not Ranked."

Supplementary Table 4: Psychology Journals

Index	Journal	Publisher
NR	Annual Review of Psychology	Annual Reviews
1	Psychological Science	SAGE Publishing
2	Psychological Bulletin	American Psychological Association
3	American Psychologist	American Psychological Association
4	Journal of Experimental Psychology - General	American Psychological Association
5	Trends in Cognitive Sciences	Elsevier
6	Social Cognitive and Affective Neuroscience	Oxford University Press
7	Journal of Personality and Social Psychology	American Psychological Association
8	Journal of Consulting and Clinical Psychology	American Psychological Association
9	Child Development	Wiley
10	Developmental Psychology	American Psychological Association

Sampling Frame Psychology Published Authors Journals used to sample psychology published authors. While the Annual Review of Psychology is not ranked, it is included as it is an influential journal for the field. The selection of journals is based on citation impact factor. NR = "Not Ranked."

Supplementary Table 5: Sociology Journals

Index	Journal	Publisher
NR	Annual Review of Sociology	Annual Reviews
1	American Sociological Review	SAGE Publishing
2	American Journal of Sociology	University of Chicago Press
3	European Sociological Review	Oxford University Press
4	Social Forces	Oxford University Press
5	Social Problems	Oxford University Press
6	Demography	Springer
7	Criminology	Wiley
8	Gender & Society	SAGE Publishing
9	Administrative Science Quarterly	SAGE Publishing
10	Sociology of Education	SAGE Publishing
11	Social Networks	Elsevier

Sampling Frame Sociology Published Authors Journals used to sample sociology published authors. While the Annual Review of Sociology is not ranked, it is included as it is an influential journal for the field. The selection of journals is based on citation impact factor and disciplinary expert recommendation. NR = "Not Ranked."

Supplementary Table 6: Top 20 North American Doctoral Programs

**Rank**	**University**	**Country**
1	Stanford University	US
2	Yale University	US
3	University of Chicago	US
4	Harvard University	US
5	Massachusetts Institute of Technology	US
6	University of Michigan-Ann Arbor	US
7	Princeton University	US
8	University of California, Los Angeles	US
9	University of California, Berkeley	US
10	Columbia University	US
11	University of Pennsylvania	US
12	Cornell University	US
13	Duke University	US
14	University of Wisconsin-Madison	US
15	University of Toronto	Canada
16	University of British Columbia	Canada
17	New York University	US
18	Northwestern University	US
19	University of Washington-Seattle	US
20	University of California, San Diego	US

Sampling Frame PhD Students PhD students in the paper were sampled from universities listed in the table. The ranking is the Times Higher Education 2017 Social Science ranking.

SI 1.2.2 Open Research Practice Definitions

In this section we reproduce the text defining open research practices used in the survey. This text is shown at the beginning of a module of questions focusing on the particular practice.

Posting Data or Code

The next several questions have to do with publicly posting data or code online for a completed study.

- By data we mean anonymized data, which could be qualitative or quantitative. Posted data might be raw, meaning in the form it was collected, or cleaned, meaning corrected for errors, transformed into scales or into coded themes, etc.
- By code, we mean the statistical coding files used to analyze or clean the quantitative data, or in the case of qualitative data, transform the data into themes, etc.

Please note that these questions are only about posting data or code, not about posting other study materials.

Study Instruments

The next several questions have to do with publicly posting study instruments online for a completed study.

- By study instruments, also known to some researchers as study protocols, we mean survey questions, participant instructions, experimental intervention materials, and other documents used to gather the data.

In the next questions, when we use the term study instruments, please understand this term as referring to any and all of the items mentioned above.

Pre-Registering Hypotheses or Analyses

The next several questions have to do with pre-registering hypotheses or analyses for a prospective data collection or data analysis.

- By pre-registration, which some researchers call a pre-analysis plan, we mean writing down study predictions or planned analyses in advance of analyzing your outcome data, and posting the document on a site where it will be time-stamped.
- This may also include writing down predictions or planned analyses for a previously collected dataset in advance of examining the data.

SI 1.2.3 Measures and Indices

We aggregate individual survey questions into five measures (awareness, behavior, attitudes, descriptive norms, and prescriptive norms) for each of the three practices (posting data or code online, posting study instruments, and pre-registration). Details of the aggregation method are described in Supplementary Table 8.

We also measure trustworthiness of the literature, behavioral intentions, and projected norms through a set of questions.

We then aggregate the large number of measures to a smaller number of sub-indices and broad indices. Each sub-index is a simple average of measures, and each broad-index is a simple average of sub-indices. See Supplementary Table 7 and Supplementary Table 8 for details.

Altogether, our outcome variables for the descriptive analysis are:

- Sub-Indices: Awareness, Behavior, Attitudes, Descriptive Norms, Prescriptive Norms, Posting data or code online, Posting study instruments, Pre-registration

- Broad Indices: Personal support for open science, Norms, Overall open science, Trustworthiness of literature

The mappings from questions to sub-indices, and from sub-indices to broad-indices can be found in Supplementary Tables 7 through 8.

Supplementary Table 7: Mapping Measures to Indices

Measure	Sub-Index	Broad Index
1.1.1 Awareness of posting data or code online	1.1 Awareness	1. Personal support for open science
1.1.2 Awareness of posting study instruments		
1.1.3 Awareness of pre-registration		
1.2.1 Behavior of posting data or code online	1.2 Behavior	
1.2.2 Behavior of posting study instruments		
1.2.3 Behavior of pre-registration		
1.3.1 Attitudes of posting data or code online	1.3 Attitudes	
1.3.2 Attitudes of posting study instruments		
1.3.3 Attitudes of pre-registration		
2.1.1 Descriptive norms of posting data or code online	2.1 Descriptive norms	2. Norms
2.1.2 Descriptive norms of pre-registration		
2.2.1 Prescriptive norms of posting data or code online	2.2 Prescriptive norms	
2.2.2 Prescriptive norms of pre-registration		
3.1.1 Awareness of posting data or code online	3.1 Posting data or code online	3. Overall Open Science
3.1.2 Behavior of posting data or code online		
3.1.3 Attitudes of posting data or code online		
3.1.4 Descriptive norms of posting data or code online		
3.1.5 Prescriptive norms of posting data or code online		
3.2.1 Awareness of posting study instruments	3.2 Posting study instruments	
3.2.2 Behavior of posting study instruments		
3.2.3 Attitudes of posting study instruments		
3.3.1 Awareness of pre-registration	3.3 Pre-registration	
3.3.2 Behavior of pre-registration		
3.3.3 Attitudes of pre-registration		
3.3.4 Descriptive norms of pre-registration		
3.3.5 Prescriptive norms of pre-registration		
4. Trustworthiness of literature		4. Trustworthiness of literature

Measures incorporated in Indices The table shows the mapping from measures (see Supplementary Table 8) to indices. Each sub-index is a simple average of measures, and each broad-index is a simple average of sub-indices.

We measure prescriptive norms using the following dynamic histogram programmed into Qualtrics:

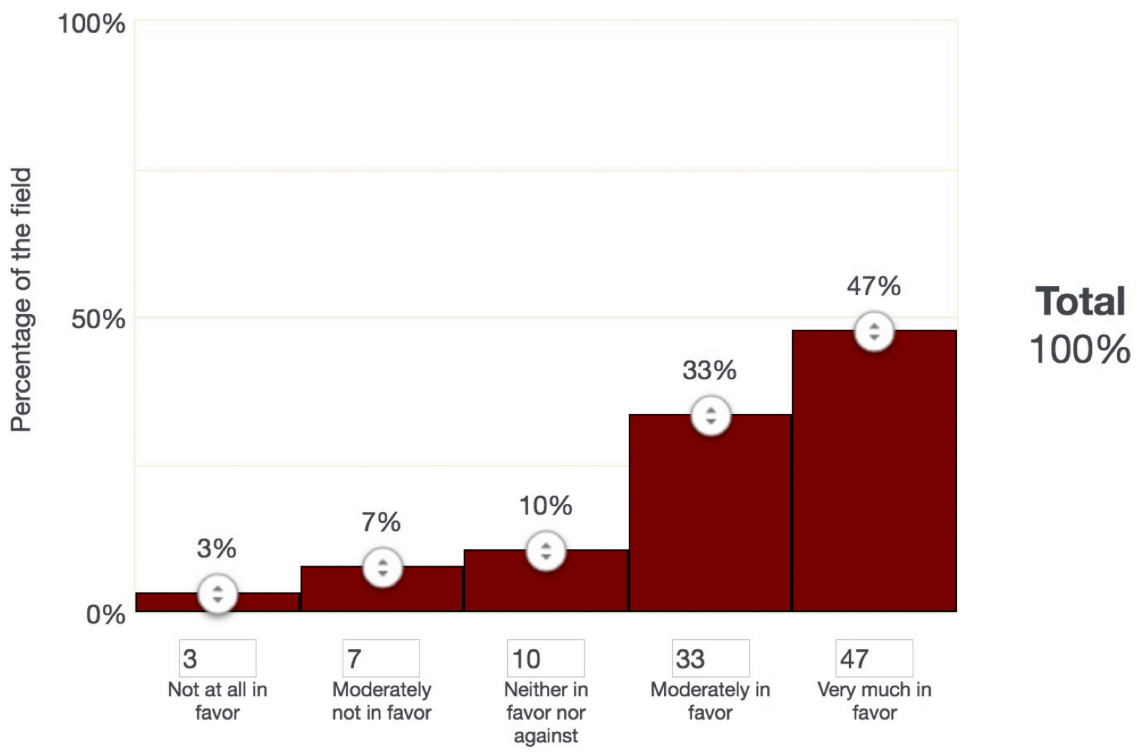

Supplementary Figure 1: **Dynamic Histogram used by survey respondents to indicate perceived support for open science practices.** This chart shows the dynamic histogram that survey respondents used to indicate perceived support for open science in their field. Bars need to add up to 100% for respondents to proceed in the survey.

Supplementary Table 8: Mapping Questions to Measures

Question	Measure	Rescaling and Aggregation
Have you ever heard of the practice of publicly posting data or code online for a completed study?	1.1.1 Awareness of posting data and code online	"No" → 0, "Yes" → 1
Approximately how many times have you publicly posted data or code online?	1.2.1 Behavior of posting data or code online	Question "Approximately. . . " coded as 0 → 0, anything ≥ 1 → 1<br>Question "Think about the last. . . " coded as "No" → 0, "Yes" → 1, "I have not published an empirical paper" → NA<br>Question "Do you encourage . . . " coded as ("No, and I don't plan to", "No, but I plan to in the future") → "0", ("Yes, I do") → "1"<br>Average over questions
Think about the last empirical paper you published. Have you publicly posted the data or code online for that paper?		
Do you encourage students to publicly post data or code online?		
To what extent do you believe that publicly posting data or code online is important for progress in [Discipline]?	1.3.1 Attitude of posting data or code online	Rescale from 1-5 to 0-1<br>Average over questions
What is your opinion of publicly posting data or code online?		
In your estimation, what percentage of researchers across the discipline of [Discipline] publicly post data or code online?	2.1.1 Descriptive norm of posting data or code online	Average over questions
In your estimation, what percentage of researchers in your sub-field of [Sub-discipline] publicly post data or code online?		
In your estimation, what is the distribution of opinion across the discipline of [Discipline] about publicly posting data or code online?	2.2.1 Prescriptive norm of posting data or code online	Calculate mean of distribution<br>Rescale from 1-5 to 0-1
In your estimation, what is the distribution of opinion in your sub-field of [Sub-discipline] about publicly posting data or code online?		
Have you ever heard of the practice of publicly posting study instruments online for a completed study?	1.1.2 Awareness of posting study instruments	"No" → 0, "Yes" → 1
Approximately how many times have you publicly posted study instruments online?	1.2.2 Behavior of posting study instruments	Question "Approximately. . . " coded as 0 → 0, anything ≥ 1 → 1<br>Question "Think about the last. . . " coded as "No" → 0, "Yes" → 1, "I have not published an empirical paper" → NA<br>Question "Do you encourage . . . " coded as ("No, and I don't plan to", "No, but I plan to in the future") → "0", ("Yes, I do") → "1"<br>Average over questions
Think about the last empirical paper you published. Have you publicly posted the study instruments online for that paper?		
Do you encourage students to publicly post study instruments online?		
To what extent do you believe that publicly posting study instruments online is important for progress in [Discipline]?	1.3.2 Attitude of posting study instruments	Rescale from 1-5 to 0-1<br>Average over questions
What is your opinion of publicly posting study instruments online?		

Have you ever heard of the practice of pre-registering hypotheses or analyses in advance of a study?	1.1.3 Awareness of pre-registration	Rescale from 1-5 to 0-1<br>Average over questions
Approximately how many times have you pre-registered hypotheses or analyses in advance of a study?<br>Think about the last empirical research you completed. Did you pre-register the hypotheses or analyses for that research?<br>Do you encourage students to pre-register hypotheses or analyses in advance of a study?	1.2.3 Behavior of pre-registration	Question "Approximately..." coded as 0 → 0, anything ≥ 1 → 1<br>Question "Think about the last..." coded as "No" → 0, "Yes" → 1, "I have not published an empirical paper" → NA<br>Question "Do you encourage ..." coded as ("No, and I don't plan to", "No, but I plan to in the future") → "0", ("Yes, I do") → "1"<br>Average over questions
To what extent do you believe that pre-registering hypotheses or analyses is important for progress in [Discipline]?<br>What is your opinion of pre-registering hypotheses or analyses?	1.3.3 Attitude of pre-registration	Rescale from 1-5 to 0-1<br>Average over questions
In your estimation, what percentage of researchers across the discipline of [Discipline] pre-register hypotheses or analyses in advance of a study?<br>In your estimation, what percentage of researchers in your sub-field of [Sub-discipline] pre-register hypotheses or analyses in advance of a study?	2.1.2 Descriptive norm of pre-registration	Rescale from 0-100 to 0-1<br>Average over questions
In your estimation, what is the distribution of opinion across the discipline of [Discipline] about pre-registering hypotheses or analyses in advance of a study?<br>In your estimation, what is the distribution of opinion in your sub-field of [Sub-discipline] about pre-registering hypotheses or analyses in advance of a study?	2.2.2 Prescriptive norm of pre-registration	Calculate mean of distribution<br>Rescale from 1-5 to 0-1
How confident are you that the influential research findings in [Discipline] would replicate?<br>When researchers run studies testing the canonical research findings in [Discipline], how confident are you that the studies will be able to replicate the canonical results?<br>When researchers run studies testing recent research findings in [Discipline], how confident are you that the studies will be able to replicate the recent results?<br>Think about the table of contents in the latest issue of [Discipline]'s top journal. How confident are you that the results of the studies will replicate?	4. Trustworthiness of literature	Rescale from 1-5 to 0-1<br>Average over questions

Questions incorporated in Measures The table shows the survey questions that are included in each measure. Each measure is then combined with other measures to produce indices (see Supplementary Table 7). In the cases where multiple questions are used in a single measure, how these questions are aggregated is also described.

SI 1.3 Response Rates

This section contains multiple summaries of the response rate. Overall, 6,231 individuals were invited in wave 1 and again in wave 2, of whom 6,114 were actually contacted (emails did not bounce). 3,257 responded in at least one wave, giving us an overall response rate of of 53%. 114 individuals explicitly opted out and 59 partially completed the survey (using only the most complete response from an individual across both waves). In addition to this sample, we invited 400 new participants in wave 2 for potential use in a future experimental paper; their responses are not used in any of the analyses for this paper. Wherever we do analyses specific to wave 2 responses, we only include individuals who were invited to both waves.

As shown in Supplementary Fig. 2, the response rate for published authors from psychology journals is somewhat lower than that for the other disciplines' journals. This may be due to the fact that a subset of psychologists often publish with scholars or clinicians from other fields who are less active empirical researchers, and therefore may be less likely to respond to an invitation to complete a survey focused on research methods. Consistent with this explanation, the response rate from authors who published in clinical and neuroscience-focused journals is considerably lower than the rate for social and developmental psychology journals (see Supplementary Fig. 6 for response rates by journal). Similarly, the response rate for authors who had published in macroeconomics journals is somewhat lower than the rate from other economics journals, possibly due to the greater share of articles based on theoretical or simulation approaches, rather than quantitative empirical data analysis, in those journals.

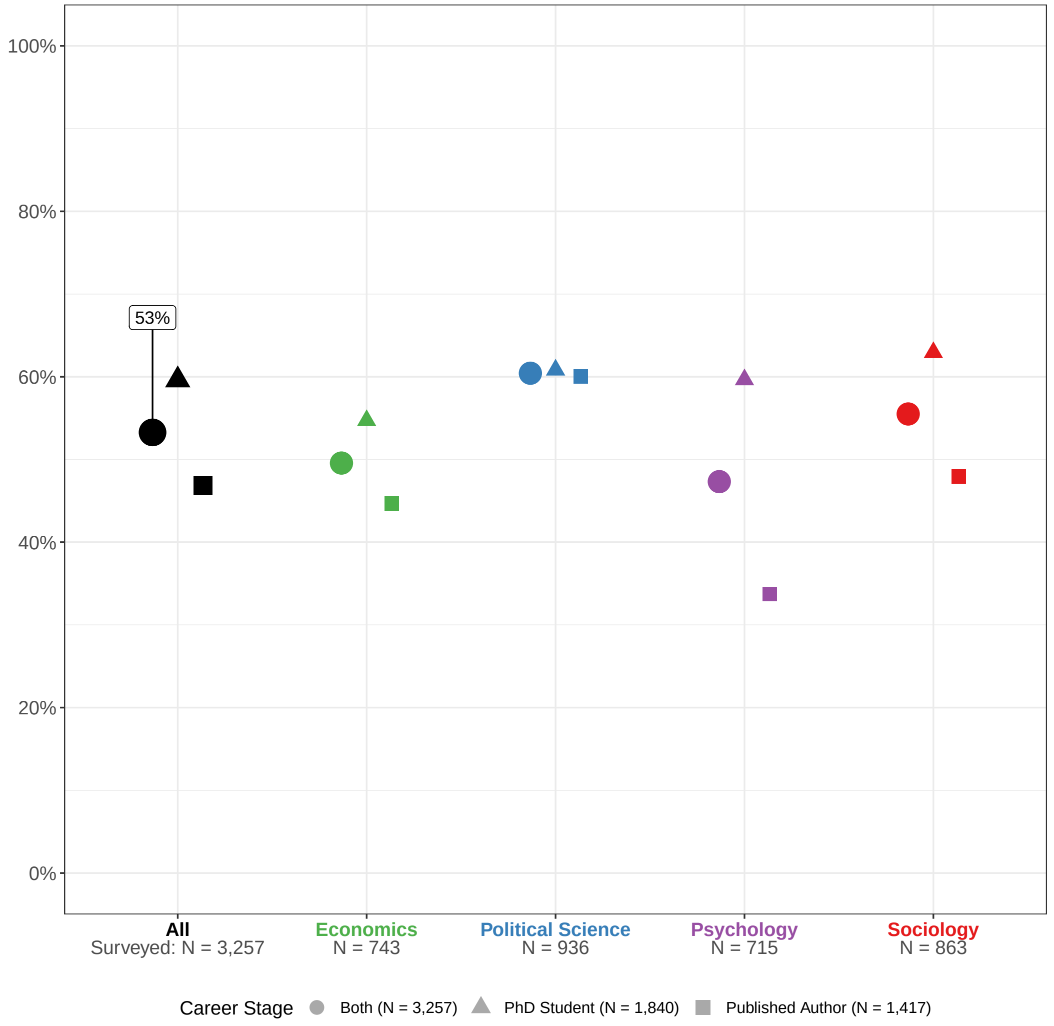

Supplementary Figure 2: **Response Rates Across Disciplines.** Response rates by discipline and by career stage (PhD student or published author). We contacted 6,114 researchers (6,231 researchers were invited via email but 117 could not be reached) across both survey rounds. Above figure consists of 3,257 respondents and 2,857 non-respondents, including 114 explicit opt-outs and 59 partially complete surveys, but excluding the 117 bounced emails. The number in parentheses in the legend is the number of responses received for a given category. An individual is classified as responding if they completed the wave 1 or wave 2 survey, or both.

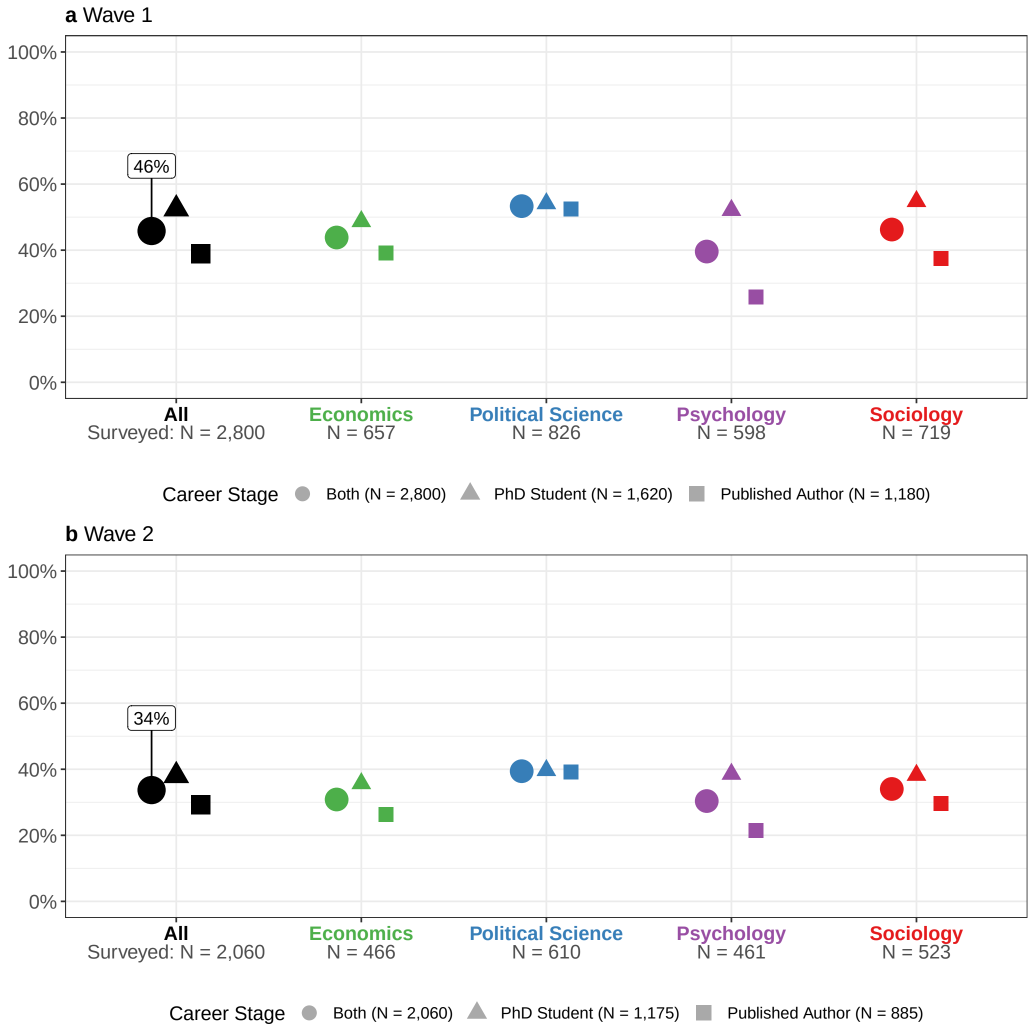

Supplementary Figure 3: **Response Rates by Survey Wave** Response rates by discipline and by career stage (PhD student or published author), in each survey wave are shown above. The top panel shows response rates for Survey Wave 1, conducted between April and August 2018. The bottom panel shows response rates for Survey Wave 2, conducted between March and July 2020. The number in parentheses in the legend is the number of responses received for a given category. An individual is classified as responding if they completed the wave 1 or wave 2 survey, respectively.

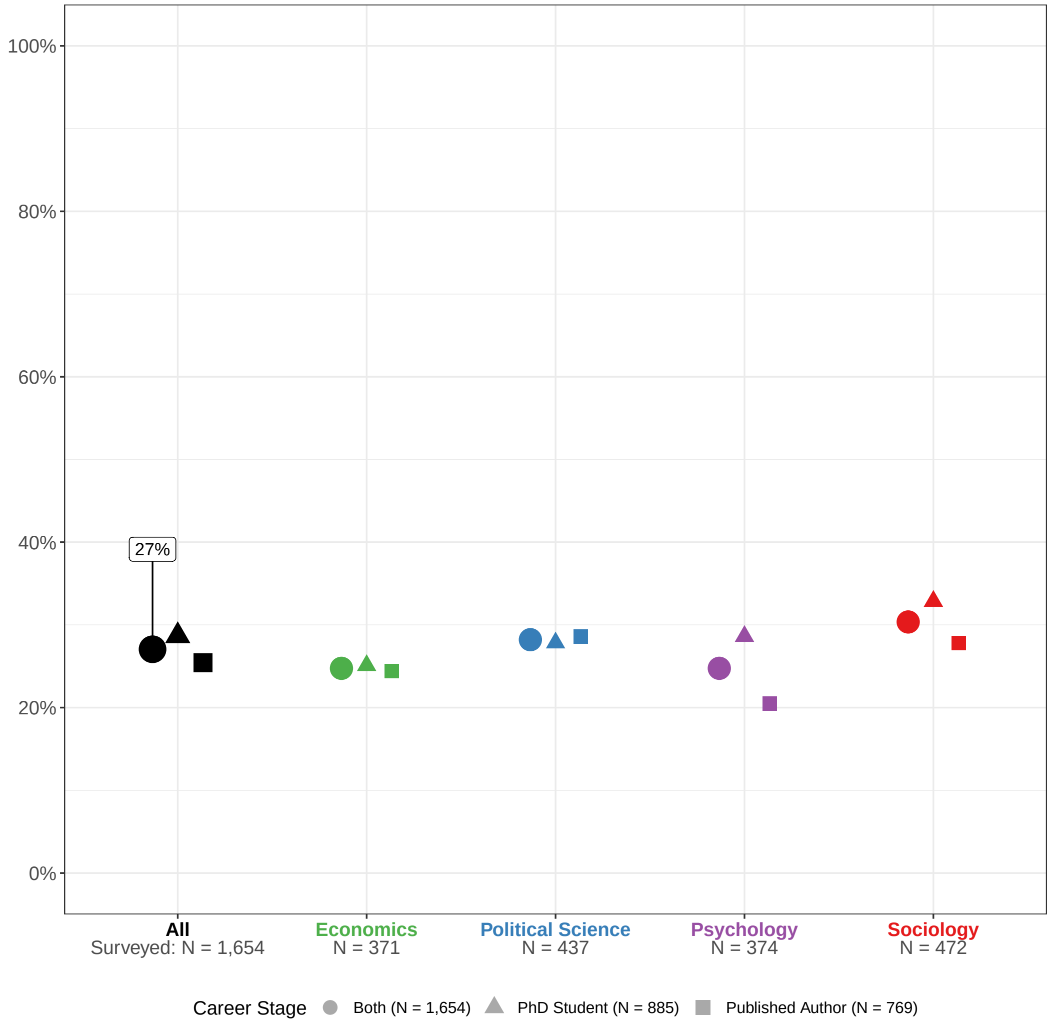

Supplementary Figure 4: **Response Rates Both Wave Completers** Response rates by discipline and by career stage (PhD student or published author), in each survey wave are shown above. An individual is classified as responding if they completed both the wave 1 and wave 2 survey. The response rate is calculated by dividing by all individuals invited to participate in the survey

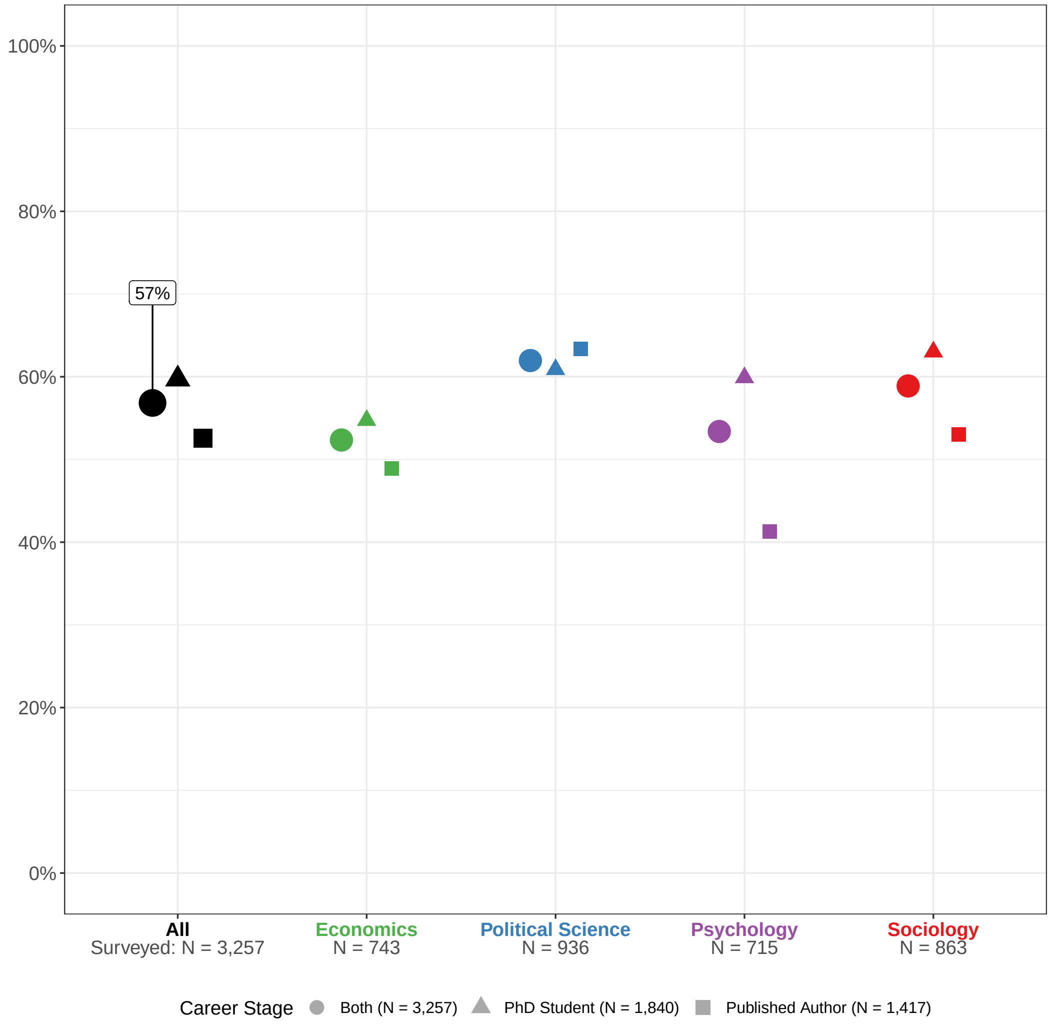

Supplementary Figure 5: **Response Rates are Higher in the United States and Canada Sample.** Response rates by discipline and by career stage (PhD student or published author). This figure shows the response rate by discipline and author status for all PhD students and published authors whose institution was based in the United States or Canada. The response rate is defined as an individual responding either to the wave 1 survey invite, or the wave 2 survey invite.

SI 1.4 Supplementary Audit and Online Data

In order to validate our survey responses and check for balance across respondents and non-respondents, we conducted an audit of our economics and some psychology published authors. Specifically, we randomly sampled i) all economics published authors who

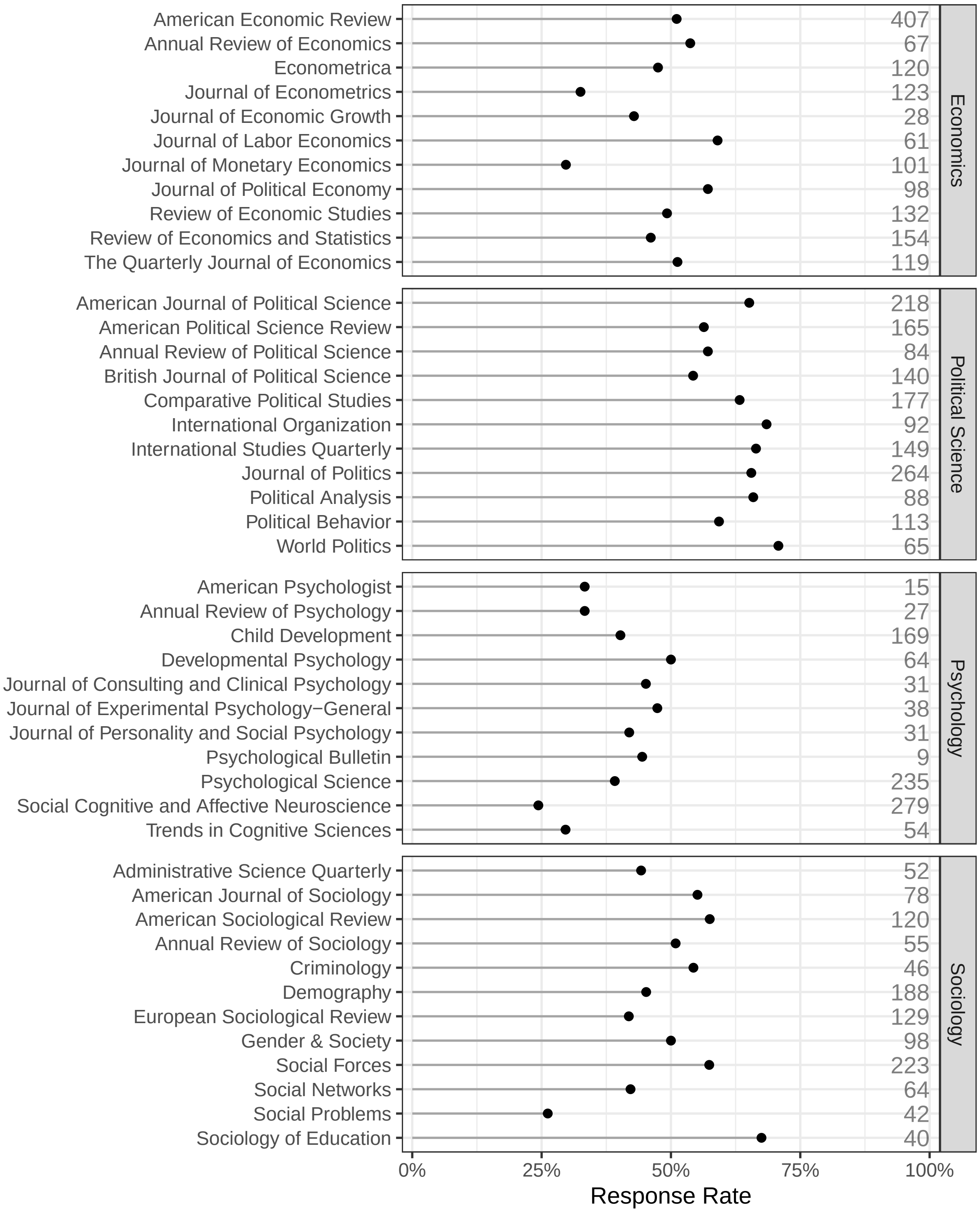

Supplementary Figure 6: **Response Rate by Journal.** This figure shows the response rate by journal for the universe of journals that were used as the sampling frame for published authors in this project. Each panel denotes the journals for a different discipline. Numbers in grey on the right hand side of the figures show the raw number of respondents from each journal. The published author sample is drawn from the universe of authors that published in one of the above journals during the timeframe 2014-2016. However, the published authors are matched to any journal in Supplementary Tables 2-5 that they published in during the period 2010-2016. Therefore the number of published authors in the table above is larger than the number of published authors in our sample.

completed our survey in both waves, ii) 150 economics published authors who were contacted but did not complete our survey in either wave and iii) 167 psychology published authors who were contacted in both waves, 119 of whom completed either wave of the survey. We chose to audit Economics and Psychology rather than all four due to project budget and staffing constraints. We expected that providing information on these fields would still be informative of the reliability of survey data for our entire sample.

We then conducted two audit activities. These audit activities were not pre-registered, but were considered important activities in order for us to reliably interpret the surveys. For all sampled individuals we conducted an audit of these authors' pre-registration and data posting behaviors using publicly available information. This is discussed in the Method section, and the protocol for this activity is the first subsection below. This audit activity was completed between March 15, 2019 and March 29, 2019 for wave 1. We then conducted a second round of audit activities after the second wave of surveys. This was completed between June and November 2020.

The second audit activity was conducted only for the non-respondent sample, and was completed between April 4, 2019 and April 15, 2019 for wave 1 and September to November 2020 for wave 2. In this activity, we used publicly available data sources to collect data on the primary subfield of these non-respondents. The protocol for this activity is below.

During the audits, subfields were collected and we manually categorised these subfields into one of four categories. The first of these was "Theory focused", which is categorised as any individual who listed Microeconomic Theory or Econometrics as a primary subfield. The second was "Macroeconomics/Finance", which was any author who listed Macroeconomics or Finance as a primary field. The third and fourth were "Development" and "Behavioral" economics. Finally, all other authors were categorised in the residual category. These

categories were chosen as it was suggested that the former two categories may have been less likely to have engaged previously in open science activity, whereas the latter two categories may have been more likely to have engaged in such behavior.

SI 1.4.1 Audit Protocol - Open Science behaviors

The goal of the audit is to identify whether a published author in the selected sample has (i) pre-registered an analysis or (ii) posted data or code for their projects. We use an author's last name as a keyword to search a set of popular open science websites used by scholars in economics and psychology.

General Procedure Since the collection of last names was fully automated in generating the sampling frame, auditors first verify whether an author's last name corresponds to a published author by looking for a university affiliation using a Google search.

The auditors then go to the websites listed below, and search by last name only. They look through the search results and try to identify the published author using their first name or affiliation. Then, following the link associated with an identified author, auditors look for a (i) pre-analysis plan or (ii) posted data or code on the websites. As soon as a match is found, auditors stop searching and record the match and a link to the matched page. If no match can be found, the auditors record that no match was found.

After the second wave, auditors also searched for the 4-5 most recent publications by authors, and checked journal websites for evidence of posting data or code directly.

Note that this audit process is in contrast to the procedure outlined by [2, 3], who follow a more tightly defined search process that focuses on mentions of data and pre-registration (as well as other open science practices) within journal articles themselves. While both audit methods have benefits, we did find that our more wide-ranging audit activity seemed

to uncover a larger number of open science practices that may have been missed in more narrowly defined audit protocols.

Websites for posting data or code online

- Dataverse.org
- Authors' personal websites
- OSF (wave 2)
- Journal websites of recent publications (wave 2)

Websites for pre-registering analysis (PAP)

- SocialScienceRegistry.org (AEA RCT registry). Details of some pre-analysis plans may not be visible to the public, but we still count those as having pre-registered.
- OSF.io
- Authors' personal websites

SI 1.4.2 Audit Protocol - Author Subfield

The goal of this activity is to collect data on the primary subfields of Economics published authors that did not complete the survey. The following steps are followed to complete this activity:

- Go to the author's webpage. Record subfields information if subfields of interest are listed on the homepage or another part of the webpage.
- Open the author's CV. Record any subfields that are listed on the author's CV.

SI 1.5 Supplemetary material on Reliability and Representativeness of Survey Responses

To assess the representativeness of those who responded to our survey of our selected sample, as well as the reliability of our survey results, we utilize the data collected in the manual and automated audits of open science behavior described in section SI 1.4.1 above. These audits allowed us to collect data on objective measures of open science behavior for a random sample of published author respondents and non-respondents from economics and psychology, such as whether the respondent had previously posted data or code online. The goal of these audits were threefold. First, by comparing manual audit data to self-reports we can assess the accuracy of self-reported data. Second, using both manual audits and web-based scraping audits allowed us to assess the degree to which web-based scraping approaches might be able to determine broad trends in open science. Finally, by auditing both survey respondents and non-respondents we are able to assess the degree to which there is differential selection along observable dimensions into our sample.

SI 1.5.1 Reliability

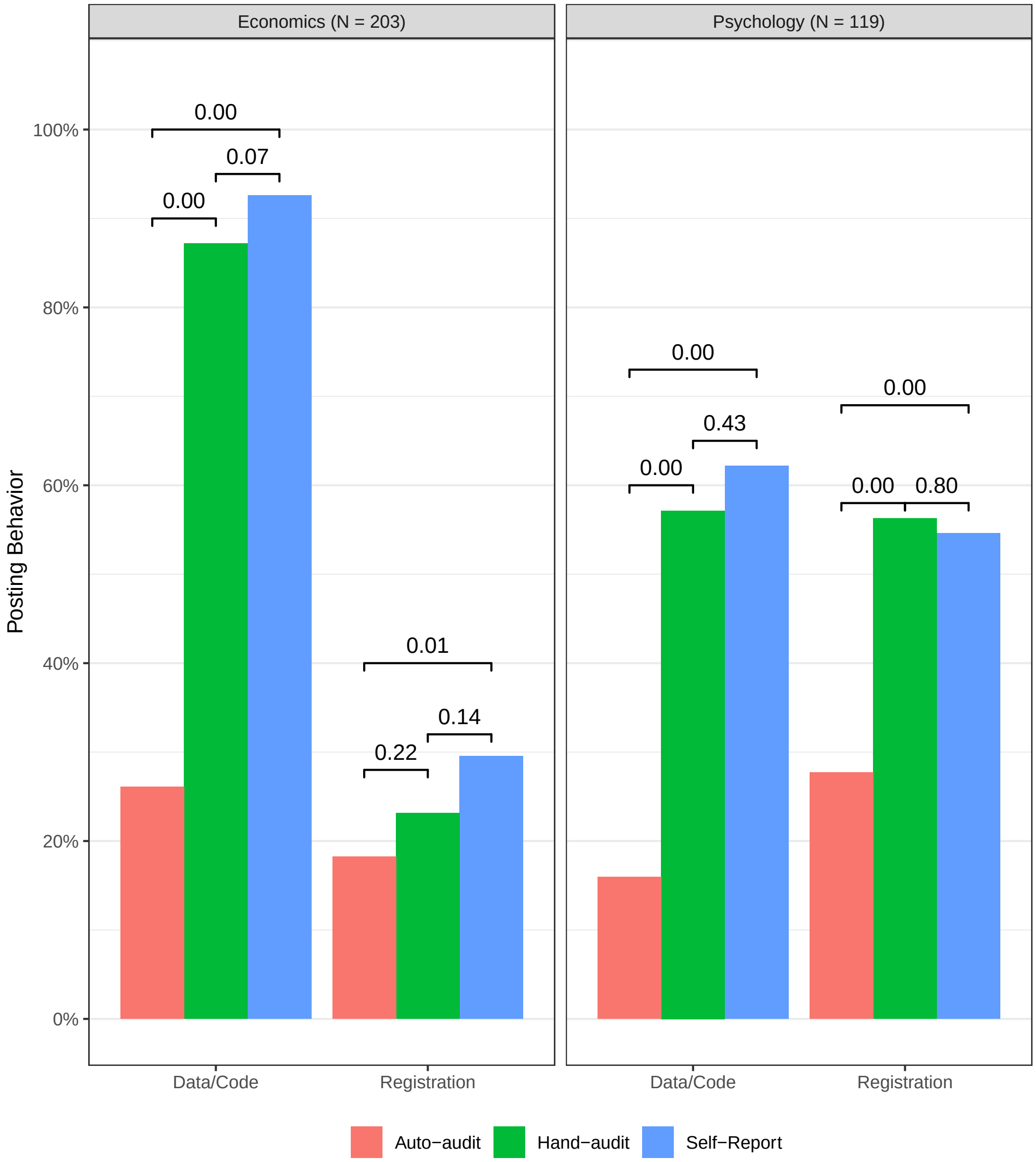

Supplementary Figure 7: **Correspondence between Survey data, manual audits, and automated audits** This figure compares the extent of posting of data or code found in i) the electronic audit, ii) the hand-audit, and iii) survey self-reports. The sample is restricted to a randomly selected subset of published authors that responded to Survey Wave 2 (N = 203 economics published authors, N = 199 psychology published authors, total N = 322). The red bar shows the proportion of the sample who we found engaged in a behavior in our automated audit of major code and pre-registration repositories. The green bar shows the proportion of the sample we found engaged in a behavior in our manual hand audits. The blue bar shows the proportion of the sample that self-reported ever engaging in a behavior in our surveys. The numbers above the bars correspond to p-values of two-sided Mann-Whitney tests without adjustments for multiple comparisons.

SI 1.5.2 Representativeness

To model selection into the sample, we predict the propensity of response based on covariates. We also assess the robustness of this approach by using multiple models of the response probability. In employing these various propensity score-based adjustments, we assume i) that selection into our sample (after conditioning on some set of covariates), is unrelated to our outcomes of interest[2] and ii) we are able to model selection into our sample based on covariates of our sample. In this case we use the Hajek esimator:

$$\mu_{hajek} = \frac{\sum_{i=1}^{N} \frac{1}{\hat{p}(z)} y_i}{\sum_{i=1}^{N} \frac{1}{\hat{p}(z)}}$$

Where N is the sample size of the response data set, $\hat{p}(z)$ is the estimated response probability of individual i with covariates z and y_i is the outcome data for this individual.

Both assumptions, however, must be true for the estimator to be consistent. That is, the model of the propensity to respond i) must be modelled correctly and ii) be unrelated to outcomes.

For the first point, we collect a rich set of covariates related to our published author sample to model selection into our sample. In particular, from publicly available sources we gathered data regarding the individual's i) publication track record between 2014 and 2016, ii) region and institution of work and iii) institution ranking. We also generated a probabilistic measure of respondent's gender using an algorithm that predicts gender from a respondent's name and used this predictor in our selection model. We note that this predictor is only an imperfect measure of respondent's self reported gender. We also

[2] Specifically for a sample where only a subset respond (R), and we observe covariates X for all individuals but outcomes Y only for the responders we assume that $\mathbb{P}(S=1|Y,X) = \mathbb{P}(S=1|X)$

assess the robustness of our model by using multiple models of the response probability. In particular, first we use the covariate balancing propensity score of [4], a widely employed method that has been shown to improve the performance of propensity scores. Moreover, given the large number of covariates in our dataset, we use a high dimensional version of the estimator [5]. Secondly, we also use machine learning tools to estimate the probability of response into our sample, which have been shown in some settings to improve the performance of traditional propensity score estimators, particularly when the predictors are high dimensional and there may be non-linearities in the selection model [6, 7].[3]

Rather than taking a stand on the optimal algorithm, we follow the approach of [8] and employ an ensemble method that produces a weighted average of several machine learning algorithms (random forests, lasso/ridge and bayesian glm), a methodology that has been shown to reduce bias in the event the underlying selection model is misspecified. While assessing model performance for propensity scores is challenging [6] our model of response probabilities generates an AUC[4] of 0.63. This is similar to other models of human decision-making (for instance, [9] predict job hiring model using features gathered from individual's CV's: their algorithm produces an AUC of 0.67) although somewhat low compared to what is considered a model with a high degree of predictive power. [5] Finally, for comparison we also estimate weights using a random forest algorithm.

The correlation between the weights generated by each method ranges from 0.25-0.65. Generally, results adjusting for selection under various propensity score models are very

[3]For instance, random forest estimators may offer improvements if there are non-linearities the selection model.

[4]One way to interpret the Area under the curve is as the probability that for two randomly selected individuals, the algorithm will correctly rank the response probability. Therefore, an algorithm produces predictions no better than chance if the AUC is 0.5.

[5]Though there is not a threshold value, a model generating an AUC of .75-0.85 may be considered to perform well ([10], [9])

similar to the unadjusted results. See Supplementary Tables 18-21 for the full set of adjusted results.

We also provide some evidence that selection into our sample is on covariates rather than outcomes, by again drawing on our hand audit of economists for evidence that this assumption may be correct. In particular, Supplementary Table 15 shows that among economists there is apparent selection on behavior (particularly pre-registration) into our sample. However, once we adjust for author's subfields, we find little credible evidence that respondents and non-respondents differ greatly in behavior.

We also use our audit data to generate a third simple estimate of actual posting data/code and pre-registration behavior for the population of interest, using a weighted average of validated behavior for survey respondents and non-respondents. This method also generates estimates that are qualitatively consistent with our survey results.

Weighting economist respondents and non-respondents behaviors by their share of the invited population would suggest that the population level of posting data or code is around 75%, and pre-registration is around 14%. Even under these assumptions our qualitative results are unchanged: posting data or code is high among economists and this is underappreciated by other economists, whereas the extent of pre-registration is much lower and the extent of misperception much less. A similar exercise for psychologists (not shown) uses a similar method to estimate that 55% of the psychologist published authors have posted data and 45% have pre-registered, slightly lower than our unadjusted estimates but still substantially higher than psychologists perceptions.

Finally, note that these estimates are not our preferred estimates of behavior, since only a sample of respondents and non-respondents were randomly sampled for auditing and therefore the level of behavior may reflect some sampling variation. However, it is

reassuring that even accounting for selection into our sample in this manner does not qualitatively change our findings.

SI 1.5.2.1 Verifying Survey Responses

Supplementary Table 9: Verifying Survey Responses in Survey Wave 1: Posting Data/Code for Economics Published Authors

		Audit - Posted Data			
		Yes		No	
**Survey - Posted Data**	Yes	59.73%	(178)	24.50%	(73)
	No	3.69%	(11)	12.08%	(36)
**Survey/Audit Alignment**	71.81%				

This table shows survey responses and our audit findings for wave 1 Economics published author survey respondents. Rows capture whether a respondent indicated they had previously posted data/code online, or not. Columns capture whether we find data/code posted for each individual, or not. Entries on the diagonal indicate respondents for whom their survey response matches our audit findings. The audit was completed between March 15 and March 29, 2019.

Supplementary Table 10: Verifying Survey Responses in Survey Wave 1: Pre-registration for Economics Published Authors

		Audit - Pre-registration			
		Yes		No	
**Survey - Pre-registration**	Yes	13.76%	(41)	4.70%	(14)
	No	5.03%	(15)	76.51%	(228)
**Survey/Audit Alignment**	90.27%				

This table shows survey responses and our audit findings for wave 1 Economics published author survey respondents. Rows capture whether a respondent indicated they had previously pre-registered, or not. Columns capture whether we find an online pre-registration posted for each individual, or not. Entries on the diagonal indicate respondents for whom their survey response matches our audit findings. The audit was completed between March 15 and March 29, 2019.

Supplementary Table 11: Verifying Survey Responses in Survey Wave 2: Posting Data/Code for Economics Published Authors

		**Audit - Posted Data**			
		Yes		No	
**Survey - Posted Data**	Yes	85.20%	(167)	6.12%	(12)
	No	2.55%	(5)	6.12%	(12)
**Survey/Audit Alignment**	91.33%				

This table shows survey responses and our audit findings for wave 2 Economics published authors survey respondents. Rows capture whether a respondent indicated they had previously posted data/code online, or not. Columns capture whether we find data/code posted for each individual, or not. Entries on the diagonal indicate respondents for whom their survey response matches our audit findings. The audit was completed between June and November 2020.

Supplementary Table 12: Verifying Survey Responses in Survey Wave 2: Pre-registration for Economics Published Authors

		**Audit - Pre-registration**			
		Yes		No	
**Survey - Pre-registration**	Yes	20.92%	(41)	6.63%	(13)
	No	2.04%	(4)	70.41%	(138)
**Survey/Audit Alignment**	91.33%				

This table shows survey responses and our audit findings for wave 2 Economics published authors survey respondents. Rows capture whether a respondent indicated they had previously pre-registered, or not. Columns capture whether we find an online pre-registration posted for each individual, or not. Entries on the diagonal indicate respondents for whom their survey response matches our audit findings. The audit was completed between June and November 2020.

Supplementary Table 13: Verifying Survey Responses in Survey Wave 2: Posting data/code for Psychology Published Authors

		Audit - Posted Data			
		Yes		No	
**Survey - Posted Data**	Yes	48.74%	(58)	13.45%	(16)
	No	8.40%	(10)	29.41%	(35)
**Survey/Audit Alignment**	78.15%				

This table shows survey responses and our audit findings for a randomly selected subset of our Psychology published author respondents. Rows capture whether a respondent indicated they had previously posted data/code online, or not. Columns capture whether we find data posted for each individual, or not. Entries on the diagonal indicate respondents for whom their survey response matches our audit findings. The audit was completed between June and November 2020.

Supplementary Table 14: Verifying Survey Responses in Survey Wave 2: Pre-registration for Psychology Published Authors

		Audit - Registered			
		Yes		No	
**Survey - Registered**	Yes	42.86%	(51)	11.76%	(14)
	No	13.45%	(16)	31.93%	(38)
**Survey/Audit Alignment**	74.79%				

This table shows survey responses and our audit findings for a randomly selected subset of our Psychology published author respondents. Rows capture whether a respondent indicated they had previously pre-registered, or not. Columns capture whether we find a pre-registration for each individual, or not. Entries on the diagonal indicate respondents for whom their survey response matches our audit findings. The audit was completed between June and November 2020.

SI 1.5.2.2 Differences Between Respondents and Non-Respondents

Supplementary Table 15: Differences in Open Science Behavior by respondents and non-respondents, with and without controls for observable characteristics

	Posting			Registration			Any			Completed Survey
	(1)	(2)	(3)	(4)	(5)	(6)	(7)	(8)	(9)	(10)
Survey Respondent	0.07*	0.04	0.01	0.15***	0.06***	0.04	0.08**	0.05	0.02	
	(0.04)	(0.04)	(0.04)	(0.03)	(0.02)	(0.02)	(0.04)	(0.04)	(0.04)	
Theory/Metrics		-0.32***	-0.32***		-0.04*	-0.04		-0.30***	-0.30***	-0.06
		(0.05)	(0.05)		(0.03)	(0.03)		(0.05)	(0.05)	(0.05)
Macro		0.01	-0.02		-0.07***	-0.09***		-0.00	-0.04	-0.14***
		(0.04)	(0.05)		(0.02)	(0.03)		(0.04)	(0.04)	(0.05)
Development		0.11***	0.14***		0.63***	0.62***		0.14***	0.17***	0.19***
		(0.04)	(0.04)		(0.06)	(0.07)		(0.03)	(0.04)	(0.06)
Behavioral		0.07	0.03		0.51***	0.44***		0.11**	0.06	0.28***
		(0.07)	(0.08)		(0.10)	(0.10)		(0.05)	(0.06)	(0.07)
Observations	565	565	545	565	565	545	565	565	545	565
Constant	0.72	0.81		0.06	0.07		0.73	0.80		0.63
Controls	No	No	Yes	No	No	Yes	No	No	Yes	No

The table presents regression results for regressions of audited open science behavior on indicators for whether an individual responded to our survey, as well as individual level control variables. The sample for this table is all Economics published authors who completed the survey across wave 1 and wave 2, plus a randomly selected subsample of non-respondent economics published authors. Outcomes in columns 1-3 are whether the individual posted data, 4-6 are whether the individual pre-registered, and 7-9 are whether the individual conducted any practice. Column 10 shows the probability of responding by subfield (note that this is not the response rate as only a randomly selected subset of non-respondents is included). All outcome data comes from manual audits. Survey respondent is an indicator for whether the individual completed our survey across one of the two waves. The additional controls shown are subfields of research. Controls include geographic work region, indicators for numbers of times published in particular journals and job classification. Standard errors are in parentheses. * indicates significance at the 10% level, ** indicates significance at the 5% level and *** indicates significance at the 1% level from a two-sided t-test. There is no adjustment for multiple testing.

Supplementary Table 16: Differences in Observables for those Completing and Not Completing Survey

Variable	Overall (1)	Respondent (2)	Nonrespondent (3)	Difference (2) - (3)
**All**				
Publication Count (right winsorized)	2.08	2.21	2	0.22 (4.16)***
USA and Canada	0.68	0.76	0.63	0.13 (7.62)***
N	3027	1180	1847	
**Economics**				
Publication Count (right winsorized)	2.3	2.37	2.25	0.12 (1.13)
USA and Canada	0.66	0.72	0.61	0.11 (3.07)***
N	766	300	466	
**Political Science**				
Publication Count (right winsorized)	2.39	2.45	2.32	0.13 (1.22)
USA and Canada	0.76	0.8	0.72	0.08 (2.54)**
N	773	406	367	
**Psychology**				
Publication Count (right winsorized)	1.73	1.81	1.7	0.11 (1.01)
USA and Canada	0.59	0.72	0.54	0.18 (4.48)***
N	718	185	533	
**Sociology**				
Publication Count (right winsorized)	1.89	1.98	1.84	0.14 (1.49)
USA and Canada	0.71	0.77	0.68	0.09 (2.83)***
N	770	289	481	

This table presents differences in means for the number of publications and geographic location of the university for published authors who did and did not complete the survey. The "Difference" column shows differences in means and t-statistics in parentheses. * indicates significance at the 10% level, ** indicates significance at the 5% level and *** indicates significance at the 1% level from a two-sided t-test. There is no adjustment for multiple testing.

Supplementary Table 17: Characteristics of those Completing Survey

	Completed Survey				
	All	Economics	Psychology	Political Science	Sociology
	(1)	(2)	(3)	(4)	(5)
USA and Canada	0.170***	0.117***	0.180***	0.133***	0.175***
	(0.02)	(0.04)	(0.04)	(0.04)	(0.04)
Publication Count	0.020***	0.004	0.006	0.010	0.033**
(right winsorized)	(0.01)	(0.01)	(0.02)	(0.01)	(0.01)
Constant	0.313***	0.363***	0.220***	0.476***	0.299***
	(0.02)	(0.04)	(0.04)	(0.04)	(0.04)
Observations	2,983	708	753	763	759

This table presents regression coefficients and standard errors from ordinary least squares regressions. The outcome variable in each regression is an indicator variable for whether the individual contacted completed the survey. The covariates are observable characteristics of the individual contacted. The sample is limited to published authors. * indicates significance at the 10% level, ** indicates significance at the 5% level and *** indicates significance at the 1% level from a two-sided t-test. There is no adjustment for multiple testing.

SI 1.5.2.3 Propensity score weight estimates

Supplementary Table 18: Propensity score weighted estimates, Attitudes Awareness Behavior

	Unadjusted	HDCBPS	Super Learner	Random Forest
**All Published Authors**	(1)	(2)	(3)	(4)
Overall personal support	0.730	0.729	0.729	0.726
Behavior	0.482	0.479	0.480	0.471
Awareness	0.891	0.888	0.888	0.896
Attitude	0.816	0.820	0.817	0.812
**Economics Published Authors**				
Overall personal support	0.747	0.743	0.742	0.738
Behavior	0.534	0.531	0.531	0.525
Awareness	0.872	0.866	0.863	0.853
Attitude	0.833	0.833	0.833	0.834
**Psychology Published Authors**				
Overall personal support	0.780	0.778	0.777	0.778
Behavior	0.581	0.577	0.576	0.580
Awareness	0.927	0.923	0.924	0.923
Attitude	0.832	0.834	0.832	0.831
**Political Science Published Authors**				
Overall personal support	0.748	0.754	0.752	0.736
Behavior	0.478	0.489	0.489	0.463
Awareness	0.921	0.923	0.924	0.932
Attitude	0.845	0.849	0.844	0.811
**Sociology Published Authors**				
Overall personal support	0.637	0.643	0.639	0.652
Behavior	0.309	0.320	0.316	0.339
Awareness	0.843	0.843	0.840	0.839
Attitude	0.759	0.764	0.762	0.777

This table presents estimates of the outcomes, adjusted using propensity score weighting. Sample means are adjusted using Hajek weights. Propensity scores are estimated using three methods. Col. 2 estimates the probability of responding to the survey using high-dimensional covariate-balancing propensity score (Ning et al 2018). Col. 3 estimates the propensity score using an ensemble learner. Col. 4 estimates the propensity score using a random forest algorithm. Sample estimates are computed for different Disciplines. The sample is restricted to published authors, as we only have a rich array of covariates to predict survey response at this career stage.

Supplementary Table 19: Propensity score weighted estimates, Adoption

	Unadjusted	HDCBPS	Super Learner	Random Forest
**All Published Authors**	(1)	(2)	(3)	(4)
Any	0.866	0.869	0.868	0.881
Posting data or code online	0.770	0.767	0.764	0.788
Posting study instruments online	0.540	0.544	0.540	0.574
Pre-registering hypotheses or analyses	0.272	0.285	0.284	0.266
**Economics Published Authors**				
Any	0.961	0.955	0.954	0.951
Posting data or code online	0.935	0.927	0.927	0.925
Posting study instruments online	0.431	0.427	0.413	0.389
Pre-registering hypotheses or analyses	0.224	0.214	0.214	0.204
**Political Science Published Authors**				
Any	0.928	0.928	0.925	0.925
Posting data or code online	0.894	0.890	0.887	0.881
Posting study instruments online	0.619	0.610	0.609	0.611
Pre-registering hypotheses or analyses	0.292	0.289	0.286	0.279
**Psychology Published Authors**				
Any	0.873	0.878	0.884	0.907
Posting data or code online	0.669	0.693	0.683	0.786
Posting study instruments online	0.712	0.722	0.728	0.783
Pre-registering hypotheses or analyses	0.551	0.552	0.562	0.373
**Sociology Published Authors**				
Any	0.673	0.695	0.686	0.729
Posting data or code online	0.485	0.515	0.500	0.553
Posting study instruments online	0.469	0.480	0.473	0.488
Pre-registering hypotheses or analyses	0.138	0.139	0.140	0.189

This table presents estimates of the outcomes, adjusted using propensity score weighting. Sample means are adjusted using Hajek weights. Propensity scores are estimated using three methods. Col. 2 estimates the probability of responding to the survey using high-dimensional covariate-balancing propensity score (Ning et al 2018). Col. 3 estimates the propensity score using an ensemble learner. Col. 4 estimates the propensity score using a random forest algorithm. Sample estimates are computed for different Disciplines. The sample is restricted to published authors, as we only have a rich array of covariates to predict survey responses at this career stage.

Supplementary Table 20: Propensity score weighted estimates, Pre-registering hypotheses or analyses

**All Published Authors**	Unadjusted (1)	HDCBPS (2)	Super Learner (3)	Random Forest (4)
**Behavior**	0.272	0.281	0.279	0.225
**Attitudes**				
Very much in favor	0.224	0.245	0.236	0.220
Moderately in favor	0.320	0.320	0.318	0.288
Neither in favor nor against	0.290	0.283	0.289	0.370
Moderately not in favor	0.113	0.102	0.106	0.081
Not at all in favor	0.053	0.050	0.051	0.041
**Economics Published Authors**				
**Behavior**	0.223	0.209	0.210	0.196
**Attitudes**				
Very much in favor	0.193	0.189	0.191	0.193
Moderately in favor	0.354	0.362	0.357	0.350
Neither in favor nor against	0.280	0.277	0.280	0.283
Moderately not in favor	0.122	0.121	0.122	0.123
Not at all in favor	0.051	0.051	0.051	0.052
**Psychology Published Authors**				
**Behavior**	0.299	0.289	0.291	0.286
**Attitudes**				
Very much in favor	0.205	0.211	0.205	0.202
Moderately in favor	0.345	0.344	0.344	0.353
Neither in favor nor against	0.275	0.275	0.277	0.267
Moderately not in favor	0.116	0.107	0.110	0.107
Not at all in favor	0.059	0.063	0.063	0.071
**Political Science Published Authors**				
**Behavior**	0.498	0.502	0.505	0.253
**Attitudes**				
Very much in favor	0.412	0.424	0.412	0.271
Moderately in favor	0.325	0.324	0.317	0.244
Neither in favor nor against	0.210	0.205	0.221	0.458
Moderately not in favor	0.041	0.038	0.038	0.022
Not at all in favor	0.012	0.009	0.012	0.006
**Sociology Published Authors**				
**Behavior**	0.129	0.131	0.130	0.146
**Attitudes**				
Very much in favor	0.145	0.154	0.150	0.163
Moderately in favor	0.250	0.250	0.251	0.257
Neither in favor nor against	0.378	0.377	0.376	0.374
Moderately not in favor	0.151	0.144	0.147	0.135
Not at all in favor	0.076	0.075	0.076	0.071

This table presents estimates of the outcomes, adjusted using propensity score weighting. Sample means are adjusted using Hajek weights. Propensity scores are estimated using three methods. Col. 2 estimates the probability of responding to the survey using high-dimensional covariate-balancing propensity score (Ning et al 2018). Col. 3 estimates the propensity score using an ensemble learner. Col. 4 estimates the propensity score using a random forest algorithm. Sample estimates are computed for different Disciplines. The sample is restricted to published authors, as we only have a rich array of covariates to predict survey responses at this career stage.

Supplementary Table 21: Propensity score weighted estimates, Posting data or code online

**All Published Authors**	Unadjusted (1)	HDCBPS (2)	Super Learner (3)	Random Forest (4)
**Behavior**	0.730	0.722	0.721	0.663
**Attitudes**				
Very much in favor	0.603	0.592	0.592	0.513
Moderately in favor	0.281	0.288	0.286	0.389
Neither in favor nor against	0.081	0.082	0.084	0.064
Moderately not in favor	0.033	0.035	0.035	0.032
Not at all in favor	0.002	0.002	0.002	0.001
**Economics Published Authors**				
**Behavior**	0.874	0.865	0.867	0.862
**Attitudes**				
Very much in favor	0.748	0.749	0.751	0.758
Moderately in favor	0.231	0.230	0.228	0.228
Neither in favor nor against	0.015	0.015	0.015	0.010
Moderately not in favor	0.006	0.006	0.006	0.005
Not at all in favor	0.000	0.000	0.000	0.000
**Psychology Published Authors**				
**Behavior**	0.872	0.870	0.868	0.866
**Attitudes**				
Very much in favor	0.741	0.745	0.739	0.734
Moderately in favor	0.214	0.209	0.213	0.216
Neither in favor nor against	0.028	0.030	0.030	0.031
Moderately not in favor	0.015	0.014	0.016	0.017
Not at all in favor	0.002	0.002	0.002	0.003
**Political Science Published Authors**				
**Behavior**	0.637	0.657	0.646	0.539
**Attitudes**				
Very much in favor	0.496	0.502	0.489	0.356
Moderately in favor	0.314	0.321	0.322	0.546
Neither in favor nor against	0.112	0.101	0.114	0.053
Moderately not in favor	0.074	0.072	0.072	0.044
Not at all in favor	0.004	0.003	0.003	0.001
**Sociology Published Authors**				
**Behavior**	0.468	0.490	0.477	0.532
**Attitudes**				
Very much in favor	0.352	0.365	0.361	0.395
Moderately in favor	0.395	0.397	0.394	0.392
Neither in favor nor against	0.195	0.186	0.190	0.165
Moderately not in favor	0.055	0.050	0.053	0.046
Not at all in favor	0.003	0.003	0.003	0.002

This table presents estimates of the outcomes, adjusted using propensity score weighting. Sample means are adjusted using Hajek weights. Propensity scores are estimated using three methods. Col. 2 estimates the probability of responding to the survey using high-dimensional covariate-balancing propensity score (Ning et al 2018). Col. 3 estimates the propensity score using an ensemble learner. Col. 4 estimates the propensity score using a random forest algorithm. Sample estimates are computed for different Disciplines. The sample is restricted to published authors, as we only have a rich array of covariates to predict survey responses at this career stage.

SI 2 Supplementary Results

SI 2.0.1 Sub Indices, Attitudes Awareness Behavior, Number of Responses

Supplementary Table 22: Sub Indices, Attitudes, Behavior and Norms, Number of Responses Across Both Waves

Discipline	Attitude	Awareness	Behavior	Overall
**All Respondents**				
All	3178	3179	3163	3179
Economics	724	724	722	724
Political Science	909	909	906	909
Psychology	708	708	707	708
Sociology	837	838	828	838
**PhD Students**				
All	1795	1795	1783	1795
Economics	387	387	386	387
Political Science	450	450	447	450
Psychology	465	465	464	465
Sociology	493	493	486	493
**Published Authors**				
All	1383	1384	1380	1384
Economics	337	337	336	337
Political Science	459	459	459	459
Psychology	243	243	243	243
Sociology	344	345	342	345

Supplementary Table 23: Attitudes, Behavior and Norms, Number of Responses Across Wave 1

Discipline	Attitude	Awareness	Behavior	Overall
**All Respondents**				
All	2706	2707	2667	2707
Economics	619	619	614	619
Political Science	788	788	775	788
Psychology	590	590	587	590
Sociology	709	710	691	710
**PhD Students**				
All	1567	1567	1533	1567
Economics	337	337	332	337
Political Science	393	393	381	393
Psychology	407	407	404	407
Sociology	430	430	416	430
**Published Authors**				
All	1139	1140	1134	1140
Economics	282	282	282	282
Political Science	395	395	394	395
Psychology	183	183	183	183
Sociology	279	280	275	280

Supplementary Table 24: Attitudes, Behavior and Norms, Number of Responses Across Wave 2

Discipline	Attitude	Awareness	Behavior	Overall
**All Respondents**				
All	2050	2050	2047	2050
Economics	463	463	462	463
Political Science	608	608	608	608
Psychology	459	459	459	459
Sociology	520	520	518	520
**PhD Students**				
All	1167	1167	1166	1167
Economics	259	259	259	259
Political Science	304	304	304	304
Psychology	304	304	304	304
Sociology	300	300	299	300
**Published Authors**				
All	883	883	881	883
Economics	204	204	203	204
Political Science	304	304	304	304
Psychology	155	155	155	155
Sociology	220	220	219	220

Supplementary Table 25: Attitudes, Behavior and Norms, Number of Responses Across Both Wave Completers

Discipline	Attitude	Awareness	Behavior	Overall
**All Respondents**				
All	1609	1609	1609	1609
Economics	382	382	382	382
Political Science	503	503	503	503
Psychology	344	344	344	344
Sociology	380	380	380	380
**PhD Students**				
All	957	957	957	957
Economics	218	218	218	218
Political Science	256	256	256	256
Psychology	246	246	246	246
Sociology	237	237	237	237
**Published Authors**				
All	652	652	652	652
Economics	164	164	164	164
Political Science	247	247	247	247
Psychology	98	98	98	98
Sociology	143	143	143	143

SI 2.1 Adoption

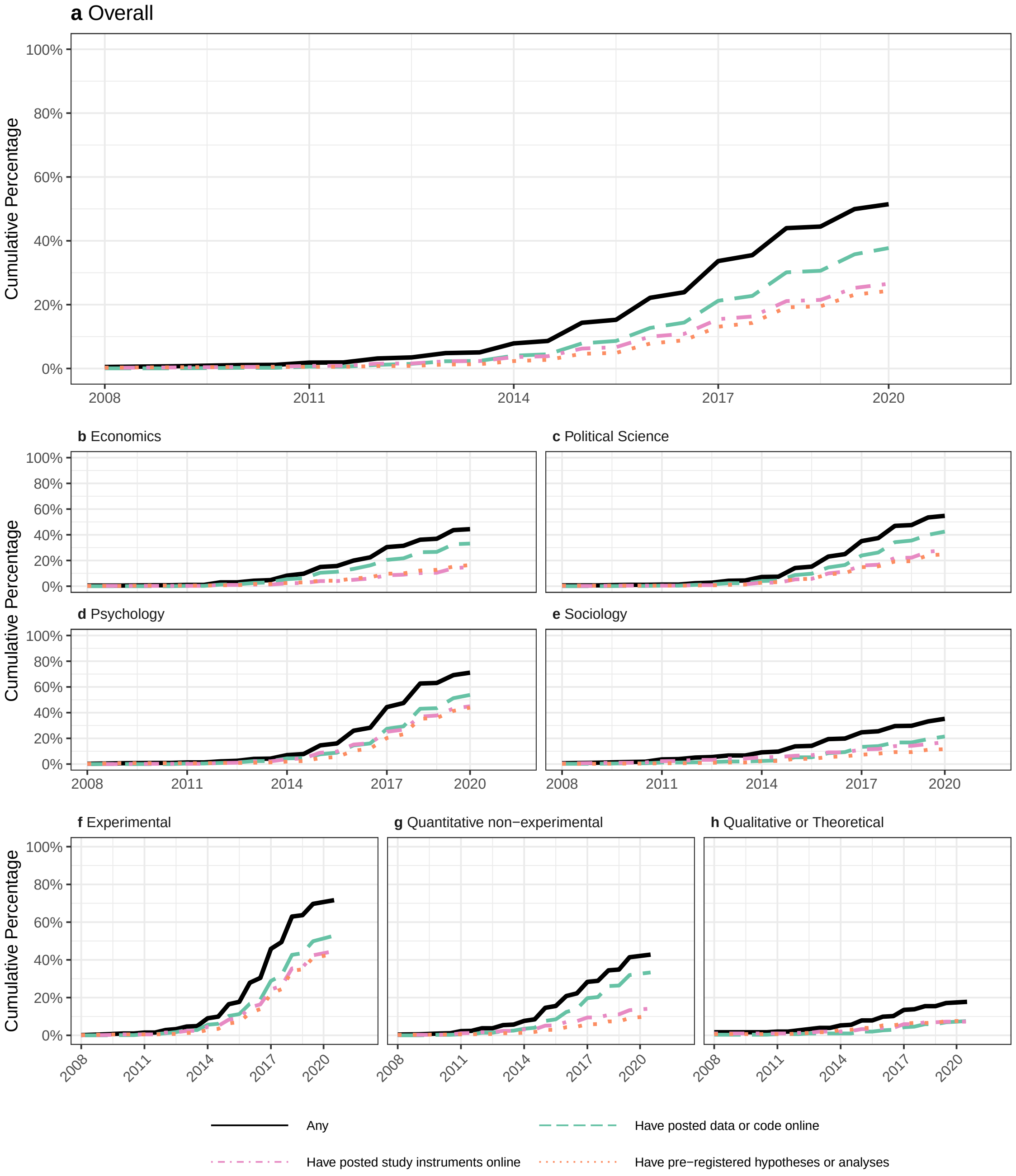

Supplementary Figure 8: **Reported Year of Adoption of Open Science Practices - Students** The figure shows for a given year the cumulative proportion of Phd Students that completed both survey waves who had first completed an open science practice in that year or previously. If a published author responded to both waves of the survey, then their first wave response is prioritized. Posting study instruments online is the response to the question "Approximately when was the first time you publicly posted study instruments online?" Posting data or code online is the response to the question "Approximately when was the first time you publicly posted data or code online?" Pre-registering hypotheses or analyses is the response to the question "Approximately when was the first time you pre-registered hypotheses or analyses in advance of a study?" Panel a shows data from all student respondents, and Panels b-e show responses from students identified as economists, political scientists, psychologists, and sociologists, respectively. Panels f-h show responses from students who self-reported primarily being experimentalists, quantitative non-experimental, and qualitative or theoretical, respectively.

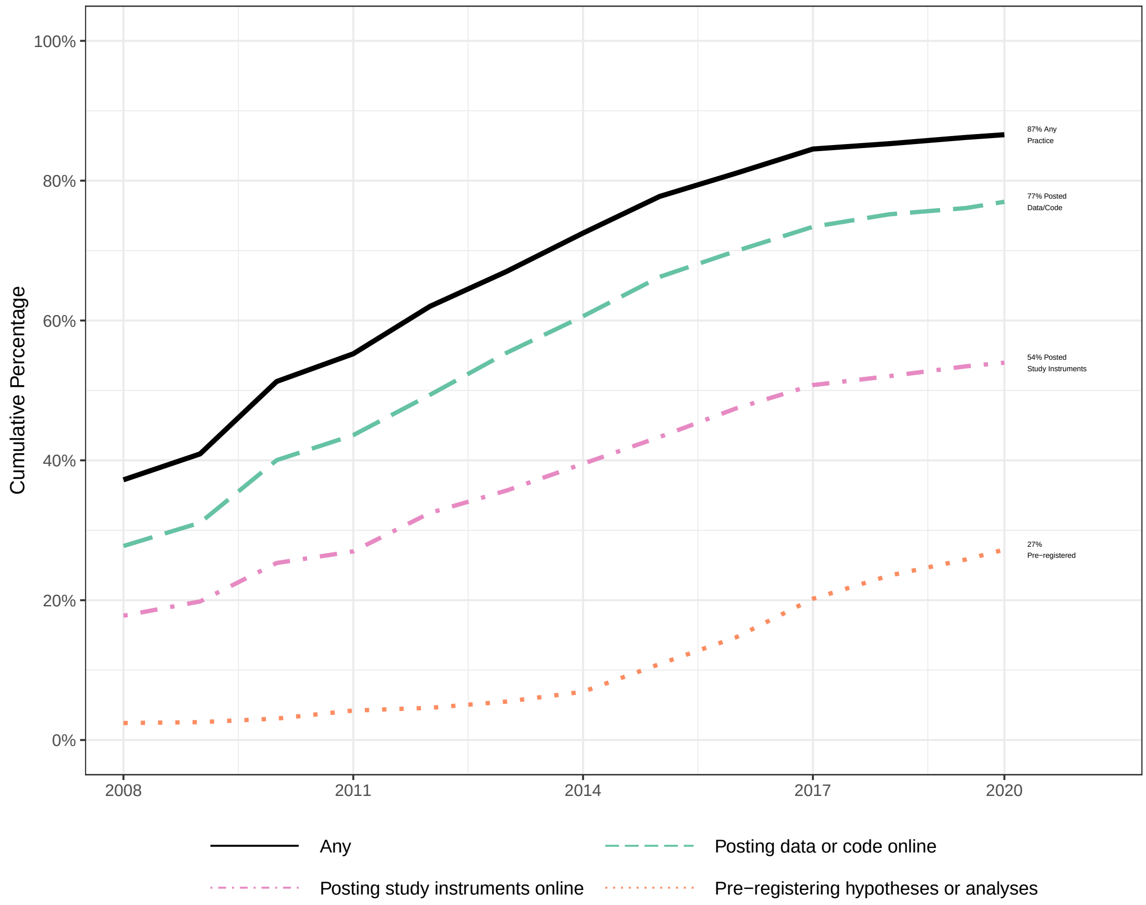

Supplementary Figure 9: **Reported Year of Adoption of Open Science Practices - Prioritize Wave 1 response** The figure shows for a given year the cumulative proportion of published authors that completed both survey waves who had first completed an open science practice in that year or previously. If a published author responded to both waves of the survey, then their first wave response is prioritized. Posting study instruments online is the response to the question "Approximately when was the first time you publicly posted study instruments online?" Posting data or code online is the response to the question "Approximately when was the first time you publicly posted data or code online?" Pre-registering hypotheses or analyses is the response to the question "Approximately when was the first time you pre-registered hypotheses or analyses in advance of a study?" The sample is restricted to published authors who completed their PhD by 2009

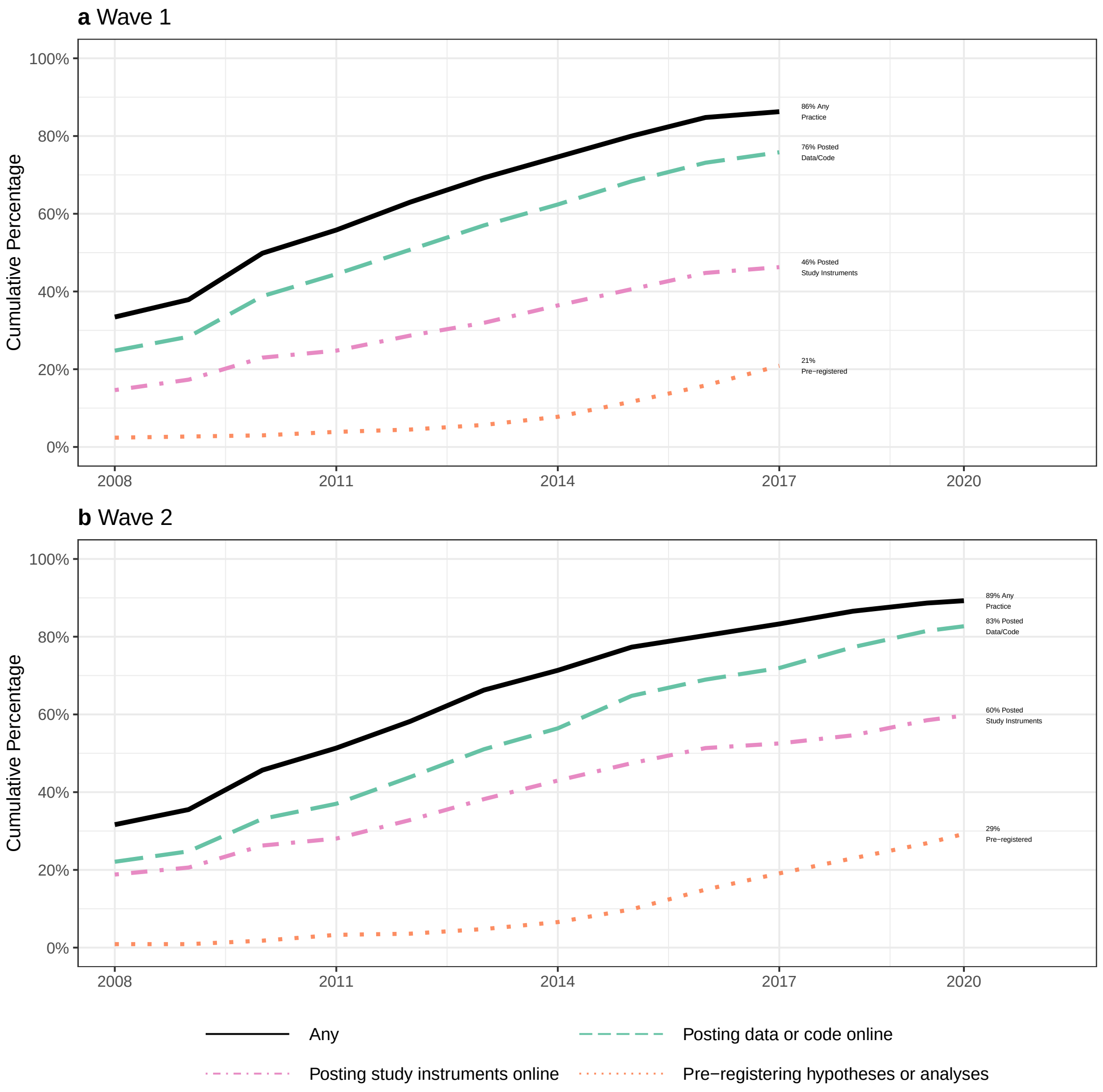

Supplementary Figure 10: **Reported Year of Adoption of Open Science Practices - Both Wave Completers** The figure shows for a given year the cumulative proportion of published authors that completed both survey waves who had first completed an open science practice in that year or previously. Panel a shows the first wave responses and panel b the second wave responses. Posting study instruments online is the response to the question "Approximately when was the first time you publicly posted study instruments online?" Posting data or code online is the response to the question "Approximately when was the first time you publicly posted data or code online?" Pre-registering hypotheses or analyses is the response to the question "Approximately when was the first time you pre-registered hypotheses or analyses in advance of a study?" The sample is restricted to published authors who completed their PhD by 2009 and completed both waves of the survey.

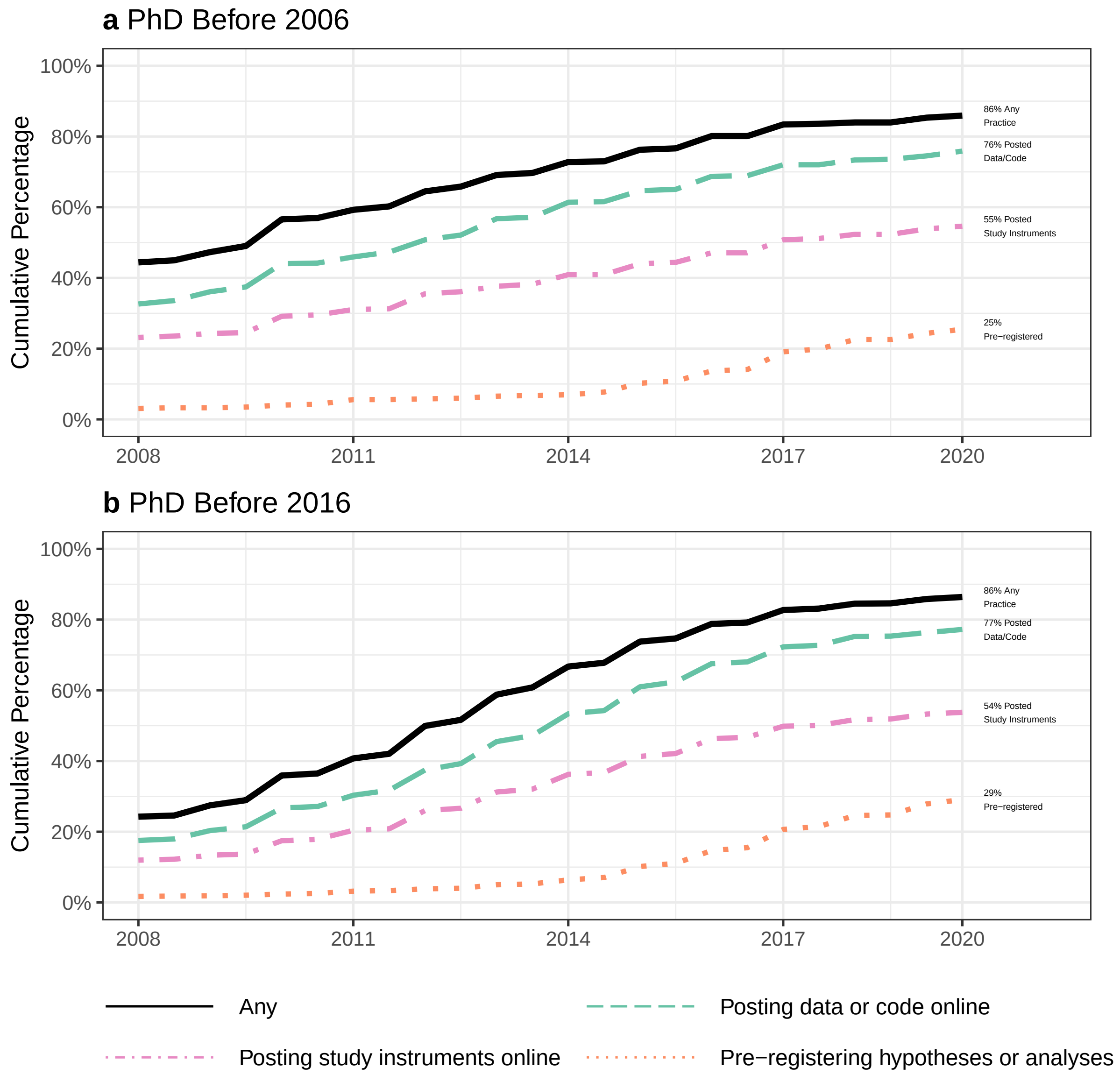

Supplementary Figure 11: **Reported Year of Adoption of Open Science Practices - Alternate Cutoff Dates.** The chart shows for a given year the cumulative proportion of published authors who had first completed an open science practice in that year or previously. Posting study instruments online is the response to the question "Approximately when was the first time you publicly posted study instruments online?" Posting data or code online is the response to the question "Approximately when was the first time you publicly posted data or code online?" Pre-registering hypotheses or analyses is the response to the question "Approximately when was the first time you pre-registered hypotheses or analyses in advance of a study?" The sample is restricted to published authors who completed their PhD by 2006 in Panel a, and published authors who completed their PhD prior to 2016 in Panel b.

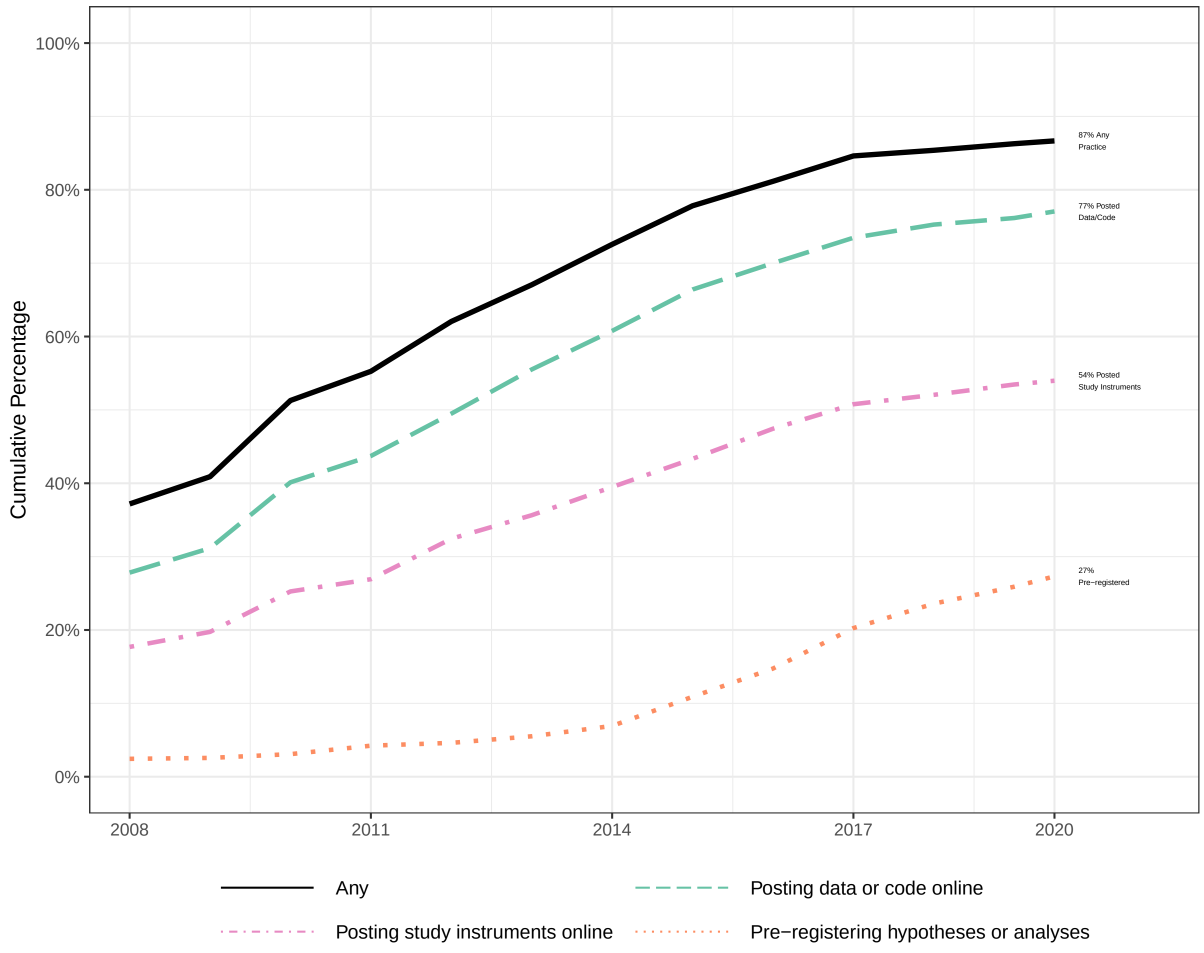

Supplementary Figure 12: **Reported Year of Adoption of Open Science Practices - Robustness to Dropping Logically Inconsistent Participants.** The figure shows for a given year the cumulative proportion of published authors who had first completed an open science practice in that year or previously. If a published author responded to both waves of the survey, and answered the survey in a logically inconsistent manner, then their response is dropped (N=12). Posting study instruments online is the response to the question "Approximately when was the first time you publicly posted study instruments online?" Posting data or code online is the response to the question "Approximately when was the first time you publicly posted data or code online?" Pre-registering hypotheses or analyses is the response to the question "Approximately when was the first time you pre-registered hypotheses or analyses in advance of a study?" The sample is restricted to published authors who completed their PhD by 2009.

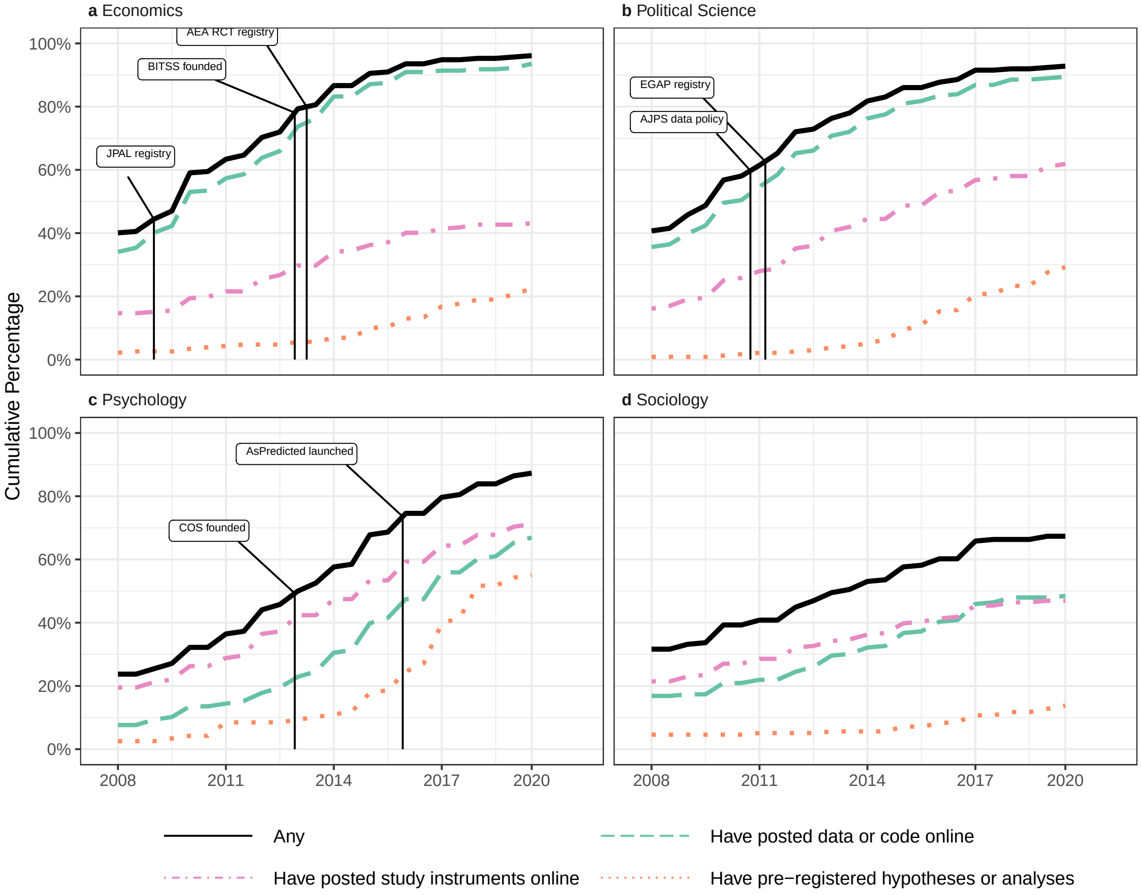

Supplementary Figure 13: **Reported Year of Adoption of Open Science Practices** The figure shows for a given year the cumulative proportion of published authors that completed both survey waves who had first completed an open science practice in that year or previously. Posting study instruments online is the response to the question "Approximately when was the first time you publicly posted study instruments online?" Posting data or code online is the response to the question "Approximately when was the first time you publicly posted data or code online?" Pre-registering hypotheses or analyses is the response to the question "Approximately when was the first time you pre-registered hypotheses or analyses in advance of a study?" The sample is restricted to published authors who completed their PhD by 2009. The organizations mentioned in the figure are included in the panel of the discipline that they work in. BITSS and COS are interdisciplinary organizations, but are included with the discipline they are most associated with. Panels a-d show responses from published authors identified as economists, political scientists, psychologists, and sociologists, respectively.

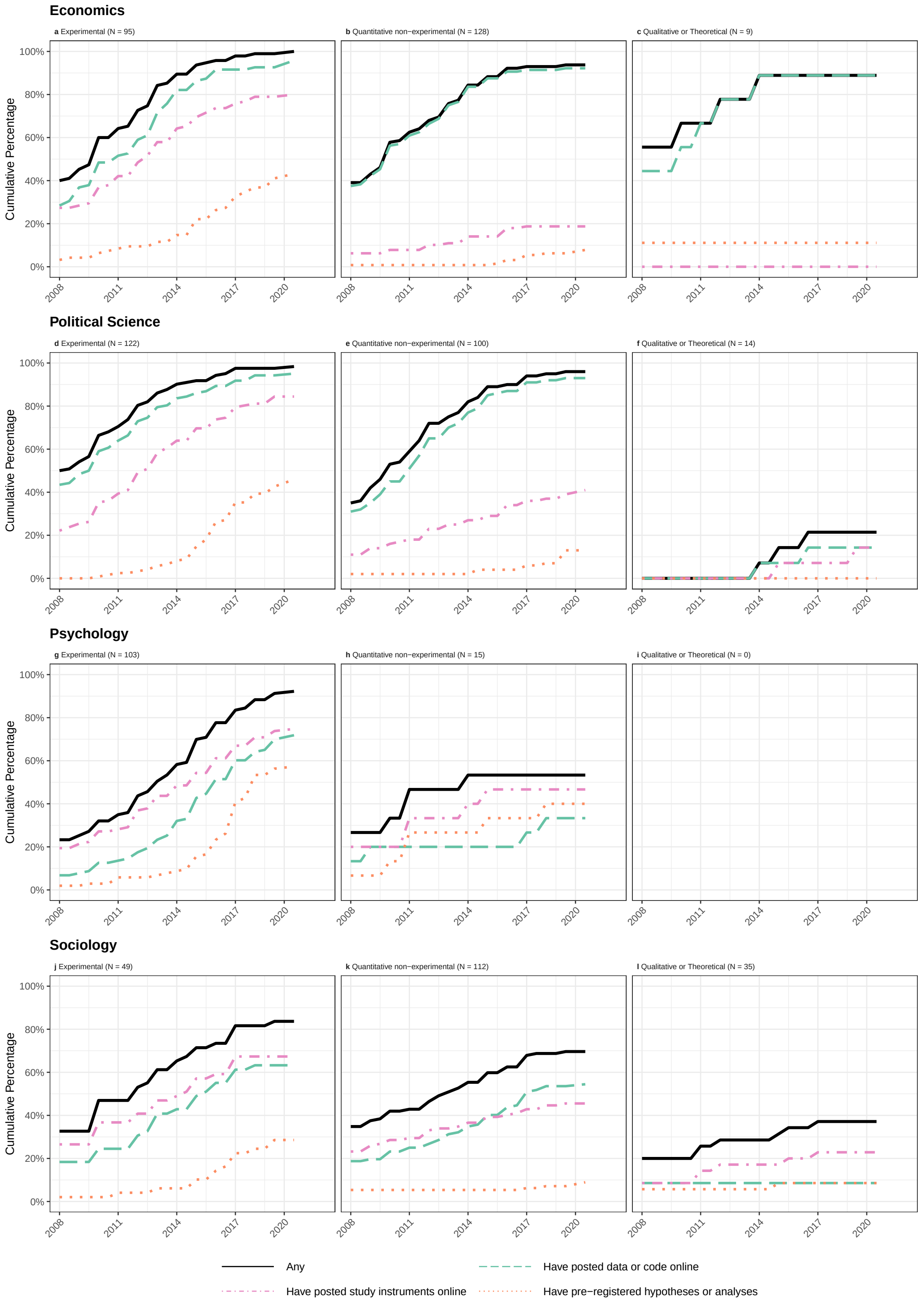

Supplementary Figure 14: **Reported Year of Adoption of Open Science Practices - By Discipline and by Research Type** The figure shows for a given year the cumulative proportion of published authors that completed both survey waves who had first completed an open science practice in that year or previously split by Discipline and Research type. Posting study instruments online is the response to the question "Approximately when was the first time you publicly posted study instruments online?" Posting data or code online is the response to the question "Approximately when was the first time you publicly posted data or code online?" Pre-registering hypotheses or analyses is the response to the question "Approximately when was the first time you pre-registered hypotheses or analyses in advance of a study?" The sample is restricted to published authors who completed their PhD by 2009 (N = 782). Panels a-c show data from published authors identified as economists, who self-reported primarily being experimentalists, quantitative non-experimental, and qualitative or theoretical, respectively. Panels d-f, g-i, and j-l, show the equivalent data for political scientists, psychologists, and sociologists, respectively.

SI 2.1.1 Adoption Heterogeneity

Supplementary Table 26: Differences in Adoption across Disciplines

	Posting data or code online		Posting study instruments		Pre-registering hypotheses or analyses	
	(1)	(2)	(3)	(4)	(5)	(6)
Economics	0.184***	0.172***	0.023**	0.026*	0.076***	0.083***
	(0.01)	(0.01)	(0.01)	(0.01)	(0.01)	(0.01)
Political Science	0.173***	0.169***	0.086***	0.092***	0.081***	0.080***
	(0.01)	(0.01)	(0.01)	(0.01)	(0.01)	(0.01)
Psychology	0.080***	0.098***	0.101***	0.124***	0.161***	0.160***
	(0.01)	(0.01)	(0.01)	(0.01)	(0.01)	(0.01)
Years since started PhD		0.000		0.000		-0.001
		(0)		(0)		(0)
Male		0.044***		0.035***		0.011
		(0.01)		(0.01)		(0.01)
Tenured		0.039***		0.068***		-0.006
		(0.01)		(0.02)		(0.01)
Leadership Position		0.002		0.004		-0.009
		(0.01)		(0.01)		(0.01)
USA and Canada		-0.010		-0.067***		0.007
		(0.01)		(0.02)		(0.01)
Constant	0.458***	0.441***	0.614***	0.617***	0.322***	0.319***
	(0)	(0.01)	(0.01)	(0.02)	(0)	(0.01)
Observations	3,179	1,981	3,179	1,981	3,179	1,981

This table presents regression coefficients and standard errors from ordinary least squares regressions. The outcome variable in each regression is one of the sub indices described in Supplementary Table 7. The covariates are indicator variables for the discipline of the respondent. In odd numbered specifications no other control variables are included. In even numbered specifications individual-level covariates are included. The omitted discipline in the regressions is Sociology. Significance stars are indicated for standard errors computed using a multiple testing adjustment to address risk of false positives. In particular, we use the False Discovery Rate (FDR) adjustment in [11] and discussed in [12]. * indicates significance at the 10% level, ** indicates significance at the 5% level and *** indicates significance at the 1% level two-sided t-test.

Supplementary Table 27: Differences in Adoption by Author Type

	Posting data or code online		Posting study instruments		Pre-registering hypotheses or analyses	
	(1)	(2)	(3)	(4)	(5)	(6)
Published Author	0.057***	0.034***	0.094***	0.084***	-0.017***	0.006
	(0.01)	(0.01)	(0.01)	(0.02)	(0.01)	(0.01)
Years since started PhD		-0.002***		-0.002*		-0.001*
		(0)		(0)		(0)
Male		0.072***		0.023**		0.004
		(0.01)		(0.01)		(0.01)
Tenured		0.046***		0.034*		-0.014
		(0.01)		(0.02)		(0.01)
Leadership Position		-0.003		0.009		-0.008
		(0.01)		(0.01)		(0.01)
USA and Canada		-0.006		-0.048**		0.001
		(0.01)		(0.02)		(0.01)
Constant	0.542***	0.538***	0.626***	0.665***	0.405***	0.413***
	(0)	(0.01)	(0.01)	(0.02)	(0)	(0.01)
Observations	3,179	1,981	3,179	1,981	3,179	1,981

This table presents regression coefficients and standard errors from ordinary least squares regressions. The outcome variable in each regression is one of the sub indices described in Supplementary Table 7. The covariates are indicator variables for the whether the respondent has published in one of the journals in Supplementary Tables 2 through 5. In odd numbered specifications no other control variables are included. In even numbered specifications individual-level covariates are included. The omitted career stage in the regressions is PhD students. Significance stars are indicated for standard errors computed using a multiple testing adjustment to address risk of false positives. In particular, we use the False Discovery Rate (FDR) adjustment in [11] and discussed in [12]. * indicates significance at the 10% level, ** indicates significance at the 5% level and *** indicates significance at the 1% level two-sided t-test.

Supplementary Table 28: Differences in Adoption across Disciplines and Author type

	Posting data or code online		Posting study instruments		Pre-registering hypotheses or analyses	
	(1)	(2)	(3)	(4)	(5)	(6)
Published Author	0.025***	0.015	0.103***	0.101***	-0.014	-0.006
	(0.01)	(0.01)	(0.01)	(0.02)	(0.01)	(0.01)
Economics	0.163***	0.157***	0.021	0.037*	0.078***	0.08***
	(0.01)	(0.01)	(0.01)	(0.02)	(0.01)	(0.01)
Political Science	0.141***	0.144***	0.065***	0.086***	0.084***	0.079***
	(0.01)	(0.01)	(0.01)	(0.02)	(0.01)	(0.01)
Psychology	0.094***	0.107***	0.127***	0.153***	0.153***	0.152***
	(0.01)	(0.01)	(0.01)	(0.02)	(0.01)	(0.01)
Published Author:Economics	0.043***	0.036**	-0.007	-0.022	-0.002	0.008
	(0.01)	(0.02)	(0.02)	(0.03)	(0.01)	(0.02)
Published Author:Political Science	0.06***	0.051***	0.023	0.004	-0.005	0.003
	(0.01)	(0.01)	(0.02)	(0.03)	(0.01)	(0.02)
Published Author:Psychology	-0.037***	-0.035**	-0.056**	-0.088***	0.019	0.023
	(0.01)	(0.02)	(0.02)	(0.03)	(0.02)	(0.02)
Years since started PhD		-0.001		-0.001		-0.001
		(0)		(0)		(0)
Male		0.042***		0.032***		0.011
		(0.01)		(0.01)		(0.01)
Tenured		0.021*		0.032		-0.004
		(0.01)		(0.02)		(0.01)
Leadership Position		0.002		0.007		-0.008
		(0.01)		(0.01)		(0.01)
USA and Canada		-0.003		-0.047**		0.008
		(0.01)		(0.02)		(0.01)
Constant	0.447***	0.44***	0.572***	0.586***	0.327***	0.321***
	(0)	(0.01)	(0.01)	(0.02)	(0.01)	(0.01)
Observations	3,179	1,981	3,179	1,981	3,179	1,981

This table presents regression coefficients and standard errors from ordinary least squares regressions. The outcome variable in each regression is one of the sub indices described in Supplementary Table 7. The covariates are indicator variables for the whether the respondent has published in one of the journals in Supplementary Tables 2 through 5. In odd numbered specifications no other control variables are included. In even numbered specifications individual-level covariates are included. The omitted discipline in the regressions is Sociology, and the omitted career stage is PhD students. Significance stars are indicated for standard errors computed using a multiple testing adjustment to address risk of false positives. In particular, we use the False Discovery Rate (FDR) adjustment in [11] and discussed in [12]. * indicates significance at the 10% level, ** indicates significance at the 5% level and *** indicates significance at the 1% level from a two-sided t-test.

SI 2.1.2 Relationship between Past and Current Open Science Behavior

Supplementary Table 29: Relationship between Past and Current Open Science Behavior

	Used in Last Paper:			
	Any practice	Posting data or code online	Posting study instruments	Pre-registering hypotheses or analyses
	(1)	(2)	(3)	(4)
Has done any practice ever	0.74*** (0.03)			
Has done posting data or code online		0.68*** (0.02)		
Has done posting study instruments			0.61*** (0.02)	
Has done pre-registering hypotheses or analyses				0.55*** (0.02)
Constant	0.004 (0.03)	0.01 (0.02)	0.003 (0.01)	0.002 (0.01)
Observations	1,420	1,420	1,420	1,420

This table presents regression coefficients and standard errors from ordinary least squares regressions. The outcome variable in each regression is an indicator variable for whether the individual conducted an open science behavior in their last paper. The covariates are indicator variables for whether the individual had ever undertaken such an open science practice. The sample is limited to published authors. * indicates significance at the 10% level, ** indicates significance at the 5% level and *** indicates significance at the 1% level from a two-sided t-test. There is no adjustment for multiple testing.

Supplementary Table 30: Relationship between Past and Current Open Science Behavior, by Quartiles

	Used in Last Paper:			
	Any practice	Posting data or code online	Posting study instruments	Pre-registering hypotheses or analyses
	(1)	(2)	(3)	(4)
First Practice Q1 (1984 - 2008)	0.75*** (0.03)			
First Practice Q2 (2008 - 2012)	0.77*** (0.03)			
First Practice Q3 (2012 - 2015)	0.77*** (0.03)			
First Practice Q4 (2015 - 2020)	0.65*** (0.03)			
Post Data/Code Q1 (1984 - 2010)		0.74*** (0.03)		
Post Data/Code Q2 (2010 - 2013)		0.76*** (0.03)		
Post Data/Code Q3 (2013 - 2016)		0.64*** (0.03)		
Post Data/Code Q4 (2016 - 2020)		0.57*** (0.03)		
Post Study Instruments Q1 (1996 - 2010)			0.64*** (0.04)	
Post Study Instruments Q2 (2010 - 2013)			0.58*** (0.04)	
Post Study Instruments Q3 (2013 - 2016)			0.55*** (0.04)	
Post Study Instruments Q4 (2016 - 2020)			0.54*** (0.04)	
Pre-registration Q1 (1996 - 2015)				0.61*** (0.05)
Pre-registration Q2 (2015 - 2016)				0.57*** (0.05)
Pre-registration Q3 (2016 - 2018)				0.49*** (0.05)
Pre-registration Q4 (2018 - 2020)				0.44*** (0.05)
Observations	1,186	1,056	739	406

This table presents regression coefficients and standard errors from ordinary least squares regressions. The outcome variable in each regression is an indicator variable for whether the individual conducted an open science behavior in their last paper. The covariates are indicator variables for the quartile when the individual first undertook such an open science practice. The sample is limited to published authors. * indicates significance at the 10% level, ** indicates significance at the 5% level and *** indicates significance at the 1% level from a two-sided t-test. There is no adjustment for multiple testing.

SI 2.2 Attitudes, Behavior and Norms

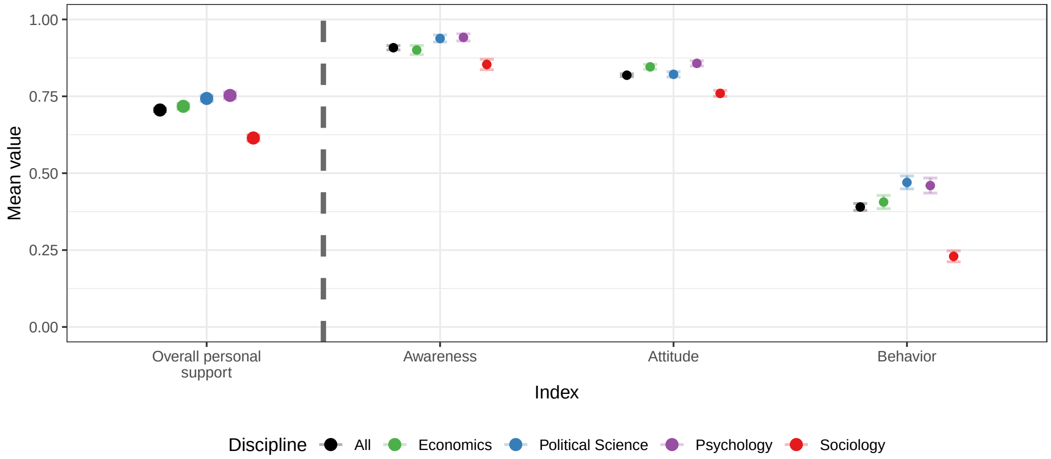

Supplementary Figure 15: **Open Science Awareness, Attitudes and Behavior - by Discipline.** Dots represent means and lines around the dots are 95% confidence intervals for the estimates. Awareness is an index comprised of questions related to the respondent's i) Awareness of posting data or code online, ii) Awareness of posting study instruments and iii) Awareness of pre-registration. Behavior is an index comprised of questions related to the respondent's i) Behavior of posting data or code online, ii) Behavior of posting study instruments and iii) Behavior of pre-registration. Attitudes is an index comprised of questions related to the respondent's i) Attitudes of posting data or code online, ii) Attitudes of posting study instruments and iii) Attitudes of pre-registration. Overall Personal Support is an average of the three indices. The questions and methodology that are used to construct the indices can be found in Supplementary Table. 7. The sample is comprised of those who responded to at least one wave of surveys. If an individual responded to both waves their second survey response is prioritized. See Supplementary Table 22 for the N for each data point.

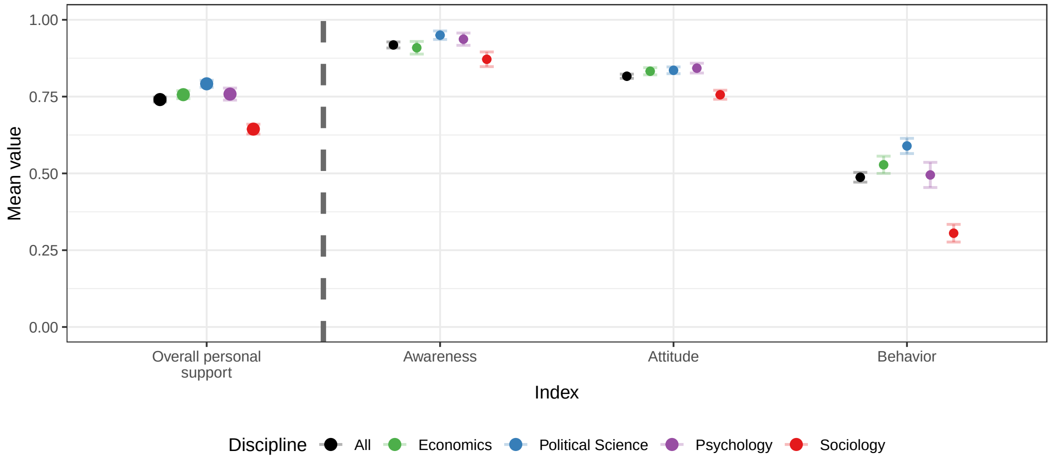

Supplementary Figure 16: **Published Author Open Science Awareness, Attitudes and Behavior - By Discipline.** Dots represent means and lines around the dots are 95% confidence intervals for the estimates. Sample is restricted to published authors. Awareness is an index comprised of questions related to the respondent's i) Awareness of posting data or code online, ii) Awareness of posting study instruments and iii) Awareness of pre-registration. Behavior is an index comprised of questions related to the respondent's i) Behavior of posting data or code online, ii) Behavior of posting study instruments and iii) Behavior of pre-registration. Attitudes is an index comprised of questions related to the respondent's i) Attitudes of posting data or code online, ii) Attitudes of posting study instruments and iii) Attitudes of pre-registration. Overall Personal Support is an average of the three indices. The questions and methodology that are used to construct the indices can be found in Supplementary Table. 7. The sample is comprised of those who responded to at least one wave of surveys. If an individual responded to both waves their second survey response is prioritized. See Supplementary Table 22 for the N for each data point.

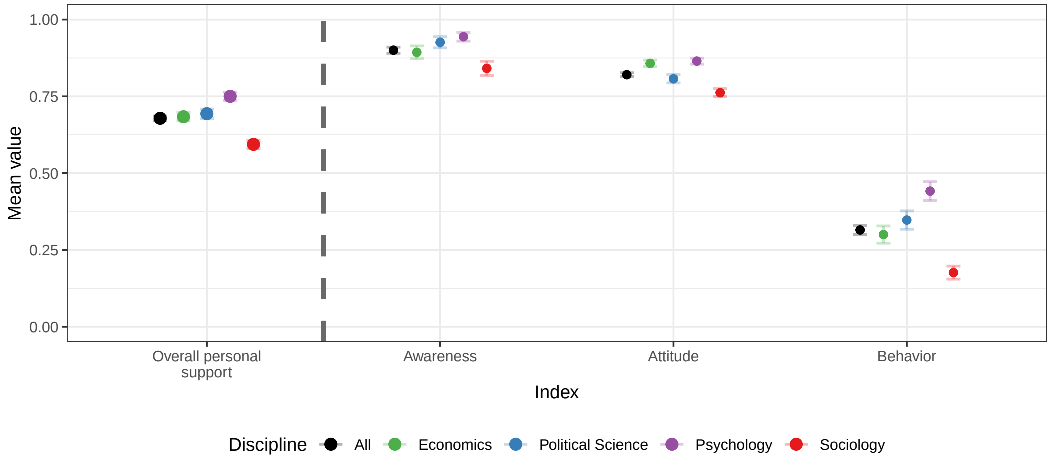

Supplementary Figure 17: **Student Open Science Awareness, Attitudes and Behavior - By Discipline.** Dots represent means and lines around the dots are 95% confidence intervals for the estimates. Sample is restricted to PhD students. Awareness is an index comprised of questions related to the respondent's i) Awareness of posting data or code online, ii) Awareness of posting study instruments and iii) Awareness of pre-registration. Behavior is an index comprised of questions related to the respondent's i) Behavior of posting data or code online, ii) Behavior of posting study instruments and iii) Behavior of pre-registration. Attitudes is an index comprised of questions related to the respondent's i) Attitudes of posting data or code online, ii) Attitudes of posting study instruments and iii) Attitudes of pre-registration. Overall Personal Support is an average of the three indices. The questions and methodology that are used to construct the indices can be found in Supplementary Table 7. The sample is comprised of those who responded to at least one wave of surveys. If an individual responded to both waves their second survey response is prioritized. See Supplementary Table 22 for the N for each data point.

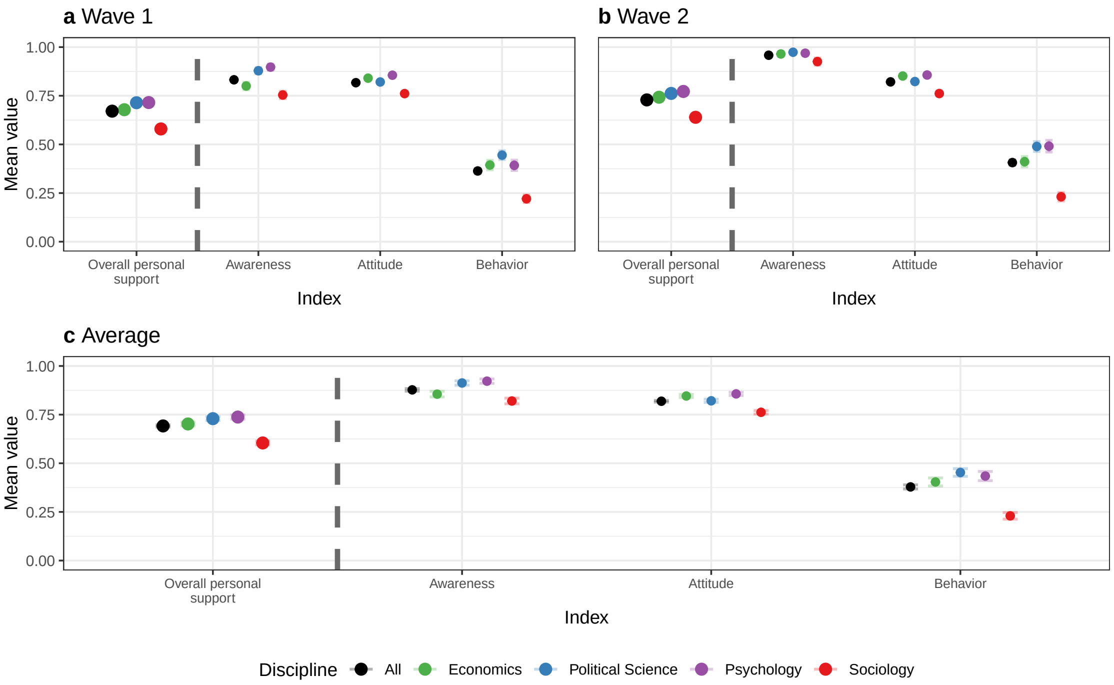

Supplementary Figure 18: **Open Science Awareness, Attitudes and Behavior - By Discipline.** Dots represent means and lines around the dots are 95% confidence intervals for the estimates. Awareness is an index comprised of questions related to the respondent's i) Awareness of posting data or code online, ii) Awareness of posting study instruments and iii) Awareness of pre-registration. In wave 2, Awareness is automatically coded as 1 for those who completed the first wave. Behavior is an index comprised of questions related to the respondent's i) Behavior of posting data or code online, ii) Behavior of posting study instruments and iii) Behavior of pre-registration. Attitudes is an index comprised of questions related to the respondent's i) Attitudes of posting data or code online, ii) Attitudes of posting study instruments and iii) Attitudes of pre-registration. Overall Personal Support is an average of the three indices. The questions and methodology that are used to construct the indices can be found in Supplementary Table 7. Panels a and b show the responses from Wave 1 and Wave 2 respectively. Panel c shows the average of the wave 1 and wave 2 responses. See Supplementary Tables 23 and 24 for the N for each data point.

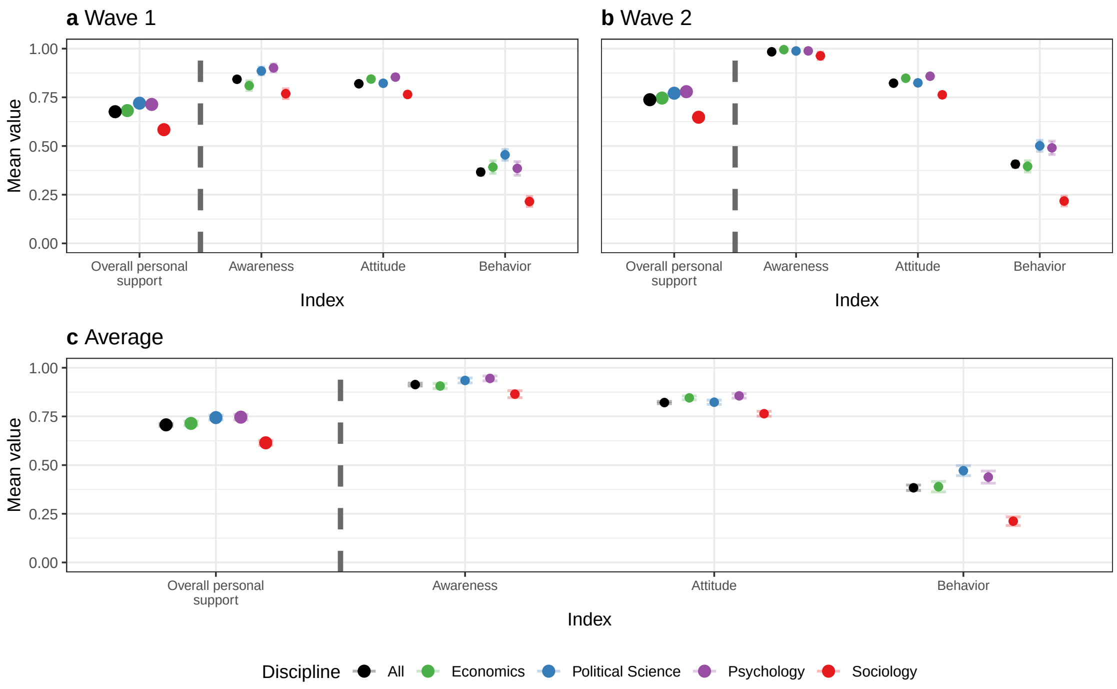

Supplementary Figure 19: **Both Wave Completers - Open Science Awareness, Attitudes and Behavior - By Discipline.** Dots represent means and lines around the dots are 95% confidence intervals for the estimates. Awareness is an index comprised of questions related to the respondent's i) Awareness of posting data or code online, ii) Awareness of posting study instruments and iii) Awareness of pre-registration. In wave 2, Awareness is automatically coded as 1 for those who completed the first wave. Behavior is an index comprised of questions related to the respondent's i) Behavior of posting data or code online, ii) Behavior of posting study instruments and iii) Behavior of pre-registration. Attitudes is an index comprised of questions related to the respondent's i) Attitudes of posting data or code online, ii) Attitudes of posting study instruments and iii) Attitudes of pre-registration. Overall Personal Support is an average of the three indices. The questions and methodology that are used to construct the indices can be found in Supplementary Table 7. Panels a and b show the responses from Wave 1 and Wave 2 respectively. Panel c shows the average of the wave 1 and wave 2 responses. See Supplementary Table 25 for the N for each data point.

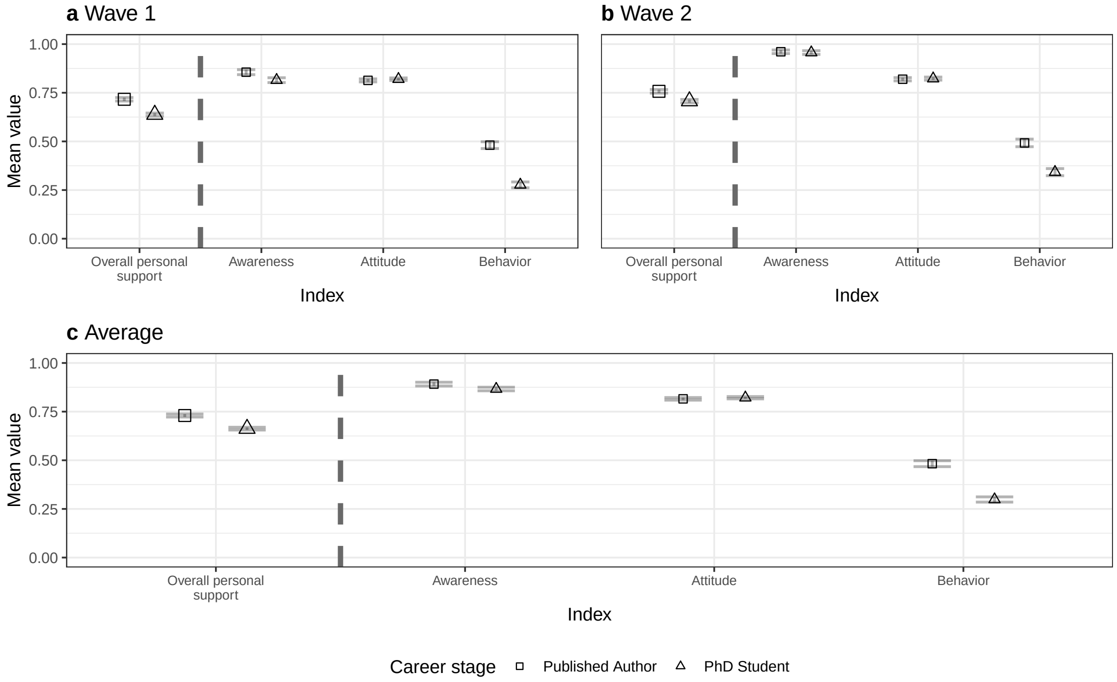

Supplementary Figure 20: **Open Science Awareness, Attitudes and Behavior - By Career Stage.** Dots represent means and lines around the dots are 95% confidence intervals for the estimates. Awareness is an index comprised of questions related to the respondent's i) Awareness of posting data or code online, ii) Awareness of posting study instruments and iii) Awareness of pre-registration. In wave 2, Awareness is automatically coded as 1 for those who completed the first wave. Behavior is an index comprised of questions related to the respondent's i) Behavior of posting data or code online, ii) Behavior of posting study instruments and iii) Behavior of pre-registration. Attitudes is an index comprised of questions related to the respondent's i) Attitudes of posting data or code online, ii) Attitudes of posting study instruments and iii) Attitudes of pre-registration. Overall Personal Support is an average of the three indices. The questions and methodology that are used to construct the indices can be found in Supplementary Table 7. Panels a and b show the responses from Wave 1 and Wave 2 respectively. Panel c shows the average of the wave 1 and wave 2 responses. See Supplementary Tables 23 and 24 for the N for each data point.

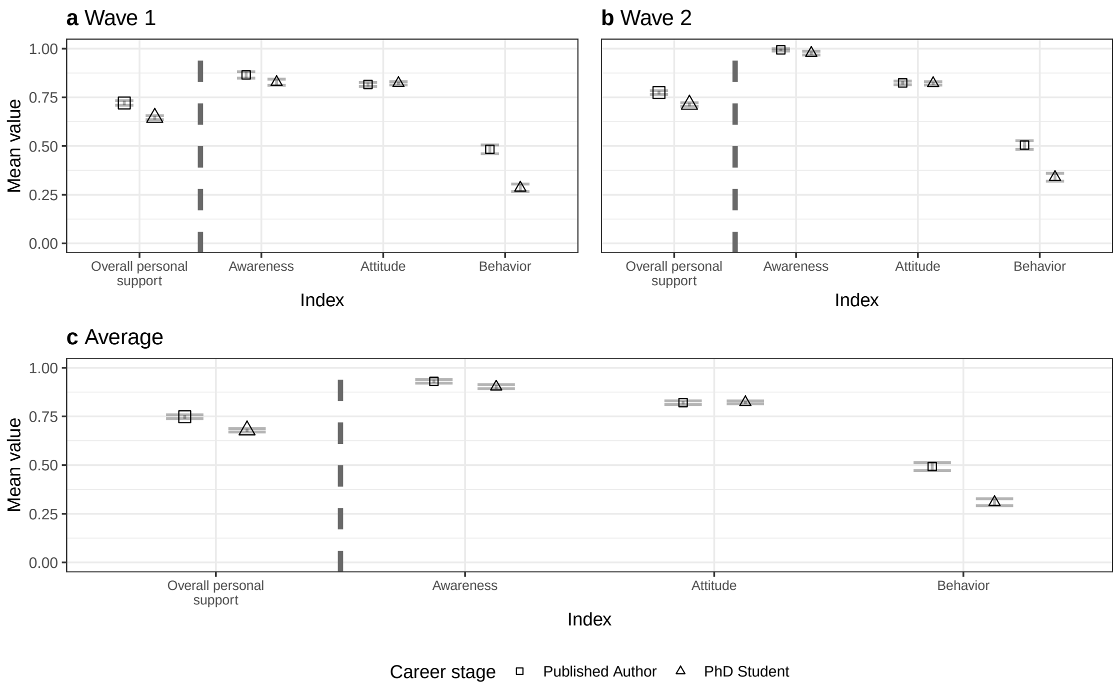

Supplementary Figure 21: **Both Wave Completers Open Science Awareness, Attitudes and Behavior - By Career Stage.** Dots represent means and lines around the dots are 95% confidence intervals for the estimates. Awareness is an index comprised of questions related to the respondent's i) Awareness of posting data or code online, ii) Awareness of posting study instruments and iii) Awareness of pre-registration. In wave 2, Awareness is automatically coded as 1 for those who completed the first wave. Behavior is an index comprised of questions related to the respondent's i) Behavior of posting data or code online, ii) Behavior of posting study instruments and iii) Behavior of pre-registration. Attitudes is an index comprised of questions related to the respondent's i) Attitudes of posting data or code online, ii) Attitudes of posting study instruments and iii) Attitudes of pre-registration. Overall Personal Support is an average of the three indices. The questions and methodology that are used to construct the indices can be found in Supplementary Table 7. Panels a and b show the responses from Wave 1 and Wave 2 respectively. Panel c shows the average of the wave 1 and wave 2 responses. See Supplementary Table 25 for the N for each data point.

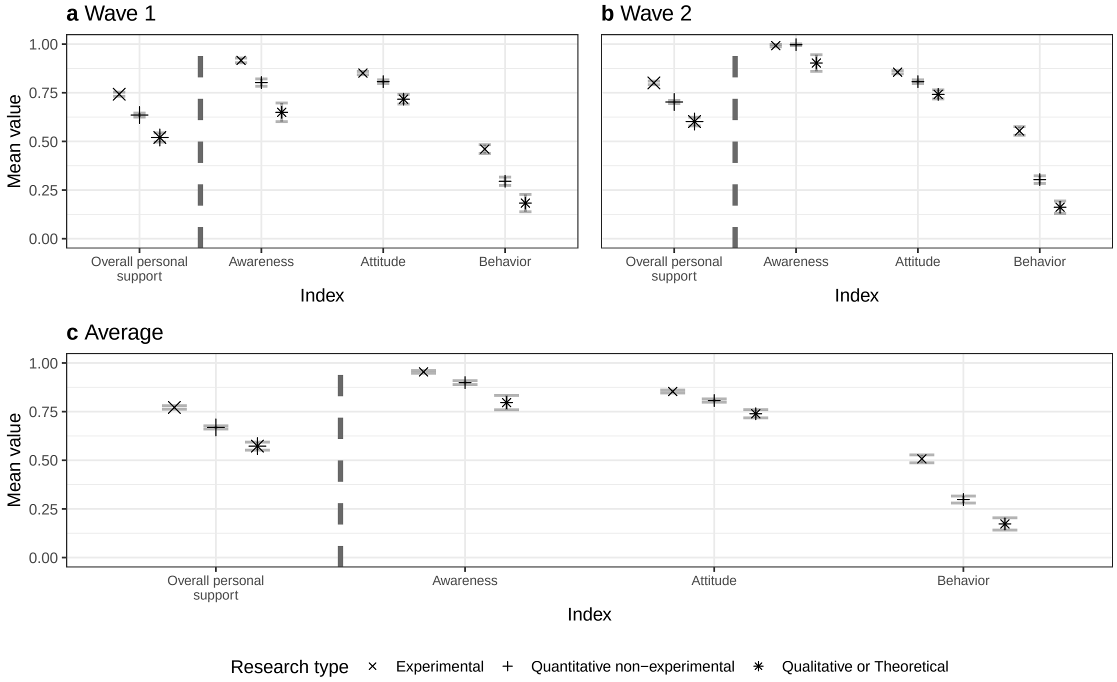

Supplementary Figure 22: **Both Wave Completers Open Science Awareness, Attitudes and Behavior - By Research Type.** Dots represent means and lines around the dots are 95% confidence intervals for the estimates. Awareness is an index comprised of questions related to the respondent's i) Awareness of posting data or code online, ii) Awareness of posting study instruments and iii) Awareness of pre-registration. In wave 2, Awareness is automatically coded as 1 for those who completed the first wave. Behavior is an index comprised of questions related to the respondent's i) Behavior of posting data or code online, ii) Behavior of posting study instruments and iii) Behavior of pre-registration. Attitudes is an index comprised of questions related to the respondent's i) Attitudes of posting data or code online, ii) Attitudes of posting study instruments and iii) Attitudes of pre-registration. Overall Personal Support is an average of the three indices. The questions and methodology that are used to construct the indices can be found in Supplementary Table 7. Panels a and b show the responses from Wave 1 and Wave 2 respectively. Panel c shows the average of the wave 1 and wave 2 responses. See Supplementary Table 25 for the N for each data point.

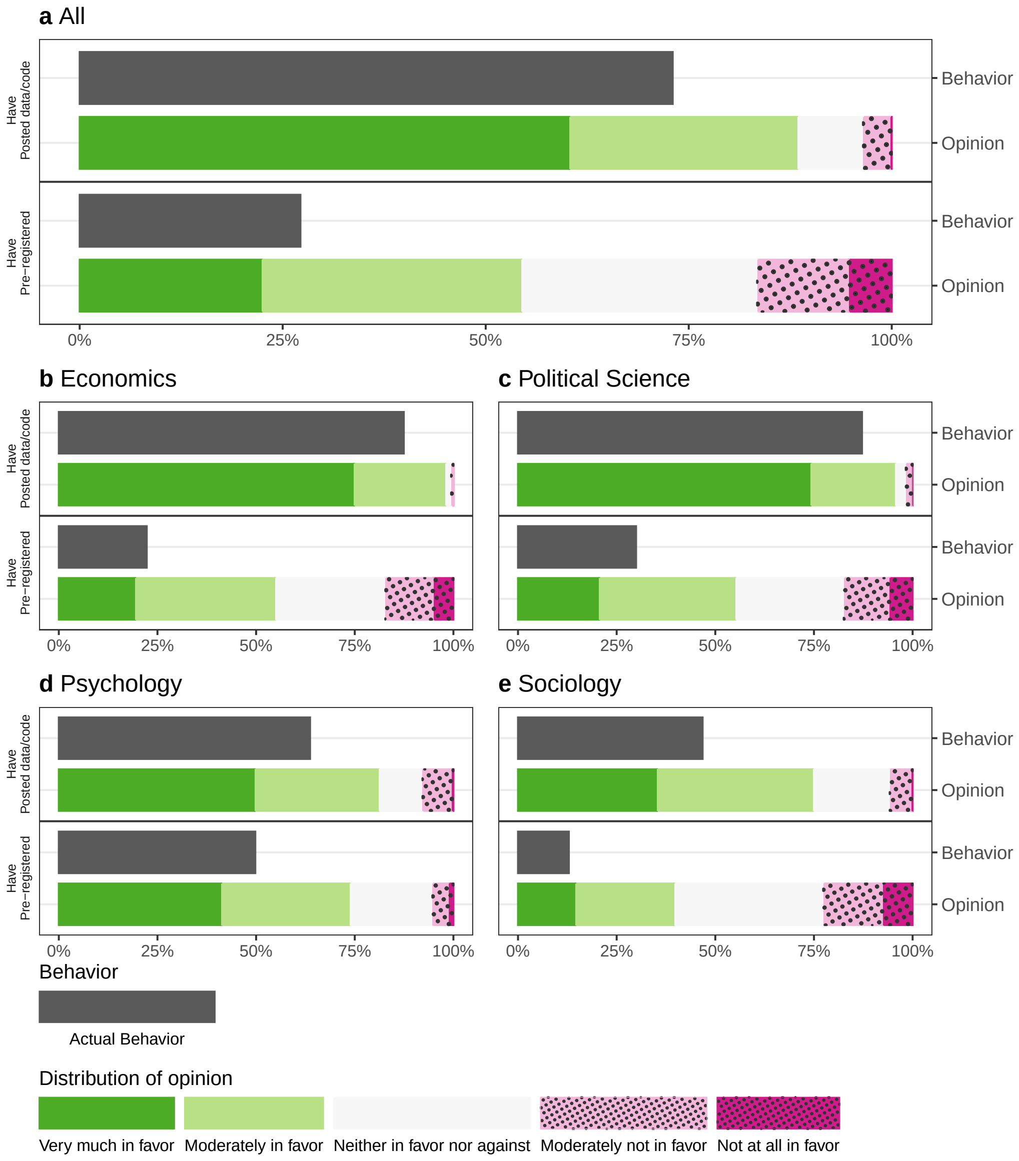

Supplementary Figure 23: **Published Authors Actual Opinion and Behavior** The chart shows support for and engagement with two practices: posting data or code online and pre-registering hypotheses or analyses. The sample is restricted to published authors and is presented overall and by discipline. Within each panel, the solid black bar shows the proportion of published authors who have done the stated practice, using the responses elicited from our survey. Below each solid black bar, the next bar in the panel shows the distribution of support for the practice constructed using the responses elicited from the published authors that we sampled. Colors indicate the level of support, with green indicating more and red indicating less support. Panel a shows data from all published author responses, and Panels b-e show responses from published authors identified as economists, political scientists, psychologists, and sociologists respectively.

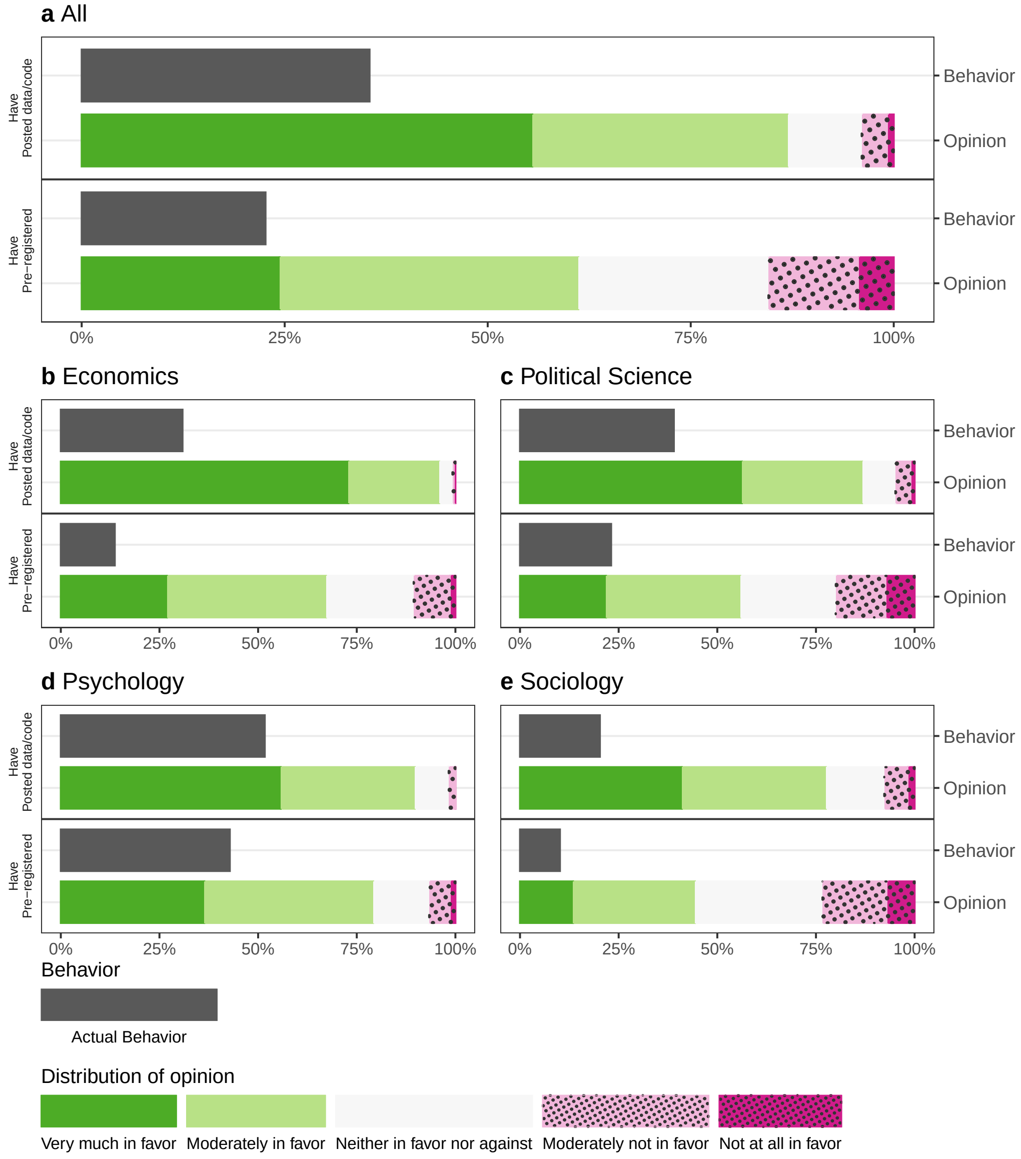

Supplementary Figure 24: **Students Actual Opinion and Behavior** The chart shows support for and engagement with two practices: posting data or code online and pre-registering hypotheses or analyses. The sample is restricted to PhD students and is presented overall and by discipline. Within each panel, the solid black bar shows the proportion of PhD students who have done the stated practice, using the responses elicited from our survey. Below each solid black bar, the next bar in the panel shows the distribution of support for the practice constructed using the responses elicited from the PhD students that we sampled. Colors indicate the level of support, with green indicating more and red indicating less support. Panel a shows data from all student responses, and Panels b-e show responses from students identified as economists, political scientists, psychologists, and sociologists respectively.

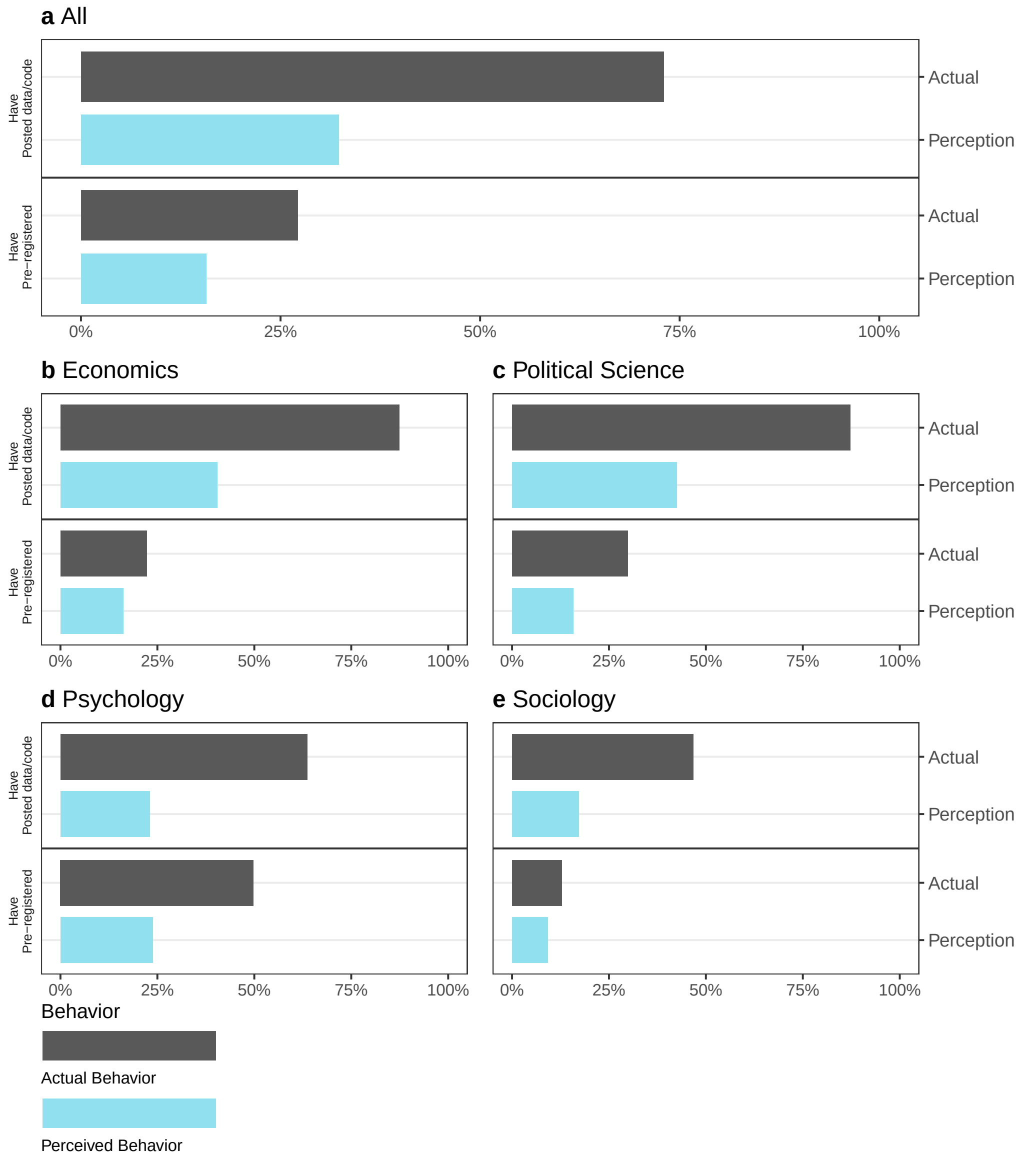

Supplementary Figure 25: **Actual and Perceived Behavior of Open Science Practices among Published Authors.** The chart shows differences between perceived and actual behaviors regarding two practices: posting data or code online and pre-registering hypotheses or analyses. The sample is restricted to published authors; the analogous data for PhD students are presented in Supplementary Fig. 26. Within each panel, the top bar shows the fraction of published authors in a specified discipline that have engaged in an open science practice. The bar underneath shows the average perception among respondents about what proportion of scholars in their discipline have engaged in the open science practice. Panel a shows data from all published author responses, and Panels b-e show responses from published authors identified as economists, political scientists, psychologists, and sociologists respectively.

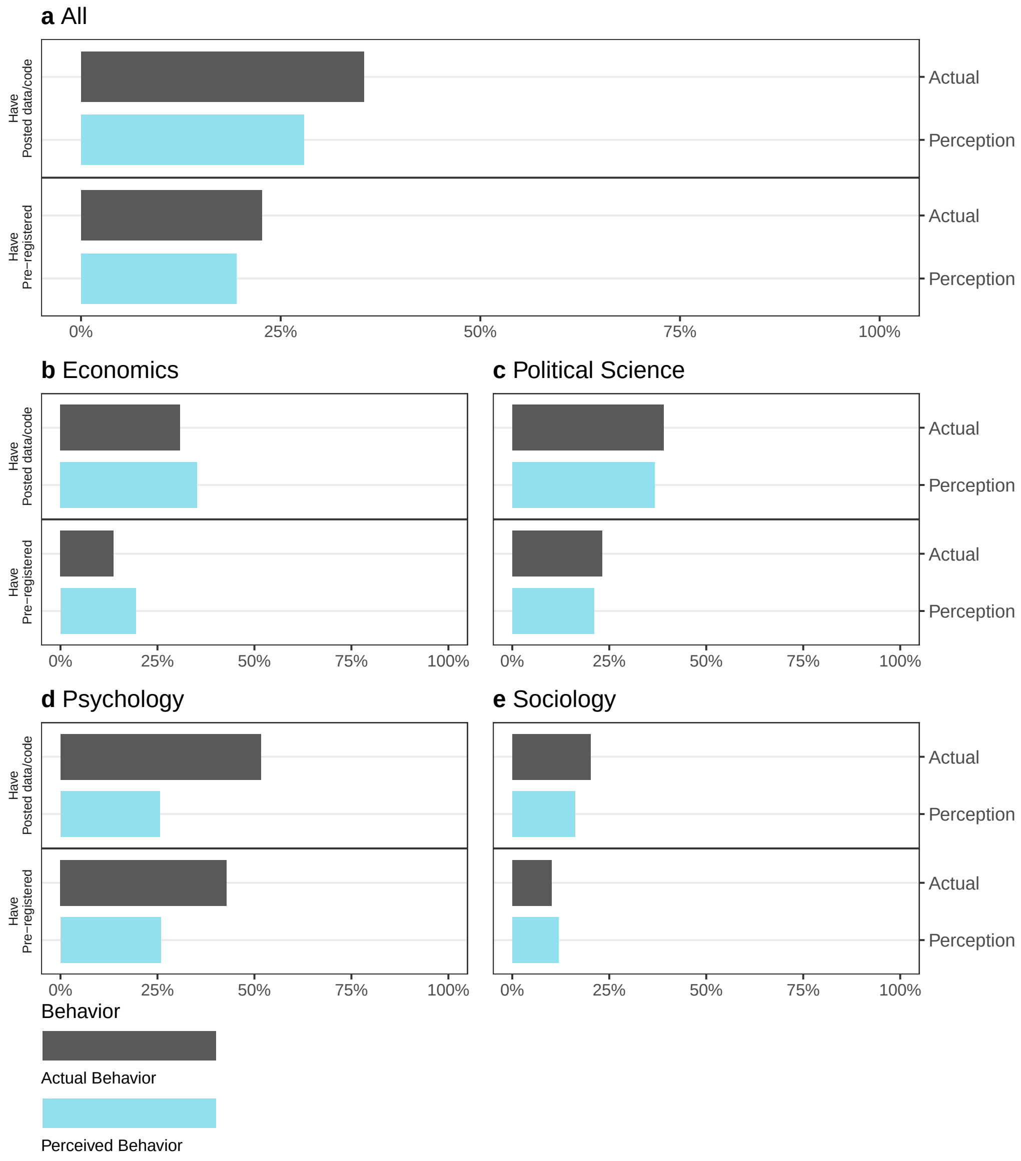

Supplementary Figure 26: **Actual and Perceived Behavior of Open Science Practices among PhD Students** The chart shows differences between perceived and actual usage of two practices: posting data or code online and pre-registering hypotheses or analyses. The sample is restricted to PhD students. Within each panel, the first solid black bar below shows the fraction of Phd Students in a specified discipline that have posted data and/or code. The second light blue bar shows the average proportion of researchers sample respondents believe have posted data/or code, in their discipline. The third solid black bar below shows the fraction of PhD students in a specified discipline that have preregistered a project previously. The final light blue bar shows the average proportion of researchers sample respondents believe have posted pre-registered projects. Panel a shows data from all student responses, and Panels b-e show responses from students identified as economists, political scientists, psychologists, and sociologists respectively.

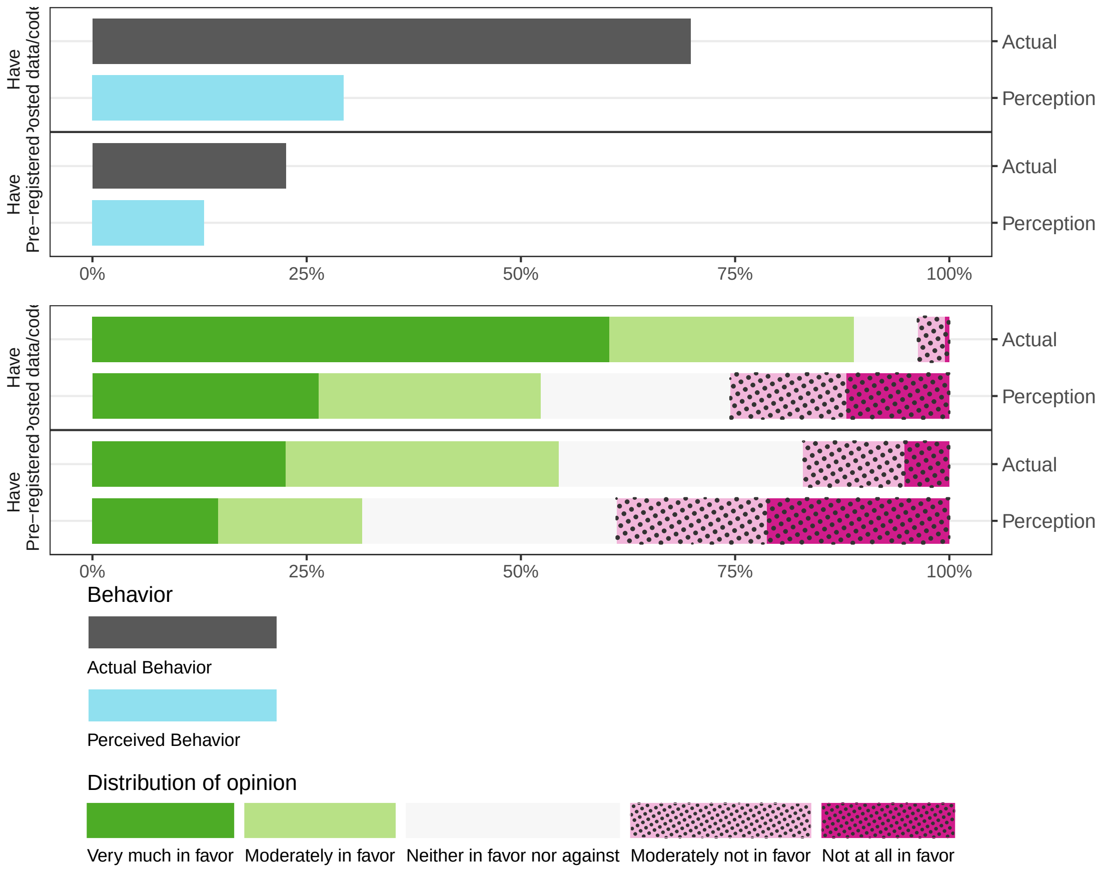

Supplementary Figure 27: **Perceived and Actual Behavior and Support for Open Science among Published Authors - Wave 1** The chart shows differences between perceived and actual behavior (top panel) and support (bottom panel) for two practices: posting data or code online and pre-registering hypotheses or analyses. The sample is restricted to published authors who responded to the first survey wave. In the first panel, the first solid black bar shows the proportion of researchers who have posted data, using the responses elicited from our survey. The second blue bar below shows the fraction of researchers in their field they believe have posted data. The third and fourth bars show analogous information for the practice of pre-registration. In the second panel, the first bar show the distribution of support for posting data or code online, constructed using the responses elicited from the sample mentioned above. Colors for the bars showing perceived and stated support indicate the degree of support, with green indicating more and red indicating less support. The second bar shows the perceived distribution of support for posting data or code online among the sample mentioned above. This is constructed by asking individuals what percentage of researchers in their field they believe fall into each opinion category, and then averaging over their responses. The final two bars show analogous information for the practice of preregistration.

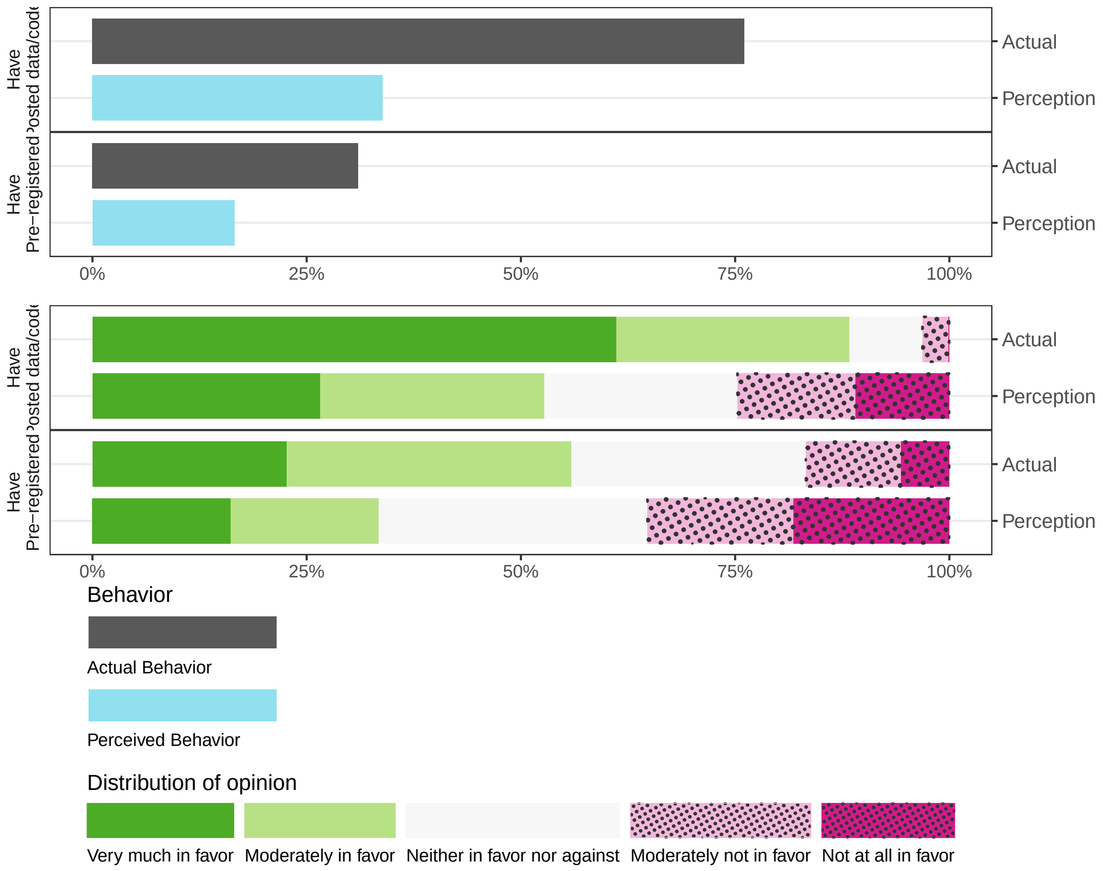

Supplementary Figure 28: **Perceived and Actual Support for Open Science among Published Authors - Wave 2** The chart shows differences between perceived and actual behavior (top panel) and support (bottom panel) for two practices: posting data or code online and pre-registering hypotheses or analyses. The sample is restricted to published authors who responded to the second survey wave. In the first panel, the first solid black bar shows the proportion of researchers who have posted data, using the responses elicited from our survey. The second blue bar below shows the fraction of researchers in their field they believe have posted data. The third and fourth bars show analogous information for the practice of pre-registration. In the second panel, the first bar show the distribution of support for posting data or code online, constructed using the responses elicited from the sample mentioned above. Colors for the bars showing perceived and stated support indicate the degree of support, with green indicating more and red indicating less support. The second bar shows the perceived distribution of support for posting data or code online among the sample mentioned above. This is constructed by asking individuals what percentage of researchers in their field they believe fall into each opinion category, and then averaging over their responses. The final two bars show analogous information for the practice of preregistration.

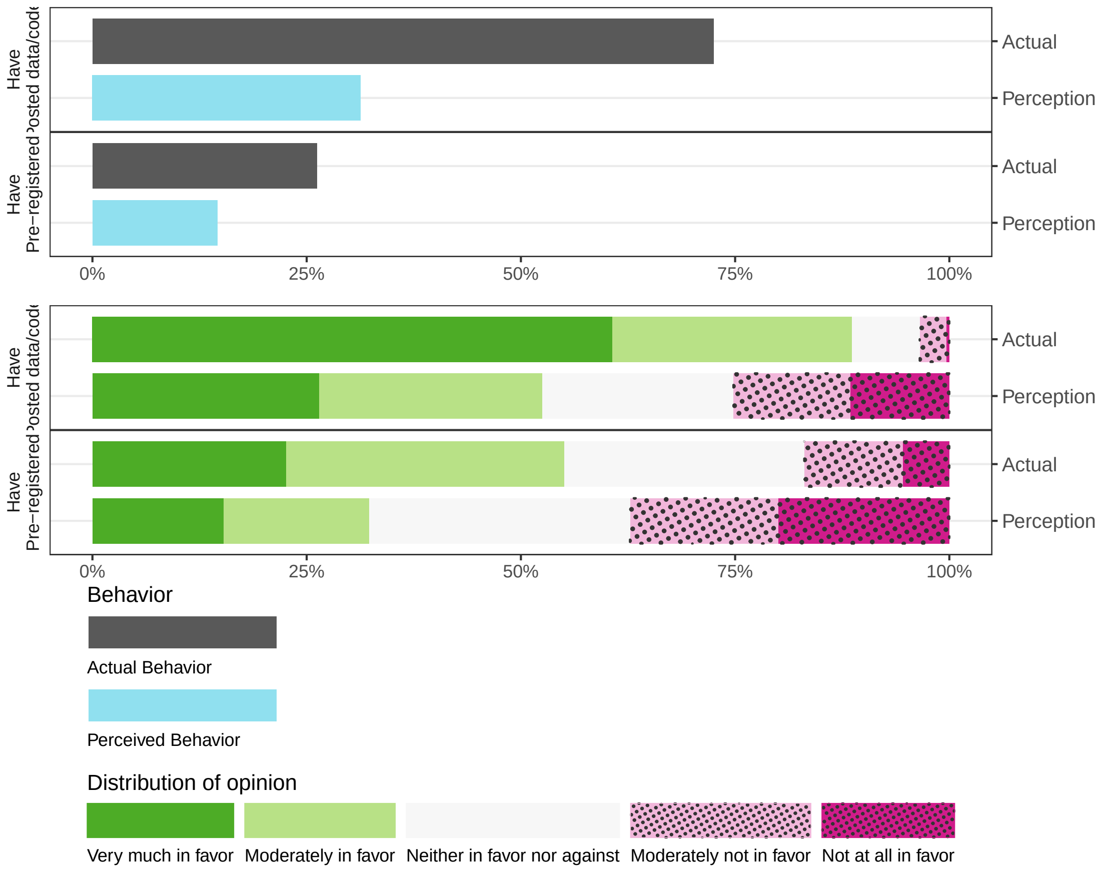

Supplementary Figure 29: **Perceived and Actual Support for Open Science among Published Authors - Whole Sample, responses averaged** The chart shows differences between perceived and actual behavior (top panel) and support (bottom panel) for two practices: posting data or code online and pre-registering hypotheses or analyses. The sample includes published authors any survey wave, with responses averaged for those published authors who responded to both waves. In the first panel, the first solid black bar shows the proportion of researchers who have posted data, using the responses elicited from our survey. The second blue bar below shows the fraction of researchers in their field they believe have posted data. The third and fourth bars show analogous information for the practice of pre-registration. In the second panel, the first bar show the distribution of support for posting data or code online, constructed using the responses elicited from the sample mentioned above. Colors for the bars showing perceived and stated support indicate the degree of support, with green indicating more and red indicating less support. The second bar shows the perceived distribution of support for posting data or code online among the sample mentioned above. This is constructed by asking individuals what percentage of researchers in their field they believe fall into each opinion category, and then averaging over their responses. The final two bars show analogous information for the practice of preregistration.

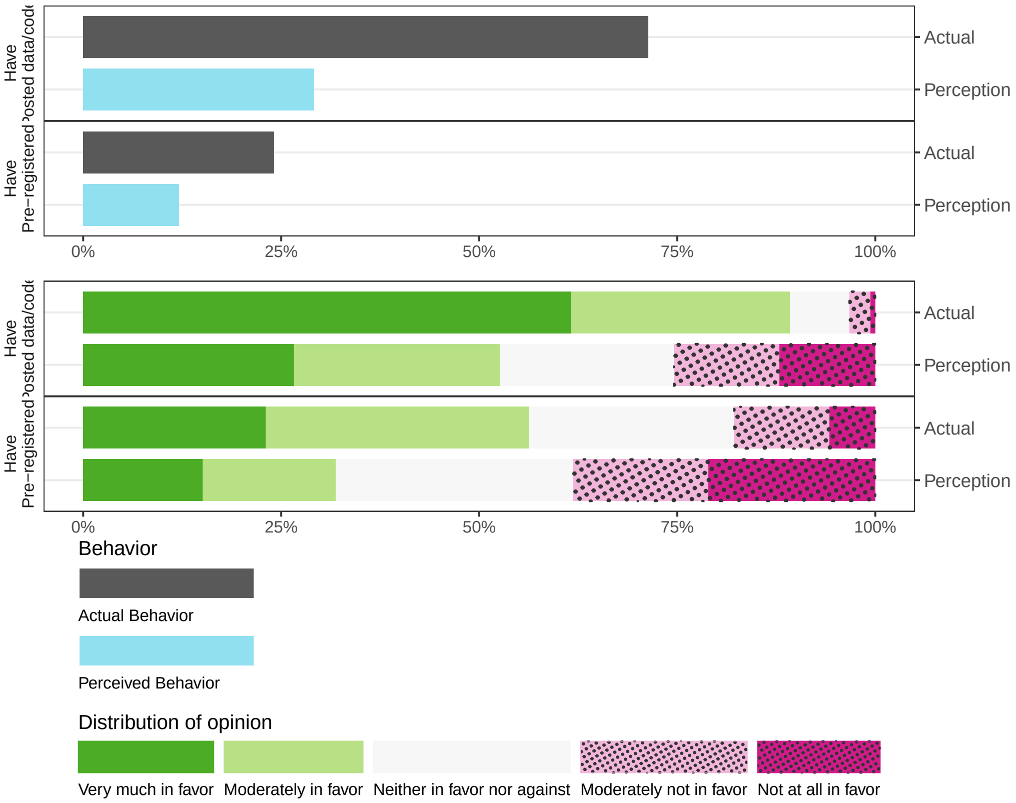

Supplementary Figure 30: **Both Wave Completers Wave 1 - Perceived and Actual Support for and Reported Adoption of Open Science among Published Authors.** The chart shows differences between perceived and actual behavior (top panel) and support (bottom panel) for two practices: posting data or code online and pre-registering hypotheses or analyses. The sample is restricted to published authors who responded to both survey waves, and shows their response the first survey wave. In the first panel, the first solid black bar shows the proportion of researchers who have posted data, using the responses elicited from our survey. The second blue bar below shows the fraction of researchers in their field they believe have posted data. The third and fourth bars show analogous information for the practice of pre-registration. In the second panel, the first bar shows the distribution of support for posting data or code online, constructed using the responses elicited from the sample mentioned above. Colors for the bars showing perceived and stated support indicate the degree of support, with green indicating more and red indicating less support. The second bar shows the perceived distribution of support for posting data or code online among the sample mentioned above. This is constructed by asking individuals what percentage of researchers in their field they believe fall into each opinion category, and then averaging over their responses. The final two bars show analogous information for the practice of preregistration.

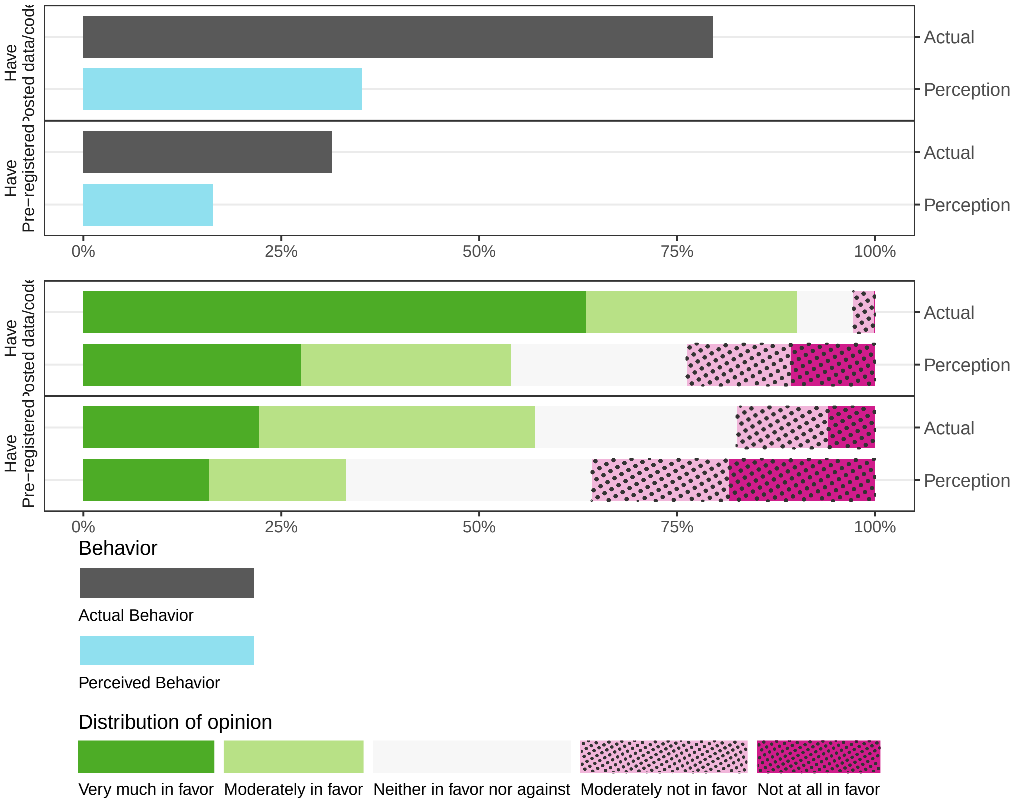

Supplementary Figure 31: **Both Wave Completers Wave 2 - Perceived and Actual Support for Open Science among Published Authors.** The chart shows differences between perceived and actual behavior (top panel) and support (bottom panel) for two practices: posting data or code online and pre-registering hypotheses or analyses. The sample is restricted to published authors who responded to both survey waves, and shows these Authors' responses to the second survey wave. In the first panel, the first solid black bar shows the proportion of researchers who have posted data, using the responses elicited from our survey. The second blue bar below shows the fraction of researchers in their field they believe have posted data. The third and fourth bars show analogous information for the practice of pre-registration. In the second panel, the first bar shows the distribution of support for posting data or code online, constructed using the responses elicited from the sample mentioned above. Colors for the bars showing perceived and stated support indicate the degree of support, with green indicating more and red indicating less support. The second bar shows the perceived distribution of support for posting data or code online among the sample mentioned above. This is constructed by asking individuals what percentage of researchers in their field they believe fall into each opinion category, and then averaging over their responses. The final two bars show analogous information for the practice of preregistration.

SI 2.2.1 Attitudes Awareness Behavior, Description and Prescriptive Norms, Heterogeneity

Supplementary Table 31: Differences in Sub Indices across Disciplines

	Attitude		Awareness		Behavior		Descriptive Norms		Prescriptive Norms	
	(1)	(2)	(3)	(4)	(5)	(6)	(7)	(8)	(9)	(10)
Economics	0.086***	0.086***	0.047***	0.035***	0.177***	0.146***	0.151***	0.162***	0.116***	0.113***
	(0.01)	(0.01)	(0.01)	(0.01)	(0.01)	(0.02)	(0.01)	(0.01)	(0.01)	(0.01)
Political Science	0.062***	0.059***	0.084***	0.047***	0.240***	0.234***	0.162***	0.177***	0.080***	0.071***
	(0.01)	(0.01)	(0.01)	(0.01)	(0.01)	(0.02)	(0.01)	(0.01)	(0.01)	(0.01)
Psychology	0.098***	0.093***	0.088***	0.046***	0.230***	0.285***	0.118***	0.136***	0.078***	0.075***
	(0.01)	(0.01)	(0.01)	(0.01)	(0.02)	(0.02)	(0.01)	(0.01)	(0.01)	(0.01)
Years since started PhD		-0.001***		-0.002***		0.000		0.000		0.000
		(0)		(0)		(0)		(0)		(0)
Male		0.011*		0.016**		0.107***		0.013*		0.022***
		(0.01)		(0.01)		(0.01)		(0.01)		(0.01)
Tenured		0.000		0.028**		0.123***		0.018		-0.005
		(0.01)		(0.01)		(0.02)		(0.01)		(0.01)
Leadership Position		-0.008		0.003		-0.007		0.005		-0.009
		(0.01)		(0.01)		(0.01)		(0.01)		(0.01)
USA and Canada		-0.025**		0.006		-0.062***		0.007		0.002
		(0.01)		(0.01)		(0.02)		(0.01)		(0.01)
Constant	0.759***	0.799***	0.854***	0.923***	0.230***	0.213***	0.144***	0.130***	0.487***	0.494***
	(0)	(0.01)	(0.01)	(0.02)	(0.01)	(0.03)	(0)	(0.02)	(0)	(0.01)
Observations	3,178	1,975	1,981	3,179	1,981	3,163	1,978	3,172	1,980	3,153

This table presents regression coefficients and standard errors from ordinary least squares regressions. The outcome variable in each regression is one of the sub indices described in Supplementary Table 7. The covariates are indicator variables for the discipline of the respondent. In odd numbered specifications no other control variables are included. In even numbered specifications individual-level covariates are included. The omitted discipline in the regressions is Sociology. Significance stars are indicated for standard errors computed using a multiple testing adjustment to address risk of false positives. In particular, we use the False Discovery Rate (FDR) adjustment in [11] and discussed in [12]. * indicates significance at the 10% level, ** indicates significance at the 5% level and *** indicates significance at the 1% level from a two-sided t-test.

Supplementary Table 32: Differences in Sub Indices by Author Type

	Attitude		Awareness		Behavior		Descriptive Norms		Prescriptive Norms	
	(1)	(2)	(3)	(4)	(5)	(6)	(7)	(8)	(9)	(10)
Published Author	-0.004	0.021**	0.018**	0.007	0.172***	0.134***	0.012*	0.000	0.002	0.013
	(0)	(0.01)	(0.01)	(0.01)	(0.01)	(0.02)	(0.01)	(0.01)	(0.01)	(0.01)
Years since started PhD		-0.002***		-0.002***		-0.004***		-0.001*		-0.001*
		(0)		(0)		(0)		(0)		(0)
Male		0.014**		0.018**		0.099***		0.035***		0.034***
		(0.01)		(0.01)		(0.01)		(0.01)		(0.01)
Tenured		-0.006		0.028**		0.074***		0.033**		-0.002
		(0.01)		(0.01)		(0.02)		(0.01)		(0.01)
Leadership Position		-0.009		0.003		0.000		0.000		-0.011
		(0.01)		(0.01)		(0.01)		(0.01)		(0.01)
USA and Canada		-0.024**		0.005		-0.037		0.000		0.002
		(0.01)		(0.01)		(0.02)		(0.01)		(0.01)
Constant	0.820***	0.857***	0.900***	0.956***	0.315***	0.362***	0.246***	0.253***	0.553***	0.555***
	(0)	(0.01)	(0.01)	(0.01)	(0.01)	(0.03)	(0)	(0.02)	(0)	(0.01)
Observations	3,178	1,975	1,981	3,179	1,981	3,163	1,978	3,172	1,980	3,153

This table presents regression coefficients and standard errors from ordinary least squares regressions. The outcome variable in each regression is one of the sub indices described in Supplementary Table 7. The covariates are indicator variables for the whether the respondent has published in one of the journals in Supplementary Tables 2 through 5. In odd numbered specifications no other control variables are included. In even numbered specifications individual-level covariates are included. The omitted career stage in the regressions is PhD students. Significance stars are indicated for standard errors computed using a multiple testing adjustment to address risk of false positives. In particular, we use the False Discovery Rate (FDR) adjustment in [11] and discussed in [12]. * indicates significance at the 10% level, ** indicates significance at the 5% level and *** indicates significance at the 1% level from a two-sided t-test.

Supplementary Table 33: Differences in Sub Indices across Disciplines and Author type

	Attitude		Awareness		Behavior		Descriptive Norms		Prescriptive Norms	
	(1)	(2)	(3)	(4)	(5)	(6)	(7)	(8)	(9)	(10)
Published Author	-0.006	0.016	0.03	0.007	0.129***	0.117***	0.003	-0.008	-0.012	-0.006
	(0.01)	(0.01)	(0.02)	(0.02)	(0.02)	(0.03)	(0.01)	(0.01)	(0.01)	(0.01)
Economics	0.095***	0.091***	0.052***	0.032*	0.124***	0.127***	0.147***	0.158***	0.109***	0.101***
	(0.01)	(0.01)	(0.02)	(0.02)	(0.02)	(0.02)	(0.01)	(0.01)	(0.01)	(0.01)
Political Science	0.045***	0.039***	0.085***	0.045***	0.171***	0.198***	0.154***	0.171***	0.076***	0.062***
	(0.01)	(0.01)	(0.02)	(0.02)	(0.02)	(0.02)	(0.01)	(0.01)	(0.01)	(0.01)
Psychology	0.103***	0.101***	0.103***	0.052***	0.265***	0.317***	0.127***	0.143***	0.072***	0.062***
	(0.01)	(0.01)	(0.01)	(0.02)	(0.02)	(0.02)	(0.01)	(0.01)	(0.01)	(0.01)
Published Author:Economics	-0.019	-0.01	-0.015	0.007	0.099***	0.048	0.009	0.011	0.017	0.029
	(0.01)	(0.02)	(0.02)	(0.02)	(0.03)	(0.03)	(0.01)	(0.02)	(0.01)	(0.02)
Published Author:Political Science	0.034**	0.04**	-0.006	0.003	0.113***	0.064*	0.015	0.014	0.009	0.021
	(0.01)	(0.02)	(0.02)	(0.02)	(0.03)	(0.03)	(0.01)	(0.02)	(0.01)	(0.02)
Published Author:Psychology	-0.016	-0.028	-0.038	-0.018	-0.075**	-0.107**	-0.025	-0.02	0.016	0.031
	(0.01)	(0.02)	(0.02)	(0.02)	(0.03)	(0.04)	(0.02)	(0.02)	(0.01)	(0.02)
Years since started PhD		-0.001***		-0.002**		-0.003**		0.000		-0.001
		(0)		(0)		(0)		(0)		(0)
Male		0.01		0.016*		0.102***		0.014		0.021***
		(0.01)		(0.01)		(0.01)		(0.01)		(0.01)
Tenured		-0.013		0.024*		0.064***		0.017		-0.009
		(0.01)		(0.01)		(0.02)		(0.01)		(0.01)
Leadership Position		-0.007		0.003		-0.004		0.004		-0.008
		(0.01)		(0.01)		(0.01)		(0.01)		(0.01)
USA and Canada		-0.021*		0.007		-0.03		0.005		0.007
		(0.01)		(0.01)		(0.02)		(0.01)		(0.01)
Constant	0.762***	0.795***	0.841***	0.921***	0.176***	0.182***	0.143***	0.133***	0.492***	0.498***
	(0.01)	(0.01)	(0.01)	(0.02)	(0.01)	(0.03)	(0.01)	(0.02)	(0.01)	(0.01)
Observations	3,178	1,975	1,981	3,179	1,981	3,163	1,978	3,172	1,980	3,153

This table presents regression coefficients and standard errors from ordinary least squares regressions. The outcome variable in each regression is one of the sub indices described in Supplementary Table 7. The covariates are indicator variables for the discipline and author type of the respondent. In odd numbered specifications no other control variables are included. In even numbered specifications individual-level covariates are included. The omitted discipline in the regressions is Sociology, and the omitted career stage is PhD students. Coefficients on disciplines not interacted with published authors are effects for PhD students in these disciplines. Significance stars are indicated for standard errors computed using a multiple testing adjustment to address risk of false positives. In particular, we use the False Discovery Rate (FDR) adjustment in [11] and discussed in [12]. * indicates significance at the 10% level, ** indicates significance at the 5% level and *** indicates significance at the 1% level from a two-sided t-test.

SI 2.3 Broad Indice Heterogeneity Results

Supplementary Table 34: Differences in Broad Indices across Disciplines

	Personal support (no norms)		Norms		Overall (includes norms)		Trustworthiness of literature	
	(1)	(2)	(3)	(4)	(5)	(6)	(7)	(8)
Economics	0.103***	0.089***	0.135***	0.138***	0.095***	0.094***	0.015	0.018
	(0.01)	(0.01)	(0.01)	(0.01)	(0.01)	(0.01)	(0.01)	(0.01)
Political Science	0.129***	0.113***	0.122***	0.124***	0.113***	0.114***	-0.038***	-0.037***
	(0.01)	(0.01)	(0.01)	(0.01)	(0.01)	(0.01)	(0.01)	(0.01)
Psychology	0.138***	0.141***	0.100***	0.107***	0.114***	0.127***	-0.051***	-0.046***
	(0.01)	(0.01)	(0.01)	(0.01)	(0.01)	(0.01)	(0.01)	(0.01)
Years since started PhD		-0.001**		0.000		0.000		0.000
		(0)		(0)		(0)		(0)
Male		0.045***		0.018***		0.030***		-0.009
		(0.01)		(0.01)		(0.01)		(0.01)
Tenured		0.050***		0.007		0.034***		0.060***
		(0.01)		(0.01)		(0.01)		(0.01)
Leadership Position		-0.004		-0.003		-0.001		-0.007
		(0.01)		(0.01)		(0.01)		(0.01)
USA and Canada		-0.027**		0.004		-0.023**		-0.049***
		(0.01)		(0.01)		(0.01)		(0.01)
Constant	0.614***	0.645***	0.314***	0.311***	0.465***	0.459***	0.629***	0.663***
	(0.01)	(0.01)	(0)	(0.01)	(0)	(0.01)	(0.01)	(0.02)
Observations	3,179	1,981	3,173	1,980	3,179	1,981	3,178	1,981

This table presents regression coefficients and standard errors from ordinary least squares regressions. The outcome variable in each regression is one of the broad indices described in Supplementary Table 7. The covariates are indicator variables for the discipline of the respondent. In odd numbered specifications no other control variables are included. In even numbered specifications individual-level covariates are included. The omitted discipline in the regressions is Sociology. Significance stars are indicated for standard errors computed using a multiple testing adjustment to address risk of false positives. In particular, we use the False Discovery Rate (FDR) adjustment in [11] and discussed in [12]. * indicates significance at the 10% level, ** indicates significance at the 5% level and *** indicates significance at the 1% level from a two-sided t-test.

Supplementary Table 35: Differences in Broad Indices by Author Type

	Personal support (no norms)		Norms		Overall (includes norms)		Trustworthiness of literature	
	(1)	(2)	(3)	(4)	(5)	(6)	(7)	(8)
Published Author	0.062***	0.054***	0.007	0.007	0.045***	0.041***	0.047***	0.021
	(0.01)	(0.01)	(0)	(0.01)	(0)	(0.01)	(0.01)	(0.01)
Years since started PhD		-0.003***		-0.001**		-0.001***		-0.001
		(0)		(0)		(0)		(0)
Male		0.044***		0.035***		0.033***		-0.003
		(0.01)		(0.01)		(0.01)		(0.01)
Tenured		0.032***		0.015		0.022**		0.056***
		(0.01)		(0.01)		(0.01)		(0.01)
Leadership Position		-0.002		-0.006		-0.001		-0.007
		(0.01)		(0.01)		(0.01)		(0.01)
USA and Canada		-0.019		0.000		-0.018		-0.041***
		(0.01)		(0.01)		(0.01)		(0.01)
Constant	0.679***	0.725***	0.399***	0.403***	0.524***	0.539***	0.589***	0.633***
	(0)	(0.01)	(0)	(0.01)	(0)	(0.01)	(0)	(0.02)
Observations	3,179	1,981	3,173	1,980	3,179	1,981	3,178	1,981

This table presents regression coefficients and standard errors from ordinary least squares regressions. The outcome variable in each regression is one of the broad indices described in Supplementary Table 7. The covariates are indicator variables for the whether the respondent has published in one of the journals in Supplementary Tables 2 through 5. In odd numbered specifications no other control variables are included. In even numbered specifications individual-level covariates are included. The omitted career stage in the regressions is PhD students. Significance stars are indicated for standard errors computed using a multiple testing adjustment to address risk of false positives. In particular, we use the False Discovery Rate (FDR) adjustment in [11] and discussed in [12]. * indicates significance at the 10% level, ** indicates significance at the 5% level and *** indicates significance at the 1% level from a two-sided t-test.

Supplementary Table 36: Differences in Broad Indices across Disciplines and Author type

	Personal support (no norms)		Norms		Overall (includes norms)		Trustworthiness of literature	
	(1)	(2)	(3)	(4)	(5)	(6)	(7)	(8)
Published Author	0.050***	0.047***	-0.006	-0.008	0.038***	0.037***	0.027*	0.016
	(0.01)	(0.01)	(0.01)	(0.01)	(0.01)	(0.01)	(0.01)	(0.02)
Economics	0.090***	0.084***	0.128***	0.130***	0.087***	0.091***	-0.008	0.002
	(0.01)	(0.01)	(0.01)	(0.01)	(0.01)	(0.01)	(0.01)	(0.01)
Political Science	0.100***	0.094***	0.115***	0.115***	0.097***	0.103***	-0.056***	-0.049***
	(0.01)	(0.01)	(0.01)	(0.01)	(0.01)	(0.01)	(0.01)	(0.01)
Psychology	0.156***	0.157***	0.100***	0.104***	0.125***	0.138***	-0.049***	-0.040***
	(0.01)	(0.01)	(0.01)	(0.01)	(0.01)	(0.01)	(0.01)	(0.01)
Published Author:Economics	0.022	0.014	0.016	0.019	0.011	0.007	0.046**	0.038
	(0.01)	(0.02)	(0.01)	(0.02)	(0.01)	(0.02)	(0.02)	(0.02)
Published Author:Political Science	0.048***	0.036*	0.016	0.021	0.026**	0.020	0.031	0.023
	(0.01)	(0.02)	(0.01)	(0.01)	(0.01)	(0.01)	(0.02)	(0.02)
Published Author:Psychology	-0.042**	-0.051**	-0.002	0.006	-0.024*	-0.033*	-0.001	-0.022
	(0.02)	(0.02)	(0.01)	(0.02)	(0.01)	(0.02)	(0.02)	(0.02)
Years since started PhD		-0.002***		0.000		-0.001		-0.001
		(0)		(0)		(0)		(0)
Male		0.042***		0.018***		0.028***		-0.010
		(0.01)		(0.01)		(0.01)		(0.01)
Tenured		0.025**		0.004		0.016		0.046***
		(0.01)		(0.01)		(0.01)		(0.01)
Leadership Position		-0.003		-0.003		0.000		-0.008
		(0.01)		(0.01)		(0.01)		(0.01)
USA and Canada		-0.015		0.005		-0.014		-0.041***
		(0.01)		(0.01)		(0.01)		(0.01)
Constant	0.594***	0.633***	0.316***	0.315***	0.449***	0.449***	0.618***	0.660***
	(0.01)	(0.01)	(0)	(0.01)	(0)	(0.01)	(0.01)	(0.02)
Observations	3,179	1,981	3,173	1,980	3,179	1,981	3,178	1,981

This table presents regression coefficients and standard errors from ordinary least squares regressions. The outcome variable in each regression is one of the broad indices described in Supplementary Table 7. The covariates are indicator variables for the discipline and author type of the respondent. In odd-numbered specifications no other control variables are included. In even-numbered specifications individual-level covariates are included. The omitted discipline in the regressions is Sociology, and the omitted career stage is PhD students. Coefficients on disciplines not interacted with published authors are effects for PhD students in these disciplines. Significance stars are indicated for standard errors computed using a multiple testing adjustment to address risk of false positives. In particular, we use the False Discovery Rate (FDR) adjustment in [11] and discussed in [12]. * indicates significance at the 10% level, ** indicates significance at the 5% level and *** indicates significance at the 1% level from a two-sided t-test.

References

[1] Swanson, N., Christensen, G., Littman, R., Birke, D., Miguel, E., Paluck, E. L. & Wang, Z. *Research transparency is on the rise in economics* in *AEA Papers and Proceedings* **110** (2020), 61–65.

[2] Hardwicke, T. E., Wallach, J. D., Kidwell, M. C., Bendixen, T., Crüwell, S. & Ioannidis, J. P. An empirical assessment of transparency and reproducibility-related research practices in the social sciences (2014–2017). *Royal Society open science* **7**, 190806 (2020).

[3] Hardwicke, T. E., Thibault, R. T., Kosie, J. E., Wallach, J. D., Kidwell, M. C. & Ioannidis, J. P. Estimating the prevalence of transparency and reproducibility-related research practices in psychology (2014–2017). *Perspectives on Psychological Science* **17**, 239–251 (2022).

[4] Imai, K., Ratkovic, M. *et al.* Estimating treatment effect heterogeneity in randomized program evaluation. *The Annals of Applied Statistics* **7**, 443–470 (2013).

[5] Ning, Y., Sida, P. & Imai, K. Robust estimation of causal effects via a high-dimensional covariate balancing propensity score. *Biometrika* **107**, 533–554 (2020).

[6] Ferri-García, R. & Rueda, M. d. M. Propensity score adjustment using machine learning classification algorithms to control selection bias in online surveys. *PloS one* **15**, e0231500 (2020).

[7] Mullainathan, S. & Spiess, J. Machine learning: an applied econometric approach. *Journal of Economic Perspectives* **31**, 87–106 (2017).

[8] Pirracchio, R., Petersen, M. L. & Van Der Laan, M. Improving propensity score estimators' robustness to model misspecification using super learner. *American journal of epidemiology* **181**, 108–119 (2015).

[9] Li, D., Raymond, L. R. & Bergman, P. *Hiring as Exploration* tech. rep. (National Bureau of Economic Research, 2020).

[10] Fischer, C. *et al.* Evaluating the performance of the breast cancer genetic risk models BOADICEA, IBIS, BRCAPRO and Claus for predicting BRCA1/2 mutation carrier probabilities: a study based on 7352 families from the German Hereditary Breast and Ovarian Cancer Consortium. *Journal of medical genetics* **50**, 360–367 (2013).

[11] Benjamini, Y., Krieger, A. M. & Yekutieli, D. Adaptive linear step-up procedures that control the false discovery rate. *Biometrika* **93**, 491–507 (2006).

[12] Anderson, M. L. Multiple inference and gender differences in the effects of early intervention: A reevaluation of the Abecedarian, Perry Preschool, and Early Training Projects. *Journal of the American statistical Association* **103**, 1481–1495 (2008).
