# Supplementary material for: Survey of open science practices and attitudes in the social sciences
Source: Nat Commun. 2023 Sep 5;14:5401. doi: 10.1038/s41467-023-41111-1 (PMC10480148; doi:10.1038/s41467-023-41111-1)
Supplement: Supplementary file 2 — Reporting Summary [file 41467_2023_41111_MOESM2_ESM.pdf]

Corresponding author(s): Edward Miguel

Last updated by author(s): 3/31/2023

Reporting Summary

Nature Portfolio wishes to improve the reproducibility of the work that we publish. This form provides structure for consistency and transparency in reporting. For further information on Nature Portfolio policies, see our Editorial Policies and the Editorial Policy Checklist.

Statistics

For all statistical analyses, confirm that the following items are present in the figure legend, table legend, main text, or Methods section.

n/a	Confirmed	
☐	☒	The exact sample size (*n*) for each experimental group/condition, given as a discrete number and unit of measurement
☐	☒	A statement on whether measurements were taken from distinct samples or whether the same sample was measured repeatedly
☐	☒	The statistical test(s) used AND whether they are one- or two-sided<br>*Only common tests should be described solely by name; describe more complex techniques in the Methods section.*
☐	☒	A description of all covariates tested
☐	☒	A description of any assumptions or corrections, such as tests of normality and adjustment for multiple comparisons
☐	☒	A full description of the statistical parameters including central tendency (e.g. means) or other basic estimates (e.g. regression coefficient) AND variation (e.g. standard deviation) or associated estimates of uncertainty (e.g. confidence intervals)
☐	☒	For null hypothesis testing, the test statistic (e.g. *F*, *t*, *r*) with confidence intervals, effect sizes, degrees of freedom and *P* value noted<br>*Give P values as exact values whenever suitable.*
☒	☐	For Bayesian analysis, information on the choice of priors and Markov chain Monte Carlo settings
☒	☐	For hierarchical and complex designs, identification of the appropriate level for tests and full reporting of outcomes
☐	☒	Estimates of effect sizes (e.g. Cohen's *d*, Pearson's *r*), indicating how they were calculated

Our web collection on statistics for biologists contains articles on many of the points above.

Software and code

Policy information about availability of computer code

Data collection	Data was primarily collected in Qualtrics, with additional data collected via web-scraping using R (4.0.2)
Data analysis	Analysis was conducted in R version 4.0.2. No custom algorithms were used. Analysis consists of means and regression analysis using base R, and the CBPS: Covariate Balancing Propensity Score version 0.21 and Superlearner (for ensemble machine learning, version 2.0-26) packages. Analysis also used cvAUC version: 1.1.0, cobalt (Version 4.3.0, Build Date: 2021-02-20 05:50:20 UTC), glmnet 4.1-1, , and arm (Version 1.11-2, built: 2020-7-27).

For manuscripts utilizing custom algorithms or software that are central to the research but not yet described in published literature, software must be made available to editors and reviewers. We strongly encourage code deposition in a community repository (e.g. GitHub). See the Nature Portfolio guidelines for submitting code & software for further information.

Data

Policy information about availability of data

All manuscripts must include a data availability statement. This statement should provide the following information, where applicable:

- Accession codes, unique identifiers, or web links for publicly available datasets
- A description of any restrictions on data availability
- For clinical datasets or third party data, please ensure that the statement adheres to our policy

The de-identified data generated in this study have been deposited in the Open Science Framework at https://osf.io/zn8u2/ and can be freely accessed. The raw

respondent-provided subfield data are protected and are not available to protect anonymity. Data come from our survey, as well as third party databases including AsPredicted, Harvard Dataverse, the American Economics Association RCT Registry, Evidence in Governance and Politics Registry, and Open Science Framework.

We have not included the raw data for the hand audit completed by our team, as this contains information that could be used to identify respondents in our survey. The code and output data used in the analysis are included in the replication materials.

Human research participants

Policy information about studies involving human research participants and Sex and Gender in Research.

Reporting on sex and gender

Participants were asked "What is your gender identity?" (Male, female, transgender, genderqueer, or other). This variable is not used in the main analysis. Limited analysis in the supplementary materials report gender differences, as pre-specified.

Population characteristics

See Behavioural & social sciences study design

Recruitment

Participants were contacted via e-mail. Overall, 6,620 individuals were invited across the two waves of surveys, of whom 6,570 were actually contacted (e-mails did not bounce). In terms of representativeness, the audit suggests that individuals with a more empirical orientation were more likely to respond to the survey. However, after controlling for observable characteristics such as empirical orientation there is no difference in behavior between those who responded to the survey and those who did not.

The fact that the response rate is less than 100% implies that there is the possibility that the individuals who responded to the survey may not be representative of the invited population. In the paper, we conduct numerous adjustments to attempt to correct for this kind of selection and find our results do not meaningfully change. In addition, there is the possibility of survey response bias. To allay concerns about this, we conduct manual audits of some behavior and find a high degree of alignment between stated and observed behavior.

Ethics oversight

The research, including both the survey and behavioral audit data, complied with all relevant ethical regulations including obtaining informed consent from each participant, and was approved by the Princeton Institutional Review Board (Protocol #11972) [This statement appears in the manuscript as well.]

Note that full information on the approval of the study protocol must also be provided in the manuscript.

Field-specific reporting

Please select the one below that is the best fit for your research. If you are not sure, read the appropriate sections before making your selection.

☐ Life sciences ☒ Behavioural & social sciences ☐ Ecological, evolutionary & environmental sciences

For a reference copy of the document with all sections, see nature.com/documents/nr-reporting-summary-flat.pdf

Behavioural & social sciences study design

All studies must disclose on these points even when the disclosure is negative.

Study description

This is a descriptive quantitative study. Using a survey, this study provides an assessment of attitudes towards, lifetime usage of, and perceived norms regarding open science practices within a broadly representative sample of scholars from four major social science disciplines: economics, political science, psychology, and sociology. We confirm the representativeness of our survey by searching for study materials, the discipline, and sub-discipline of non-respondents. We also document that reported lifetime adoption of open science practices doubled from 49% in 2010 to 87% a decade later. We assess this with self-report and confirm the self-report by searching for study materials online.

Research sample

We randomly drew the sample for the first 2 waves of the survey from the complete set of authors who had published within a range of 3 years (2014-2016) in 10 of the most-cited journals for each of four disciplines: economics, political science, psychology, and sociology. We also drew from the complete set of PhD students enrolled in each discipline in the top-20 North American universities during the fall of 2017. We did not collect the age of participants; 42% of sample participants identify as women. Within the practical and budget constraints of the project, we opted to focus on a sample of scholars who (in our view) were likely to be active and influential researchers, regardless of demographic characteristics.

Sampling strategy

Published Authors and PhD students were randomly selected from the pool described above. We were able to successfully contact 6,058 researcher via email. Of the contacted researchers we had 3,257 respondents and 2,856 non-respondents. We based our pre-registered power calculations on conservative estimates of response rates from prior transparency survey and our own pilot. We conducted power calculations expecting roughly equal numbers in each discipline. These assumptions yields an expected final sample size between 3,000 and 4,000, with 3,200 as our best guess.

Data collection

Data was collected via the online survey software Qualtrics—subjects were invited to participate in the study via e-mail and provided data independently via the Internet. Since subjects responded to the survey on their own over the Internet, we have no way of knowing if anyone else was present when they completed the survey. The researchers were not blinded to treatment status, but

	researchers were not present when subjects participated and all invitations to participate were identical regardless of treatment status. To verify self-reported behaviors and to examine whether survey respondents were representative, we conducted automated (web-scraping) and manual web verification of participants' self-reported behaviors as a check on reliability
Timing	The survey was administered twice: once between April and August 2018 (Wave 1) and once between March and July 2020 (Wave 2).
Data exclusions	Data is limited to participants who were invited to both waves of the survey.
Non-participation	53% of individuals contacted responded to at least one wave of surveys. Figure 3 in the Supplementary Materials presents the overall response rate by field, which ranged from 47% in Psychology to 60% in Political Science. PhD students responded at or above 50% in every field, while published authors (who had predominantly completed their doctoral training) responded at somewhat lower rates.
Randomization	Sample selection was drawn from the pool of scholars (see Research sample) using base R's pseudo-random-number generator, with the seed of 42, which can be seen in section 5.6 of the pre-analysis plan. The pre-analysis plan includes a set of experimental interventions that we included as part of our survey. The results of these interventions are not included in this paper as we plan to include them in a later, separate publication.

Reporting for specific materials, systems and methods

We require information from authors about some types of materials, experimental systems and methods used in many studies. Here, indicate whether each material, system or method listed is relevant to your study. If you are not sure if a list item applies to your research, read the appropriate section before selecting a response.

Materials & experimental systems

n/a	Involved in the study	
☒	☐	Antibodies
☒	☐	Eukaryotic cell lines
☒	☐	Palaeontology and archaeology
☒	☐	Animals and other organisms
☒	☐	Clinical data
☒	☐	Dual use research of concern

Methods

n/a	Involved in the study	
☒	☐	ChIP-seq
☒	☐	Flow cytometry
☒	☐	MRI-based neuroimaging
